# Synthesis of 1,4-Benzodiazepines via Intramolecular C–N Bond Coupling and Ring Opening of Azetidines

**DOI:** 10.3390/molecules30092014

**Published:** 2025-04-30

**Authors:** Xin-Ming Xu, Sen Chen, Shao-Lei Duan, Xiang-Min Wang, Qian Liu, Kai Sun

**Affiliations:** 1 School of Chemistry and Chemical Engineering, Yantai University, Yantai 264005, China; 2 School of Life Scienses, Yantai University, Yantai 264005, China

**Keywords:** 1,4-benzodiazepine, cross-coupling reaction, ring-opening reaction, *N*-methylation, azetidine

## Abstract

A facile and efficient synthesis of functionalized 1,4-benzodiazepine derivatives under mild conditions was developed. The CuI/*N*,*N*-dimethylglycine-catalyzed intramolecular cross-coupling reaction of 1-(2-bromobenzyl)azetidine-2-carboxamides proceeded smoothly under mild conditions to provide 1,4,9,10a-tetrahydroazeto[1,2-*a*]benzo[e][1,4]diazepin-10(2*H*)-ones. The resulting azetidine-fused 1,4-benzodiazepine compounds underwent consecutive *N*-methylation with methyl triflate and the opening of the four-membered heterocyclic ring by NaN_3_, KCN and PhSNa to produce diverse 1,4-benzodiazepine derivatives in good to excellent yields. Upon treatment with methyl chloroformate, on the other hand, the 1,4,9,10a-tetrahydroazeto[1,2-*a*]benzo[e][1,4]diazepin-10(2*H*)-ones were straightforwardly converted into 2-chloroethyl-substituted 1,4-benzodiazepine derivatives.

## 1. Introduction

**Figure 1:**
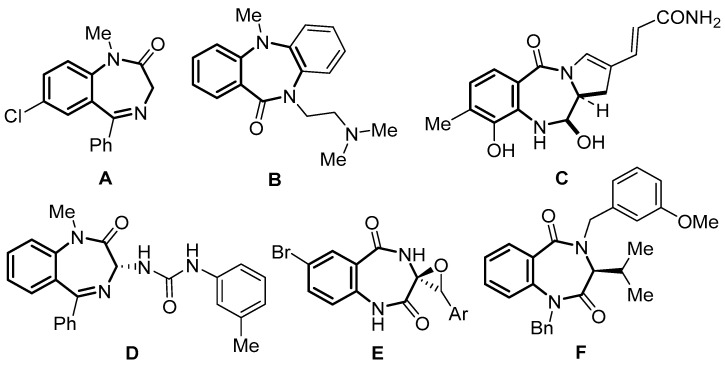
Representative 1,4-benzodiazepines with important biological activities.

1,4-Benzodiazepine is a privileged scaffold widely existing in natural products and biologically active compounds [1,2,3]. 1,4-Benzodiazepine derivatives are of significant interest in medicinal and pharmaceutical research because they exhibit a diverse range of biological activities, and some of them have been used in the clinical treatment of diseases [4]. For instance, diazepam **A** is the core structure of anxiolytics [5], anticonvulsants and hypnotics [6,7], and it is also an important fragment of some potential antiarrhythmics [8] and anti-HIV agents (Figure 1) [9]. Dibenzepin **B**, on the other hand, is a widely prescribed antidepressant drug [10], while compound **D** is used for the treatment of peptic ulcer disease [11]. In addition, some other 1,4-benzodiazepines also show antitumor (anthramycin **C**) [12,13], anticholinesterase (compound **E**) [14] and endothelin receptor antagonism activity (compound **F**) [15].

**Figure 2:**
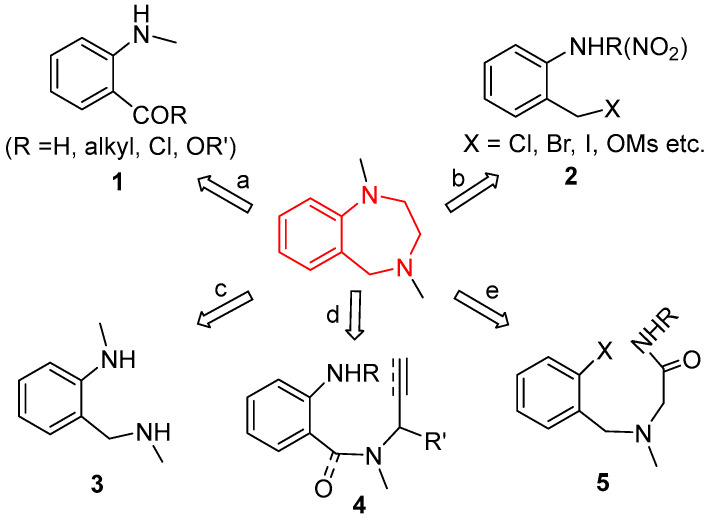
Synthetic strategies for the construction of 1,4-benzodiazepine derivatives.

As a consequence, the synthesis of 1,4-benzodiazepines has been attracting much attention from organic and medicinal chemists, and a variety of synthetic approaches to 1,4-benzodiazepine skeletons have been developed. The documented methods can be generally classified into the following five protocols [16]. As summarized in Figure 2, the most frequently used methods are the reaction of 2-aminobenzaldehyde, 2-aminophenone or 2-aminobenzoic acid and their derivatives **1** with α-amino acid (path a) [17,18,19,20,21,22,23], the cyclocondensation of 2-amino- or 2-nitrobenzyl halogen or alcohol **2** with amino acid derivatives (path b) [24,25,26,27] and the reaction between diamines **3** and bis-electrophiles (path c) [28,29,30,31]. The Pd-catalyzed C–N coupling reaction of *N*-allyl-2-aminobenzyl-/benzoylamines **4** [32,33,34,35] and the amidation of aromatic compounds **5** [36,37,38] provide other useful routes to the 1,4-benzodiazepine structure (paths d and e). However, most of the existing methods can only construct 1,4-benzodiazepines without a functional group at the 2- and 3-positions. To further search for 1,4-benzodiazepine molecules with potential biological activity for drug discovery, it is highly desirable and imperative to develop new methods for the facile construction of novel and functionalized 1,4-benzodiazepine derivatives.

**Scheme 1:**
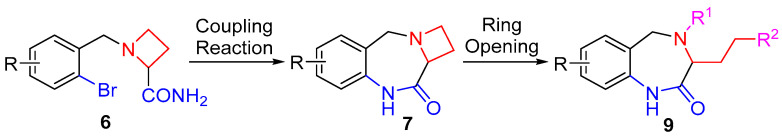
The rational design for the synthesis of functionalized 1,4-benzodiazepine derivatives.

Inspired by Wang’s previous work [39], we envisaged that the intramolecular cross-coupling reaction of 1-(2-bromobenzyl)azetidine-2-carboxamides **6** would provide a general method for the synthesis of novel azetidine-fused 1,4-diazepine derivatives **7** (Scheme 1). Moreover, the resulting products would be an invaluable platform for the preparation of functionalized 1,4-benzodiazepine derivatives **9** based on the opening of the azetidine ring by nucleophiles. We report herein the facile construction of 1,4,9,10a-tetrahydroazeto[1,2-*a*]benzo[e][1,4]diazepin-10(2*H*)-ones **7** by means of the CuI/*N*,*N*-dimethylglycine-catalyzed intramolecular C–N bond formation reaction of 1-(2-bromobenzyl)azetidine-2-carboxamides **6**. The efficient transformations of **7** into the diverse functionalized 1,4-benzodiazepine derivatives **9** through either *N*-methylation and the azetidine ring-opening reaction cascade or a straightforward reaction with methyl chloroformate are also presented. To the best of our knowledge, there is no precedent for the construction of azetidine-fused 1,4-diazepine derivatives and their application in the synthesis of 3-functionalized 1,4-benzodiazepine compounds.

## 2. Results and Discussion

**Scheme 2:**
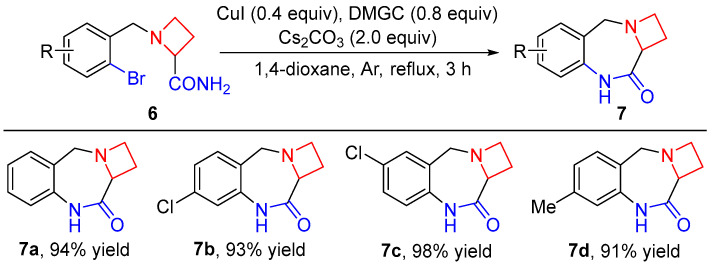
The intramolecular C–N cross-coupling reaction catalyzed by CuI/*N*,*N*-dimethylglycine.

We commenced our study with the examination of the intramolecular C–N bond coupling reaction of azetidine-2-carboxamides **6**. The substrates **6** were prepared according to a known procedure in the literature [40,41]. As we expected, catalyzed by CuI/*N*,*N*-dimethylglycine (DMGC), azetidine-2-carboxamides **6a**–**d** underwent the cross-coupling reaction smoothly to form the C–N bond efficiently under basic conditions. Thus, after refluxing in 1,4-dioxane for 3 h, fused heterocyclic ring products, 1,4,9,10*a*-tetrahydroazeto[1,2-*a*]benzo[*e*][1,4]diazepin-10(2*H*)-ones (**7**), were obtained in 91–98% yields (Scheme 2). It is worth noting that the aryl–Cl bond remained intact in the copper-catalyzed synthesis, remaining a useful handle for further chemical manipulations [42,43].

**Scheme 3:**
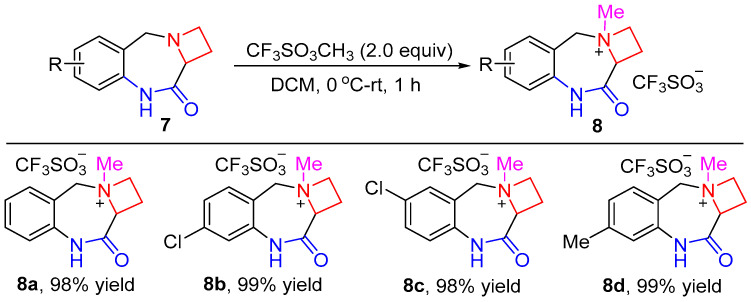
The methylation of 1,4,9,10a-tetrahydroazeto[1,2-a]benzo[e][1,4]-diazepin-10(2*H*)-ones.

The resulting precursors **7** represent a unique type of 1,4-benzodiazepine compounds that contain a fused four-membered *N*-heterocyclic ring. Owing to the relatively high ring strain, azetidine is prone to the ring-opening reaction [44,45,46,47]. The selective opening of the azetidine ring of the precursors **7** would form 1,3,4,5-tetrahydro-2*H*-benzo[*e*][1,4]diazepin-2-ones or 1,3,4,5,6,7-hexahydro-2H-benzo[*b*][1,5]diazonin-2-ones. Our interest in the derivatization of 1,4-benzodiazepine compounds led us to explore the reactivity of compounds **7**. However, the fused-ring precursors **7** were resistant to the ring-opening reaction, and their direct reactions with nucleophiles did not lead to any products. To facilitate the ring-opening reaction of azetidine, azetidines **7** were converted into the corresponding quaternary ammonium salts. After screening a variety of alkylating reagents, including methyl iodide, benzyl bromide, etc., we discovered that methyl triflate was a highly reactive reagent for the selective methylation reaction (Scheme 3). Upon treatment with two equivalents of methyl triflate under mild reaction conditions, compounds **7** were transformed almost quantitatively into ammonium triflates **8**. No methylation on the amide moiety was observed because of the higher basicity of azetidine.

With ammonium salts in hand, we studied the ring-opening reaction of **8** with different nucleophiles. With compound **8d** as the model substrate (Table 1), the opening of the azetidinium ring employing NaN_3_ as a nucleophile was surveyed. Pleasingly, the reaction of **8d** with NaN_3_ in DMF at room temperature proceeded smoothly to afford 3-(2-azidoethyl)-4,8-dimethyl-1,3,4,5-tetrahydro-2*H*-benzo[*e*][1,4]diazepin-2-one (**9da**) (Nu = N_3_) in a 91% yield (entry 1, Table 1). As expected, replacement of the reaction medium from polar DMF to THF or DCM resulted in a slight decrease of both the reaction rate and the chemical yield (entries 2–3, Table 1). When KCN was used as the nucleophilic reagent, the corresponding ring-opening product **9db** (Nu = CN) was obtained in a moderate yield under the same conditions (entry 4, Table 1), but the chemical yield could be improved to 78% by elongating the reaction time to 24 h (entry 5, Table 1). Sodium thiophenolate (PhSNa) acted as an excellent nucleophile to open the azetidinium ring of **8d** to furnish the sulfide-bearing product **9dc** in a nearly quantitative yield (entry 6, Table 1). It should be noted, however, that sodium phenoxide (PhONa) did not react with **8da**, most probably due to its lower nucleophilicity in comparison with PhSNa (entries 7–10, Table 1). It is interesting to address the fact that, for the ring-opening reaction of **8**, a nucleophilic attack on the azetidinium moiety always occurred at C_3_ rather than the C_1_ position. In other words, the reaction gave seven-membered heterocyclic products **8** instead of nine-membered ones, which has been confirmed unambiguously through the X-ray single crystal of **9aa** (see Supporting Information). The superb regioselectivity was most likely attributable to the steric effect since methylene was more accessible by an attacking nucleophilic reagent than methine between the ammonium and carbonyl moieties.

**Scheme 4:**
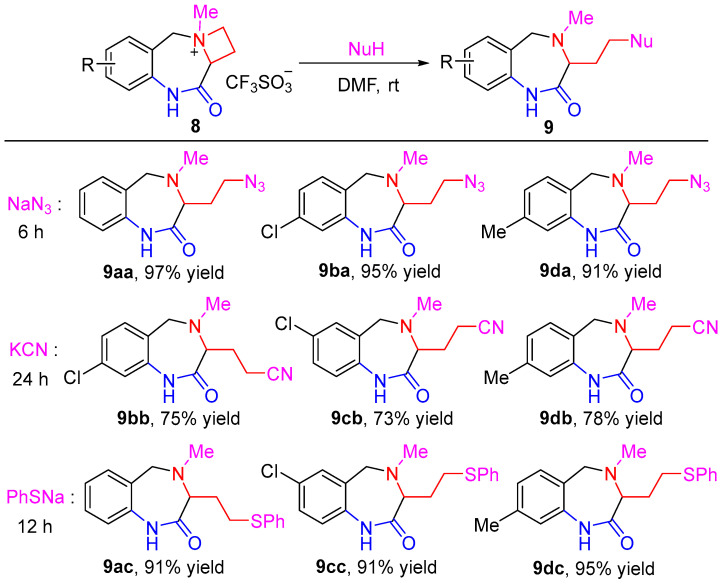
The ring-opening reaction of trifluoromethanesulfonyl salts **8** with different nucleophiles. Reaction conditions: compound **8** (1.0 mmol, 1.0 equiv), nucleophile (2.0 mmol, 2.0 equiv) and DMF (5.0 mL) were added, and then the mixture was stirred at room temperature. The isolated yields are given based on compound **8**.

The synthesis of functionalized 1,4-benzodiazepine derivatives by means of the selective azitidinium ring-opening reaction was readily extended to other substrates. As demonstrated in Scheme 4, all the substrates **8a**–**d** reacted well with NaN_3_, KCN and PhSNa, affording a range of diverse functionalized 1,4-benzodiazepines derivatives in moderate to excellent yields within 6–24 h (Scheme 4). It was clear that NaN_3_ was the most powerful reactant, and the reaction proceeded most rapidly and efficiently to give excellent yields of products **9aa**–**ba** and **9da**. The sulfide-containing products **9ac** and **9cc**–**dc** were also obtained in very high yields using the reaction of **8** with PhSNa, albeit a slightly longer reaction period was required. In the case of KCN, the ring-opening reaction took 24 h to complete the formation of **9bb**–**db** as the sole products. Due to the strong absorption on silica gel, they were isolated in a yield of 73–78% after silica gel column chromatography. The nature of the substituent on the benzene ring had virtually no influence on the outcomes of the ring-opening reaction, rendering the method applicable to the preparation of various substituted 1,3,4,5-tetrahydro-2*H*-benzo[*e*][1,4]diazepin-2-one products.

**Scheme 5:**
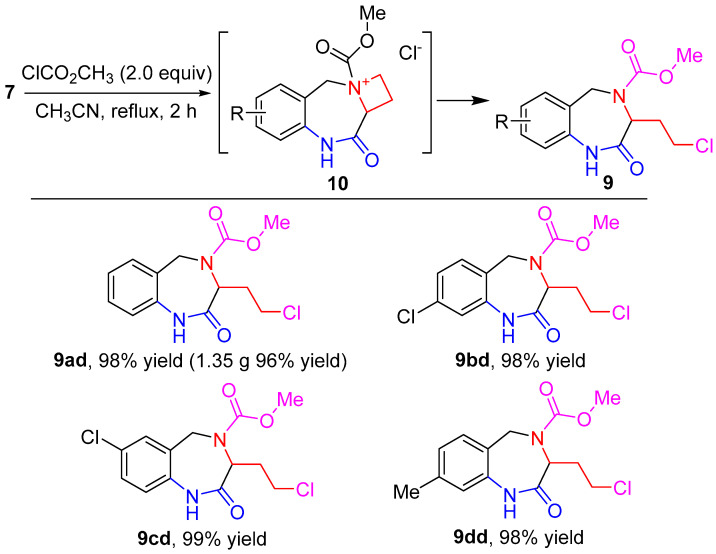
The ring-opening reaction of 1,4,9,10a-tetrahydroazeto[1,2-a]benzo[e][1,4]diazepin-10(2*H*)-ones **7** with methyl chloroformate. Reaction conditions: compound **7** (1.0 mmol, 1.0 equiv), methyl chloroformate (2.0 mmol, 2.0 equiv) and CH_3_CN (10 mL) were refluxed for 2 h. The isolated yields are given based on compound **7**.

Encouraged by the facile and selective reaction of the azetidinium ring in **8**, we turned our attention to the reaction of **7** with acyl chlorides. It was envisaged that acylation on the nitrogen atom in azetidine **7** would further enhance the ring-opening reactivity of the resulting *N*-methoxycarbonyl azetidinium derivative [48,49,50]. To our delight, compounds **7a**–**d** and methyl chloroformate in acetonitrile with reflux gave rise to the formation of 2-chloroethyl-substituted 1,3,4,5-tetrahydro-2*H*-benzo[*e*][1,4]diazepin-2-one products. The complete and straightforward conversion of compounds **7** into products **9** indicated that the reaction proceeded efficiently the *N*-methoxycarbonylation, followed by the ring opening of the in situ generated *N*-methoxycarbonyl azetidinium derivatives by chloride anion (Scheme 5). The synthesis could be readily scaled up, as exemplified by the gram-scale synthesis of **9ad** (Scheme 5).

## 3. Conclusions

In summary, we have provided a facile and efficient protocol to synthesize diverse functionalized 1,4-benzodiazepine derivatives using intramolecular C–N bond coupling and the successive ring-opening of azetidines. The easy availability of all the starting materials and the mild reaction conditions render the method versatile and useful for the preparation of novel and unique 1,4-benzodiazepine derivatives, which are in high demand for drug discovery and development.

## 4. Materials and Methods

### 4.1. General Information

All the chemicals were dried or purified according to standard procedures prior to use. The flash column chromatography was performed on silica gel (100–200). The reactions were monitored using pre-coated, glass-backed silica gel plates and visualized by means of UV irradiation (254 nm) or KMnO_4_. The ^1^H NMR and ^13^C NMR spectra were recorded on a Bruker AV500 spectrometer at ambient temperature. The chemical shifts are reported in ppm with either tetramethylsilane or the residual solvent resonance used as an internal standard. The high-resolution mass spectra (HRMS) were measured on a quadrupole tome-of-flight mass spectrometer (Q-TOF-MS) using electrospray ionization (ESI) as an ionization method. Crystallographic data were collected on a Rigaku XtaLAB Synergy (Cu) X-ray single crystal diffractometer. All the yields reported are the isolated yields.

### 4.2. General Procedure for the Synthesis of Compounds ***6a***–***d***

*Step 1*: To a flask (250 mL) equipped with a magnetic stirrer were added the corresponding 2-((2-bromobenzyl)(2-chloroethyl)amino)acetonitrile (50 mmol), potassium *tert*-butoxide (11.2 g, 100 mmol) and dry THF (100 mL). The reaction mixture was stirred at room temperature until the substrate disappeared and then saturated NH_4_Cl aqueous solution (50 mL) was added to quench the reaction. The mixture was concentrated in vacuo to remove the THF and the residue was extracted with ethyl acetate (4 × 50 mL) and washed with brine (1 × 50 mL). The organic layer was dried over anhydrous Na_2_SO_4_ and concentrated under a vacuum to give the crude product 1-(2-bromobenzyl)azetidine-2-carbonitrile, which was used immediately without further purification.

*Step 2*: Under slight heating and stirring, the product from the previous step was dissolved in *tert*-butanol (50 mL), and then anhydrous KOH (168 mg, 30 mmol) was added in portions. The resulting mixture was stirred at reflux until the substrate disappeared and water (20 mL) was added to quench the reaction. The mixture was extracted with ethyl acetate (3 × 50 mL), and washed with brine (2 × 50 mL). The organic layer was dried over anhydrous Na_2_SO_4_, and concentrated under a vacuum. The residue was chromatographed on a silica gel column eluted with a mixture of petroleum ether and ethyl acetate (3:1) to give pure product **6**.

**1-(2-Bromobenzyl)azetidine-2-carboxamide (6a)**. Light yellow solid (41% yield, 2 steps). m.p. 140–141 °C; IR (KBr) ν 3413.3, 1675.0 cm^−1^; ^1^H NMR (400 MHz, chloroform-*d*) δ 7.56 (d, *J* = 8.1 Hz, 1H), 7.32–7.25 (m, 2H), 7.23 (br s, 1H), 7.18–7.10 (m, 1H), 6.06 (br s, 1H), 3.82 (d, *J* = 12.9 Hz, 1H), 3.74 (t, *J* = 8.6 Hz, 1H), 3.64 (d, *J* = 13.0 Hz, 1H), 3.37–3.29 (m, 1H), 3.04 (q, *J* = 8.4 Hz, 1H), 2.47–2.39 (m, 1H), 2.24–2.12 (m, 1H); ^13^C NMR (101 MHz, chloroform-*d*) δ 176.2, 136.5, 133.1, 130.8, 129.2, 127.6, 124.5, 66.0, 62.0, 50.9, 23.1; HRMS (ESI) m/z [M + H]^+^ calcd. for C_11_H_14_BrN_2_O 269.0284, 271.0264; found 269.0291, 271.0270.

**1-(2-Bromo-4-chlorobenzyl)azetidine-2-carboxamide (6b)**. Light yellow solid (36% yield, 2 steps). m.p. 146–147 °C; IR (KBr) ν 3398.2, 1674.9 cm^−1^; ^1^H NMR (400 MHz, chloroform-*d*) δ 7.60 (d, *J* = 1.6 Hz, 1H), 7.33–7.28 (m, 2H), 7.20 (br s, 1H), 5.83 (br s, 1H), 3.87–3.73 (m, 2H), 3.66 (d, *J* = 13.2 Hz, 1H), 3.42–3.32 (m, 1H), 3.05 (q, *J* = 8.3 Hz, 1H), 2.53–2.42 (m, 1H), 2.30–2.17 (m, 1H); ^13^C NMR (101 MHz, chloroform-*d*) δ 175.8, 135.1, 134.2, 132.8, 131.4, 127.9, 124.8, 66.0, 61.4, 51.0, 23.2; HRMS (ESI) m/z [M + H]^+^ calcd. for C_11_H_13_BrClN_2_O 302.9894, 304.9874; found 302.9889, 304.9869.

**1-(2-Bromo-5-chlorobenzyl)azetidine-2-carboxamide (6c)**. Yellow solid (21% yield, 2 steps). m.p. 158–160 °C; IR (KBr) ν 3369.2, 1676.5 cm^−1^; ^1^H NMR (400 MHz, chloroform-*d*) δ 7.48 (d, *J* = 8.5 Hz, 1H), 7.31 (d, *J* = 2.5 Hz, 1H), 7.18 (br s, 1H), 7.13 (dd, *J* = 8.5, 2.6 Hz, 1H), 5.76 (br s, 1H), 3.83–3.72 (m, 2H), 3.60 (d, *J* = 13.7 Hz, 1H), 3.42–3.35 (m, 1H), 3.07–2.98 (m, 1H), 2.51–2.41 (m, 1H), 2.27–2.16 (m, 1H); ^13^C NMR (101 MHz, chloroform-*d*) δ 175.7, 138.4, 134.1, 133.7, 130.3, 129.2, 122.1, 66.1, 61.7, 51.3, 23.2; HRMS (ESI) m/z [M + H]^+^ calcd. for C_11_H_13_BrClN_2_O 302.9894, 304.9874; found 302.9888, 304.9868.

**1-(2-Bromo-4-methylbenzyl)azetidine-2-carboxamide (6d).** Light yellow solid (22% yield, 2 steps). m.p. 135–137 °C; IR (KBr) ν 3414.2, 1667.5 cm^−1^; ^1^H NMR (400 MHz, chloroform-*d*) δ 7.36 (s, 1H), 7.19 (br s, 1H), 7.14 (d, *J* = 7.7 Hz, 1H), 7.07–7.01 (m, 1H), 6.20 (br s, 1H), 3.75 (d, *J* = 12.8 Hz, 1H), 3.68 (t, *J* = 8.6 Hz, 1H), 3.57 (d, *J* = 12.8 Hz, 1H), 3.32–3.24 (m, 1H), 2.99 (q, *J* = 8.4 Hz, 1H), 2.45–2.34 (m, 1H), 2.28 (s, 3H), 2.20–2.08 (m, 1H); ^13^C NMR (101 MHz, chloroform-*d*) δ 176.3, 139.3, 133.5, 133.3, 130.6, 128.3, 124.3, 65.9, 61.6, 50.7, 23.0, 20.7; HRMS (ESI) m/z [M + H]^+^ calcd. for C_12_H_16_BrN_2_O 283.0441, 285.0420; found 283.0448, 285.0427.

### 4.3. General Procedure for the Synthesis of Compounds ***7a***–***d***

Based on previous work [38], to a flask (50 mL) equipped with a magnetic stirrer were added compound **6** (1.0 mmol), CuI (0.4 mmol, 76 mg), DMGC (0.8 mmol, 112 mg), Cs_2_CO_3_ (2 mmol, 650 mg), and anhydrous 1,4-dioxane (34 mL) under argon protection. The reaction mixture was stirred at reflux for about 3 h until the substrate disappeared. After the reaction, ethyl acetate (100 mL) was added to dilute the mixture before filtration. The solvents were removed in vacuo and the residue was purified by flash column chromatography (PE:EA = 1:1) to afford products **7**.

**1,4,9,10a-Tetrahydroazeto[1,2-a]benzo[e][1,4]diazepin-10(2*H*)-one (7a).** White solid (94% yield, 176.8 mg). m.p. 184–186 °C; IR (KBr) ν 3187.6, 1657.2 cm^−1^; ^1^H NMR (400 MHz, chloroform-*d*) δ 8.20 (br s, 1H), 7.29 (d, *J* = 7.4 Hz, 2H), 7.15 (t, *J* = 7.5 Hz, 1H), 7.00 (d, *J* = 7.7 Hz, 1H), 4.07 (dd, *J* = 7.8, 2.4 Hz, 1H), 3.79–3.66 (m, 2H), 3.56–3.41 (m, 2H), 2.69–2.60 (m, 1H), 2.49–2.37 (m, 1H); ^13^C NMR (101 MHz, chloroform-*d*) δ 173.0, 136.9, 130.4, 130.0, 129.0, 125.9, 121.7, 64.5, 56.7, 54.9, 19.6; HRMS (ESI) m/z [M + H]^+^ calcd. for C_11_H_13_N_2_O 189.1022; found 189.1028.

**7-Chloro-1,4,9,10a-tetrahydroazeto[1,2-a]benzo[e][1,4]diazepin-10(2*H*)-one (7b).** White solid (93% yield, 206.5 mg). m.p. 192–192 °C; IR (KBr) ν 3177.6, 1624.2 cm^−1^; ^1^H NMR (400 MHz, chloroform-*d*) δ 8.26 (br s, 1H), 7.22 (d, *J* = 8.1 Hz, 1H), 7.13 (dd, *J* = 8.1, 2.0 Hz, 1H), 7.03 (d, *J* = 1.9 Hz, 1H), 4.07 (dd, *J* = 7.7, 2.4 Hz, 1H), 3.74–3.64 (m, 2H), 3.57–3.42 (m, 2H), 2.68–2.61 (m, 1H), 2.48–2.39 (m, 1H); ^13^C NMR (101 MHz, chloroform-*d*) δ 173.4, 138.3, 134.3, 131.1, 128.7, 125.8, 121.8, 64.5, 56.2, 54.9, 19.6; HRMS (ESI) m/z [M + H]^+^ calcd. for C_11_H_12_ClN_2_O 223.0633; found 223.0640.

**6-Chloro-1,4,9,10a-tetrahydroazeto[1,2-a]benzo[e][1,4]diazepin-10(2H)-one (7c).** White solid (98% yield, 217.6 mg). m.p. 184–185 °C; IR (KBr) ν 3169.7, 1677.2 cm^−1^; ^1^H NMR (400 MHz, chloroform-*d*) δ 8.42 (br s, 1H), 7.29–7.23 (m, 2H), 6.98–6.93 (m, 1H), 4.05 (dd, *J* = 7.8, 2.7 Hz, 1H), 3.70 (d, *J* = 11.0 Hz, 1H), 3.62 (d, *J* = 11.0 Hz, 1H), 3.55–3.41 (m, 2H), 2.66–2.60 (m, 1H), 2.48–2.38 (m, 1H); ^13^C NMR (101 MHz, chloroform-*d*) δ 173.0, 135.5, 131.9, 131.0, 130.0, 128.9, 123.0, 64.5, 56.3, 54.9, 19.7; HRMS (ESI) m/z [M + H]^+^ calcd. for C_11_H_12_ClN_2_O 223.0633; found 223.0641.

**7-Methyl-1,4,9,10a-tetrahydroazeto[1,2-a]benzo[e][1,4]diazepin-10(2*H*)-one (7d).** White solid (91% yield, 183.9 mg). m.p. 211–212 °C; IR (KBr) ν 3188.6, 1662.9 cm^−1^; ^1^H NMR (400 MHz, chloroform-*d*) δ 8.59 (br s, 1H), 7.15 (d, *J* = 7.7 Hz, 1H), 6.95 (d, *J* = 7.1 Hz, 1H), 6.81 (s, 1H), 4.05 (dd, *J* = 7.8, 2.4 Hz, 1H), 3.69 (d, *J* = 10.9 Hz, 1H), 3.64 (d, *J* = 10.9 Hz, 1H), 3.56–3.40 (m, 2H), 2.67–2.61 (m, 1H), 2.48–2.37 (m, 1H), 2.32 (s, 3H); ^13^C NMR (101 MHz, chloroform-*d*) δ 173.3, 138.9, 136.8, 129.8, 127.3, 126.5, 122.2, 64.4, 56.4, 54.8, 21.2, 19.6; HRMS (ESI) m/z [M + H]^+^ calcd. for C_12_H_13_N_2_O 203.1179; found 203.1172.

### 4.4. General Procedure for the Synthesis of Compounds ***8a***–***d***

To a flask (10 mL) equipped with a magnetic stirrer were added compound **7** (1.0 mmol) and anhydrous DCM (5 mL). After cooling to 0 °C, methyl triflate (0.22 mL, 2.0 mmol) was slowly added, and then the reaction mixture was stirred at room temperature for 1 h. The solvents were removed in vacuo and the residue was washed several times with a small amount of dry ether to afford products **8**.

**3-Methyl-10-oxo-2,3,4,9,10,10a-hexahydro-1*H*-azeto[1,2-a]benzo[e][1,4]diazepin-3-ium triflate (8a).** White solid (98% yield, 345.0 mg). m.p. 167–167 °C; IR (KBr) ν 3229.8, 1692.5 cm^−1^; ^1^H NMR (400 MHz, acetonitrile-*d*_3_) δ 9.01 (br s, 1H), 7.59–7.52 (m, 1H), 7.45 (d, *J* = 7.3 Hz, 1H), 7.31 (t, *J* = 7.5 Hz, 1H), 7.25 (d, *J* = 8.1 Hz, 1H), 4.91 (d, *J* = 13.3 Hz, 1H), 4.82 (dd, *J* = 9.3, 6.1 Hz, 1H), 4.56 (q, *J* = 10.0 Hz, 1H), 4.32–4.23 (m, 2H), 3.13–2.90 (m, 5H); ^13^C NMR (101 MHz, acetonitrile-*d*_3_) δ 165.7, 137.1, 133.1, 132.8, 126.8, 123.3, 122.3, 121.9 (q, *J* = 322.2 Hz, 1C), 74.3, 65.6, 62.9, 50.1, 18.8; HRMS (ESI) m/z [M-OTf]^+^ calcd. for C_12_H_15_N_2_O 203.1179; found: 203.1185.

**7-Chloro-3-methyl-10-oxo-2,3,4,9,10,10a-hexahydro-1*H*-azeto[1,2-a]benzo[e][1,4]diazepin-3-ium triflate (8b).** White solid (99% yield, 382.2 mg). m.p. 206–207 °C; IR (KBr) ν 3209.5, 1678.7 cm^−1^; ^1^H NMR (400 MHz, acetonitrile-*d*_3_) δ 9.01 (br s, 1H), 7.43 (d, *J* = 8.2 Hz, 1H), 7.36–7.24 (m, 2H), 4.92–4.79 (m, 2H), 4.55 (q, *J* = 9.9 Hz, 1H), 4.34–4.21 (m, 2H), 3.05 (s, 3H), 3.03–2.92 (m, 2H); ^13^C NMR (101 MHz, acetonitrile-*d*_3_) δ 165.6, 138.7, 137.8, 134.6, 126.8, 123.1, 122.0 (q, *J* = 323.2 Hz, 1C), 121.1, 74.4, 65.8, 62.4, 50.2, 18.8; HRMS (ESI) m/z [M-OTf]^+^ calcd. for C_12_H_14_ClN_2_O 237.0789; found: 237.0794.

**6-Chloro-3-methyl-10-oxo-2,3,4,9,10,10a-hexahydro-1*H*-azeto[1,2-a]benzo[e][1,4]diazepin-3-ium triflate (8c).** White solid (98% yield, 378.3 mg). m.p. 215–216 °C; IR (KBr) ν 3200.3, 1678.1 cm^−1^; ^1^H NMR (400 MHz, acetonitrile-*d*_3_) δ 9.05 (br s, 1H), 7.60–7.46 (m, 2H), 7.24 (d, *J* = 8.6 Hz, 1H), 4.94–4.79 (m, 2H), 4.57 (q, *J* = 9.9 Hz, 1H), 4.33–4.23 (m, 2H), 3.09 (s, 3H), 3.05–2.92 (m, 2H); ^13^C NMR (101 MHz, acetonitrile-*d*_3_) δ 165.5, 136.2, 132.64, 132.60, 131.3, 124.9, 124.0, 121.9 (q, *J* = 322.2 Hz, 1C), 74.4, 65.9, 62.3, 50.3, 18.8; HRMS (ESI) m/z [M-OTf]^+^ calcd. for C_12_H_14_ClN_2_O 237.0789; found: 237.0796.

**3,7-Dimethyl-10-oxo-2,3,4,9,10,10a-hexahydro-1*H*-azeto[1,2-a]benzo[e][1,4]diazepin-3-ium triflate (8d).** White solid (99% yield, 362.4 mg). m.p. 192–194 °C; IR (KBr) ν 3224.6, 1676.3 cm^−1^; ^1^H NMR (400 MHz, deuterium oxide) δ 7.40 (d, *J* = 7.8 Hz, 1H), 7.24 (d, *J* = 7.8 Hz, 1H), 7.13 (s, 1H), 5.09 (dd, *J* = 11.2, 6.3 Hz, 2H), 4.66 (q, *J* = 10.4 Hz, 1H), 4.46–4.38 (m, 1H), 4.38–4.32 (m, 1H), 3.23–2.99 (m, 5H), 2.41 (s, 3H); ^13^C NMR (101 MHz, acetonitrile-*d*_3_) δ 165.7, 143.6, 137.0, 132.9, 127.6, 123.5, 122.0 (q, *J* = 322.2 Hz, 1C), 119.4, 74.2, 65.4, 62.8, 50.0, 21.2, 18.7; HRMS (ESI) m/z [M-OTf]^+^ calcd. for C_13_H_17_N_2_O 217.1335; found: 217.1341.

### 4.5. General Procedure for the Ring-Opening Reaction of Compounds ***8***

To a flask (10 mL) equipped with a magnetic stirrer were added compound **8** (1.0 mmol), different nucleophiles (2 mmol) (NaN_3_, KCN and PhSNa) and dry DMF (5 mL). After the addition, the reaction mixture was stirred at room temperature until the substrate disappeared (for NaN_3_, about 6 h; for KCN, about 24 h; for PhSNa, about 12 h), and then water (5 mL) was added to quench the reaction. The mixture was extracted with ethyl acetate (3 × 10 mL), and the combined organic layers were washed with brine (1 × 20 mL) and dried over anhydrous Na_2_SO_4_. The solvents were removed in vacuo and the residue was purified by flash column chromatography (PE:EA = 1:1) to afford products **9**.

**3-(2-Azidoethyl)-4-methyl-1,3,4,5-tetrahydro-2*H*-benzo[e][1,4]diazepin-2-one (9aa).** White solid (97% yield, 237.8 mg). m.p. 106–107 °C; IR (KBr) ν 3180.8, 2104.3, 1680.3 cm^−1^; ^1^H NMR (400 MHz, chloroform-*d*) δ 8.33 (br s, 1H), 7.33–7.26 (m, 2H), 7.16 (t, *J* = 7.2 Hz, 1H), 6.99 (d, *J* = 7.7 Hz, 1H), 3.93–3.76 (m, 2H), 3.49–3.38 (m, 2H), 3.36–3.29 (m, 1H), 2.39 (s, 3H), 2.19–2.10 (m, 1H), 1.91–1.81 (m, 1H); ^13^C NMR (101 MHz, chloroform-*d*) δ 172.4, 137.3, 130.5, 129.0 (2C), 125.3, 120.7, 60.8, 58.1, 48.6, 39.5, 28.7; HRMS (ESI) m/z [M + H]^+^ calcd. for C_12_H_16_N_5_O 246.1349; found 246.1355.

**3-(2-Azidoethyl)-8-chloro-4-methyl-1,3,4,5-tetrahydro-2*H*-benzo[e][1,4]diazepin-2-one (9ba).** White solid (95% yield, 265.1 mg). m.p. 101–101 °C; IR (KBr) ν 3178.4, 2084.1, 1681.4 cm^−1^; ^1^H NMR (400 MHz, chloroform-*d*) δ 8.96 (br s, 1H), 7.17 (d, *J* = 8.1 Hz, 1H), 7.11 (dd, *J* = 8.1, 1.9 Hz, 1H), 7.04 (d, *J* = 1.8 Hz, 1H), 3.85 (d, *J* = 13.9 Hz, 1H), 3.75 (d, *J* = 13.9 Hz, 1H), 3.50–3.39 (m, 2H), 3.38–3.30 (m, 1H), 2.38 (s, 3H), 2.20–2.08 (m, 1H), 1.90–1.80 (m, 1H); ^13^C NMR (101 MHz, chloroform-*d*) δ 173.0, 138.6, 134.3, 131.4, 127.4, 125.2, 120.7, 61.0, 57.5, 48.5, 39.3, 28.6; HRMS (ESI) m/z [M + H]^+^ calcd. for C_12_H_15_ClN_5_O 280.0960; found 280.0966.

**3-(2-Azidoethyl)-4,8-dimethyl-1,3,4,5-tetrahydro-2*H*-benzo[e][1,4]diazepin-2-one (9da).** White solid (91% yield, 235.8 mg). m.p. 105–107 °C; IR (KBr) ν 3168.7, 2081.9, 1681.5 cm^−1^; ^1^H NMR (400 MHz, chloroform-*d*) δ 8.04 (br s, 1H), 7.15 (d, *J* = 7.6 Hz, 1H), 6.97 (d, *J* = 7.7 Hz, 1H), 6.79 (s, 1H), 3.84 (d, *J* = 13.6 Hz, 1H), 3.74 (d, *J* = 13.6 Hz, 1H), 3.47–3.37 (m, 2H), 3.35–3.26 (m, 1H), 2.38 (s, 3H), 2.35 (s, 3H), 2.19–2.08 (m, 1H), 1.90–1.78 (m, 1H); ^13^C NMR (101 MHz, chloroform-*d*) δ 172.1, 139.2, 137.2, 130.4, 126.2, 125.9, 121.2, 60.7, 57.8, 48.7, 39.5, 28.6, 21.2; HRMS (ESI) m/z [M + H]^+^ calcd. for C_13_H_18_N_5_O 260.1506; found 260.1512.

**3-(8-Chloro-4-methyl-2-oxo-2,3,4,5-tetrahydro-1*H*-benzo[e][1,4]diazepin-3-yl)propanenitrile (9bb).** White solid (75% yield, 197.3 mg). m.p. 147–148 °C; IR (KBr) ν 3193.8, 2237.7, 1696.3 cm^−1^; ^1^H NMR (400 MHz, chloroform-*d*) δ 8.69 (br s, 1H), 7.20–7.09 (m, 2H), 7.02 (s, 1H), 3.85 (s, 2H), 3.33 (t, *J* = 5.9 Hz, 1H), 2.56–2.46 (m, 2H), 2.34 (s, 3H), 2.24–2.14 (m, 1H), 2.02–1.92 (m, 1H); ^13^C NMR (101 MHz, chloroform-*d*) δ 172.5, 138.0, 134.4, 131.5, 127.0, 125.3, 120.8, 119.6, 62.9, 57.6, 39.2, 25.1, 14.0; HRMS (ESI) m/z [M + H]^+^ calcd. for C_13_H_15_ClN_3_O 264.0898; found 264.0905.

**3-(7-Chloro-4-methyl-2-oxo-2,3,4,5-tetrahydro-1*H*-benzo[e][1,4]diazepin-3-yl)propanenitrile (9cb).** White solid (73% yield, 192.1 mg). m.p. 170–171 °C; IR (KBr) ν 3305.8, 2246.7, 1666.0 cm^−1^; ^1^H NMR (400 MHz, chloroform-*d*) δ 8.41 (br s, 1H), 7.30–7.27 (m, 1H), 7.22 (d, *J* = 2.1 Hz, 1H), 6.94 (d, *J* = 8.4 Hz, 1H), 3.90 (d, *J* = 14.6 Hz, 1H), 3.84 (d, *J* = 14.6 Hz, 1H), 3.33 (t, *J* = 6.7 Hz, 1H), 2.57–2.47 (m, 2H), 2.36 (s, 3H), 2.23–2.14 (m, 1H), 2.06–1.92 (m, 1H); ^13^C NMR (101 MHz, chloroform-*d*) δ 172.3, 135.4, 130.4, 130.2, 130.1, 129.0, 122.0, 119.7, 63.1, 57.8, 39.2, 25.2, 13.9; HRMS (ESI) m/z [M + H]^+^ calcd. for C_13_H_15_ClN_3_O 264.0898; found 264.0906.

**3-(4,8-Dimethyl-2-oxo-2,3,4,5-tetrahydro-1*H*-benzo[e][1,4]diazepin-3-yl)propanenitrile (9db).** White solid (78% yield, 189.6 mg). m.p. 161–162 °C; IR (KBr) ν 3173.7, 2245.7, 1681.1 cm^−1^; ^1^H NMR (400 MHz, chloroform-*d*) δ 8.66 (br s, 1H), 7.11 (d, *J* = 7.5 Hz, 1H), 6.96 (d, *J* = 7.5 Hz, 1H), 6.81 (s, 1H), 3.95–3.74 (m, 2H), 3.27 (t, *J* = 6.5 Hz, 1H), 2.54–2.44 (m, 2H), 2.34 (s, 6H), 2.21–2.11 (m, 1H), 2.01–1.90 (m, 1H); ^13^C NMR (101 MHz, chloroform-*d*) δ 172.2, 139.1, 136.8, 130.2, 126.1, 125.5, 121.3, 119.8, 62.7, 57.7, 39.5, 25.2, 21.2, 13.9; HRMS (ESI) m/z [M + H]^+^ calcd. for C_14_H_18_N_3_O 244.1444; found 244.1438.

**4-Methyl-3-(2-(phenylthio)ethyl)-1,3,4,5-tetrahydro-2*H*-benzo[e][1,4]diazepin-2-one (9ac).** White solid (91% yield, 284.1 mg). m.p. 107–108 °C; IR (KBr) ν 3305.8, 1670.1 cm^−1^; ^1^H NMR (400 MHz, chloroform-*d*) δ 9.02 (br s, 1H), 7.31–7.19 (m, 5H), 7.16–7.08 (m, 2H), 7.00 (dd, *J* = 7.8, 2.7 Hz, 1H), 3.87–3.72 (m, 2H), 3.46 (td, *J* = 7.0, 3.5 Hz, 1H), 3.04–2.89 (m, 2H), 2.36 (d, *J* = 3.5 Hz, 3H), 2.25–2.15 (m, 1H), 1.95–1.83 (m, 1H); ^13^C NMR (101 MHz, chloroform-*d*) δ 173.0, 137.5, 136.4, 130.3, 129.1, 129.0 (2C), 128.8, 125.9, 125.1, 120.7, 62.6, 58.0, 39.6, 30.4, 29.1; HRMS (ESI) m/z [M + H]^+^ calcd. for C_18_H_21_N_2_OS 313.1369; found 313.1374.

**7-Chloro-4-methyl-3-(2-(phenylthio)ethyl)-1,3,4,5-tetrahydro-2*H*-benzo[e][1,4]diazepin-2-one (9cc).** White solid (91% yield, 314.9 mg). m.p. 129–130 °C; IR (KBr) ν 3316.9, 1659.2 cm^−1^; ^1^H NMR (400 MHz, chloroform-*d*) δ 8.34 (br s, 1H), 7.30 (d, *J* = 7.5 Hz, 2H), 7.25–7.22 (m, 4H), 7.15 (t, *J* = 7.2 Hz, 1H), 6.89 (d, *J* = 8.3 Hz, 1H), 3.80 (s, 2H), 3.47 (t, *J* = 6.9 Hz, 1H), 3.07–2.90 (m, 2H), 2.35 (s, 3H), 2.23–2.14 (m, 1H), 1.95–1.86 (m, 1H); ^13^C NMR (101 MHz, chloroform-*d*) δ 172.8, 136.3, 135.8, 130.7, 130.2 (2C), 129.3, 129.0, 128.8, 126.1, 121.9, 62.8, 57.6, 39.5, 30.4, 29.1; HRMS (ESI) m/z [M + H]^+^ calcd. for C_18_H_20_ClN_2_OS 347.0979; found 347.0972.

**4,8-Dimethyl-3-(2-(phenylthio)ethyl)-1,3,4,5-tetrahydro-2*H*-benzo[e][1,4]diazepin-2-one (9dc).** White solid (95% yield, 309.8 mg). m.p. 137–138 °C; IR (KBr) ν 3295.9, 1663.1 cm^−1^; ^1^H NMR (400 MHz, chloroform-*d*) δ 8.37 (br s, 1H), 7.30–7.27 (m, 2H), 7.24–7.17 (m, 2H), 7.15–7.09 (m, 2H), 6.95 (d, *J* = 7.8 Hz, 1H), 6.77 (s, 1H), 3.80 (d, *J* = 13.6 Hz, 1H), 3.72 (d, *J* = 13.5 Hz, 1H), 3.44 (t, *J* = 6.9 Hz, 1H), 3.04–2.87 (m, 2H), 2.35 (s, 3H), 2.34 (s, 3H), 2.25–2.13 (m, 1H), 1.94–1.82 (m, 1H); ^13^C NMR (101 MHz, chloroform-*d*) δ 172.7, 138.9, 137.3, 136.5, 130.2, 129.1, 129.0, 126.1, 126.0, 125.9, 121.2, 62.5, 57.7, 39.7, 30.4, 29.1, 21.2; HRMS (ESI) m/z [M + H]^+^ calcd. for C_19_H_23_N_2_OS 327.1526; found 327.1533.

### 4.6. General Procedure for the Ring-Opening Reaction of Compounds ***7***

To a flask (25 mL) equipped with a magnetic stirrer were added compound **7** (1.0 mmol) and anhydrous CH_3_CN (10 mL).Then, methyl chloroformate (0.19 mL, 2.0 mmol) was added dropwise. After the addition, the reaction mixture was stirred at reflux for about 2 h until the substrate disappeared, and then saturated NaHCO_3_ aqueous solution (10 mL) was added to quench the reaction. The mixture was extracted with ethyl acetate (3 × 20 mL), and the combined organic layers were washed with brine (1 × 30 mL) and dried over anhydrous Na_2_SO_4_. The solvents were removed in vacuo and the residue was purified by flash column chromatography (PE:EA = 2:1) to afford products **9**.

**Methyl 3-(2-chloroethyl)-2-oxo-1,2,3,5-tetrahydro-4*H*-benzo[e][1,4]diazepine-4- carboxylate (9ad).** White solid (98% yield, 276.4 mg). m.p. 162–163 °C; IR (KBr) ν 3193.6, 1689.8, 1655.4 cm^−1^; ^1^H NMR (500 MHz, chloroform-*d*) δ 8.92 (br s, 1H), 7.33–7.17 (m, 2H), 7.07 (t, *J* = 7.5 Hz, 1H), 6.99 (d, *J* = 7.9 Hz, 1H), 5.15 (d, *J* = 32.3 Hz, 1H), 4.57 (s, 1H), 4.35 (d, *J* = 15.5 Hz, 1H), 3.81–3.53 (m, 5H), 2.60 (d, *J* = 46.2 Hz, 1H), 2.30 (s, 1H).; ^13^C NMR (126 MHz, chloroform-*d*) δ 172.8, 156.2, 136.4, 129.6, 129.0, 128.1, 124.0, 120.1, 59.4, 53.3, 46.7, 41.2, 35.3; HRMS (ESI) m/z [M + H]^+^ calcd. for C_13_H_16_ClN_2_O_3_, 283.0844. Found: 283.0854.

**Methyl 8-chloro-3-(2-chloroethyl)-2-oxo-1,2,3,5-tetrahydro-4*H*-benzo[e][1,4] diazepine-4-carboxylate (9bd).** White solid (98% yield, 309.7 mg). m.p. 166–168 °C; IR (KBr) ν 3184.6, 1693.5, 1660.2 cm^−1^; ^1^H NMR (500 MHz, chloroform-*d*) δ 9.11 (br s, 1H), 7.25–7.08 (m, 1H), 7.03 (d, *J* = 7.4 Hz, 2H), 5.18 (s, 1H), 4.57 (d, *J* = 31.3 Hz, 1H), 4.28 (d, *J* = 15.6 Hz, 1H), 3.69–3.63 (m, 5H), 2.65 (s, 1H), 2.31 (s, 1H); ^13^C NMR (126 MHz, chloroform-*d*) δ 173.2, 156.1, 155.6, 137.5, 134.3, 130.7, 130.2, 126.6, 124.0, 120.0, 59.5, 53.4, 46.3, 41.1, 35.2; HRMS (ESI) m/z [M + H]^+^ calcd. for C_13_H_15_Cl_2_N_2_O_3_, 317.0454. Found: 317.0462.

**Methyl 7-chloro-3-(2-chloroethyl)-2-oxo-1,2,3,5-tetrahydro-4*H*-benzo[e][1,4] diazepine-4-carboxylate (9cd).** White solid (99% yield, 312.9 mg). m.p. 171–173 °C; IR (KBr) ν 3178.1, 1696.2, 1667.5 cm^−1^; ^1^H NMR (500 MHz, chloroform-*d*) δ 8.99 (br s, 1H), 7.22 (dd, *J* = 8.4, 2.2 Hz, 2H), 6.93 (d, *J* = 8.4 Hz, 1H), 5.16 (s, 1H), 4.56 (d, *J* = 37.7 Hz, 1H), 4.30 (d, *J* = 15.6 Hz, 1H), 3.70 (s, 3H), 3.68–3.57 (m, 2H), 2.65 (s, 1H), 2.32 (s, 1H); ^13^C NMR (126 MHz, chloroform-*d*) δ 172.8, 156.1, 135.0, 129.7, 129.4, 129.0, 128.8, 121.3, 59.4, 53.5, 46.5, 41.1, 35.2; HRMS (ESI) m/z [M + H]^+^ calcd. for C_13_H_15_Cl_2_N_2_O_3_, 317.0454. Found: 317.0463.

**Methyl 3-(2-chloroethyl)-8-methyl-2-oxo-1,2,3,5-tetrahydro-4*H*-benzo[e][1,4] diazepine-4-carboxylate (9dd).** White solid (98% yield, 290.2 mg). m.p. 165–167 °C; IR (KBr) ν 3194.8, 1692.8, 1661.3 cm^−1^; ^1^H NMR (500 MHz, chloroform-*d*) δ 8.54 (br s, 1H), 7.13 (d, *J* = 25.1 Hz, 1H), 6.88 (d, *J* = 7.6 Hz, 1H), 6.76 (s, 1H), 5.23–5.01 (m, 1H), 4.54 (s, 1H), 4.33 (d, *J* = 15.4 Hz, 1H), 3.76–3.53 (m, 5H), 2.56 (d, *J* = 41.1 Hz, 1H), 2.32 (s, 4H); ^13^C NMR (126 MHz, chloroform-*d*) δ 172.5, 156.2, 139.1, 136.1, 129.5, 129.1, 125.2, 120.5, 59.4, 53.3, 46.6, 41.3, 35.3, 21.2; HRMS (ESI) m/z [M + H]^+^ calcd. for C_14_H_18_ClN_2_O_3_, 297.1000. Found: 297.1009.

### 4.7. General Procedure for the Scale-Up Synthesis of Compound ***9ad***

To a flask (100 mL) equipped with a magnetic stirrer were added compound **7a** (5.0 mmol, 0.94 g, 1.0 equiv) and anhydrous CH_3_CN (50 mL).Then, methyl chloroformate (10.0 mmol, 0.95 mL, 2.0 equiv) was added dropwise. After the addition, the reaction mixture was stirred at reflux for about 2 h until the substrate disappeared, and then saturated NaHCO_3_ aqueous solution (50 mL) was added to quench the reaction. The mixture was extracted with ethyl acetate (3 × 80 mL), and the combined organic layers were washed with brine (1 × 200 mL) and dried over anhydrous Na_2_SO_4_. The solvents were removed in vacuo and the residue was purified by flash column chromatography (PE:EA = 2:1) to afford 1.35 g of target product **9ad** in a 96% yield.

## Figures and Tables

**Table 1:** Optimization of the ring-opening reaction of trifluoromethanesulfonate **8d** ^1^.

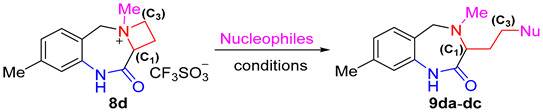					
Entry	Nucleophile (2.0 equiv)	Solvent	T (°C)	t (h)	Yield ^2^ (%)
1	NaN_3_	DMF	rt	6	91
2	NaN_3_	THF	rt	12	84
3	NaN_3_	DCM	rt	12	62
4	KCN	DMF	rt	12	64
5	KCN	DMF	rt	24	78
6	PhSNa	DMF	rt	12	95
7	PhOH	DMF	rt	24	N.R. ^3^
8	PhOH	DMF	reflux	24	trace
9	PhONa	DMF	rt	24	N.R. ^3^
10	PhONa	DMF	reflux	24	trace

^1^ Reaction conditions: compound **8d** (1.0 mmol, 1.0 equiv), nucleophile (2.0 mmol, 2.0 equiv) and solvent (5.0 mL) were added, and then the mixture was stirred at the corresponding temperature. ^2^ Yield of isolated product. ^3^ No reaction.

## Data Availability

The original contributions presented in this study are included in the article/Supplementary Material. Further inquiries can be directed to the corresponding authors.

## Supplementary Materials

The crystallographic data of **9aa** and the NMR (^1^H, ^13^C) spectra of all the new compounds can be downloaded at https://www.mdpi.com/article/10.3390/molecules30092014/s1.
