# Supplementary material for: Synthesis of 1,4-Benzodiazepines via Intramolecular C–N Bond Coupling and Ring Opening of Azetidines
Source: Molecules. 2025 Apr 30;30(9):2014. doi: 10.3390/molecules30092014 (PMC12073993; doi:10.3390/molecules30092014)

checkCIF/PLATON report

You have not supplied any structure factors. As a result the full set of tests cannot be run.

THIS REPORT IS FOR GUIDANCE ONLY. IF USED AS PART OF A REVIEW PROCEDURE FOR PUBLICATION, IT SHOULD NOT REPLACE THE EXPERTISE OF AN EXPERIENCED CRYSTALLOGRAPHIC REFEREE.

No syntax errors found. CIF dictionary Interpreting this report

Datablock: a

```
Bond precision:  C-C = 0.0048 A          Wavelength=0.71073

Cell:            a=12.762(3)     b=5.9010(12)       c=17.294(4)
                 alpha=90        beta=105.24(3)     gamma=90
Temperature:     293 K

                Calculated                Reported
Volume          1256.6(5)                 1256.6(4)
Space group     P 21/n                    P 21/n
Hall group      -P 2yn                    ?
Moiety formula  C12 H15 N5 O              C12 H15 N5 O
Sum formula     C12 H15 N5 O              C12 H15 N5 O
Mr              245.29                    245.29
Dx,g cm-3       1.297                     1.297
Z               4                         4
Mu (mm-1)       0.088                     0.088
F000            520.0                     520.0
F000'           520.17
h,k,lmax        15,7,20                   15,7,20
Nref            2265                      2258
Tmin,Tmax       0.989,0.993
Tmin'           0.989

Correction method= Not given

Data completeness= 0.997          Theta(max)= 25.250

R(reflections)= 0.0860( 1972)     wR2(reflections)= 0.2134( 2258)

S = 1.310                  Npar= 163
```

The following ALERTS were generated. Each ALERT has the format
test-name_ALERT_alert-type_alert-level.
Click on the hyperlinks for more details of the test.

Alert level C

```
PLAT052_ALERT_1_C Info on Absorption Correction Method   Not Given     Please Do !
PLAT199_ALERT_1_C Reported _cell_measurement_temperature ..... (K)        293 Check
PLAT200_ALERT_1_C Reported   _diffrn_ambient_temperature ..... (K)        293 Check
PLAT242_ALERT_2_C Low    'MainMol' Ueq as Compared to Neighbors of         N2 Check
PLAT340_ALERT_3_C Low Bond Precision on  C-C Bonds ..............     0.0048 Ang.
```

Alert level G

```
PLAT005_ALERT_5_G No Embedded Refinement Details Found  in the CIF     Please Do !
PLAT007_ALERT_5_G Number of Unrefined Donor-H Atoms .............          1 Report
PLAT093_ALERT_1_G No s.u.'s on H-positions, Refinement Reported as      mixed Check
PLAT710_ALERT_4_G Delete 1-2-3 or 2-3-4 Linear Torsion Angle ... #          1 Do !
              N1  -N2  -N3  -C1   -180.00  0.01   1.555   1.555   1.555   1.555
PLAT793_ALERT_4_G Model has Chirality at C3          (Centro SPGR)          S Verify
PLAT899_ALERT_4_G SHELXL97  is Deprecated and Succeeded by SHELXL/       2018 Note
```

---

0 **ALERT level A** = Most likely a serious problem - resolve or explain
0 **ALERT level B** = A potentially serious problem, consider carefully
5 **ALERT level C** = Check. Ensure it is not caused by an omission or oversight
6 **ALERT level G** = General information/check it is not something unexpected

4 ALERT type 1 CIF construction/syntax error, inconsistent or missing data
1 ALERT type 2 Indicator that the structure model may be wrong or deficient
1 ALERT type 3 Indicator that the structure quality may be low
3 ALERT type 4 Improvement, methodology, query or suggestion
2 ALERT type 5 Informative message, check

It is advisable to attempt to resolve as many as possible of the alerts in all categories. Often the minor alerts point to easily fixed oversights, errors and omissions in your CIF or refinement strategy, so attention to these fine details can be worthwhile. In order to resolve some of the more serious problems it may be necessary to carry out additional measurements or structure refinements. However, the purpose of your study may justify the reported deviations and the more serious of these should normally be commented upon in the discussion or experimental section of a paper or in the "special_details" fields of the CIF. checkCIF was carefully designed to identify outliers and unusual parameters, but every test has its limitations and alerts that are not important in a particular case may appear. Conversely, the absence of alerts does not guarantee there are no aspects of the results needing attention. It is up to the individual to critically assess their own results and, if necessary, seek expert advice.

Publication of your CIF in IUCr journals

A basic structural check has been run on your CIF. These basic checks will be run on all CIFs submitted for publication in IUCr journals (*Acta Crystallographica*, *Journal of Applied Crystallography*, *Journal of Synchrotron Radiation*); however, if you intend to submit to *Acta Crystallographica Section C* or *E* or *IUCrData*, you should make sure that full publication checks are run on the final version of your CIF prior to submission.

Publication of your CIF in other journals

Please refer to the *Notes for Authors* of the relevant journal for any special instructions relating to CIF submission.

PLATON version of 18/09/2020; check.def file version of 20/08/2020

Datablock a - ellipsoid plot

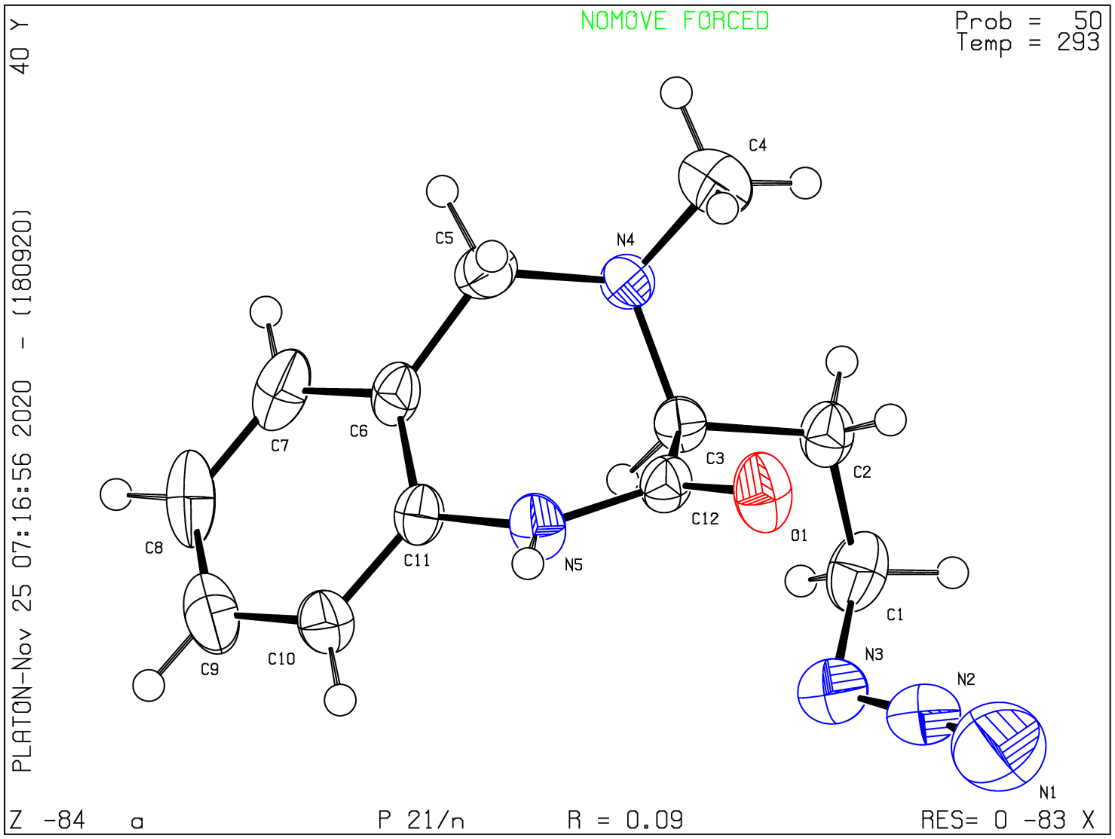

Supplement: Supplementary file 1 [file molecules-30-02014-s001.zip › checkcif.pdf]
