# Supplementary material for: Synthesis of 1,4-Benzodiazepines via Intramolecular C–N Bond Coupling and Ring Opening of Azetidines
Source: Molecules. 2025 Apr 30;30(9):2014. doi: 10.3390/molecules30092014 (PMC12073993; doi:10.3390/molecules30092014)
Supplement: Supplementary file 1 [file molecules-30-02014-s001.zip › Supporting Information.pdf]

Supplementary Materials

Synthesis of 1,4-Benzodiazepines via Intramolecular C-N Bond Coupling and Ring-Opening of Azetidines

Xin-Ming Xu[1,*], Sen Chen[1], Shao-Lei Duan[1], Xiang-Min Wang[1,*], Qian Liu[2], Kai Sun[1,*]

[1] School of Chemistry and Chemical Engineering, Yantai University, Yantai 264005, China

[2] School of Life Sciences, Yantai University, Yantai 264005, China

***** Correspondence: xin_mingxu@163.com (X.-M. X.); sailing616@126.com (X.-M. W.); sunk468@nenu.edu.cn (K. S.)

1. General information ………………………………………………………… S2

2. Crystallographic data of 9aa ………………………………………………… S2

3. Copies of ^{1}H and ^{13}C NMR Spectra … ………………………………… S4

1. General information

A range of 2-((2-bromobenzyl)(2-chloroethyl)amino)acetonitrile was prepared according to reported literature procedures. Methyl triflate, methyl chloroformate and other reagents were purchased from commercial domestic chemical companies. Anhydrous *tert*-butanol, tetrahydrofuran, 1,4-dioxane, dichloromethane, N,N-dimethylformamide, and acetonitrile were dried or purified according to standard procedures prior to use. Reactions were monitored using pre-coated, glass-backed silica gel plates and visualized by means of UV irridation (254 nm) or $KMnO_4$, phosphomolybdic acid, and ninhydrine. Infrared (IR) spectra were obtained with thin film samples on a PerkinElmer Spectrum Two spectrometer, and data are expressed in cm^{-1}. ^{1}H NMR and ^{13}C NMR spectra were recorded using 400 MHz spectrometers at ambient temperature. Chemical shifts are reported in ppm with either tetramethylsilane or the residual solvent resonance used as an internal standard. Abbreviations are used in the description of NMR data as follows: chemical shift (δ, ppm), multiplicity (s = singlet, d = doublet, t = triplet, m = multiplet), coupling constant (*J*, Hz). Mass spectra was measured using mass spectrometers. All yields reported were isolated yields.

2. Crystallographic Data of 9aa

High quality single crystals of **9aa** were cultivated from the evaporation of a solution of **9aa** in the mixture of EtOAc and *n*-hexane. As depicted below, the molecular structures were determined by X-ray diffraction analysis.

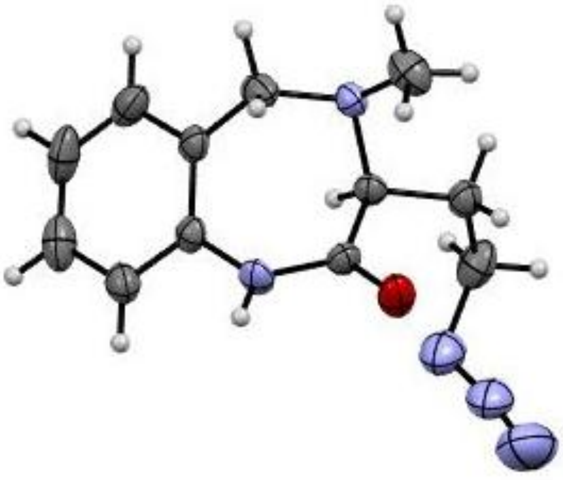

Figure S1. X-ray molecular structure of **9aa**. The molecular structure is depicted in an ellipsoid style at 50% probability level.

2.1 Crystallographic data and structure refinement of 9aa

CCDC Number	2046809
Empirical formula	$C_{12}H_{15}N_5O$
Formula weight	245.29
Temperature	293.00 K
Wavelength	0.71073 Å
Crystal system	monoclinic
Space group	P 21/n
a	12.762 (3) Å
b	5.901 (12) Å
c	17.294 (4) Å
α	90 °
β	105.24 (3) °
γ	90 °
Volumn	1256.6 (4) $Å^3$
Z	4
Density (calculated)	1.297
Absorption coefficient	0.088 mm^{-1}
F(000)	520.0
Radiation type	MoK\a
Data completeness	99.7%
Theta (max)	25.250 °
R (reflections)	0.0860 (1972)
wR2 (reflections)	0.2134 (2258)

3. Copies of ^{1}H NMR and ^{13}C NMR Spectra of Products

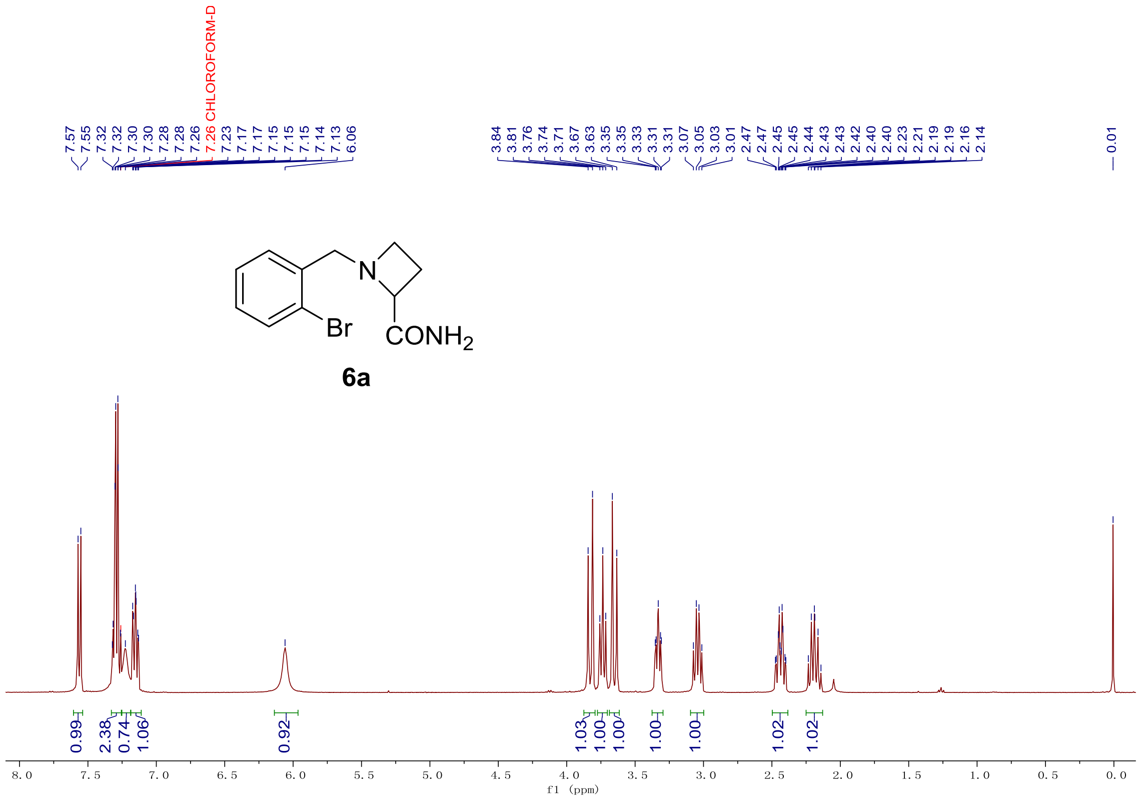

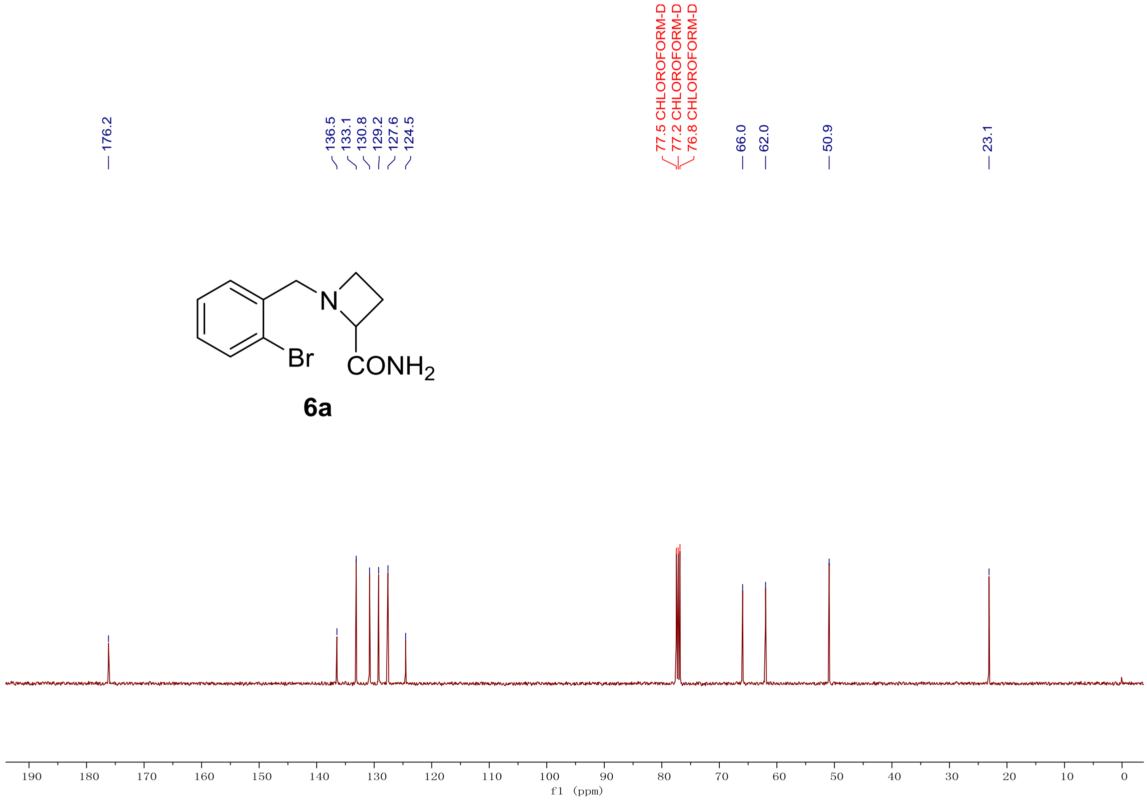

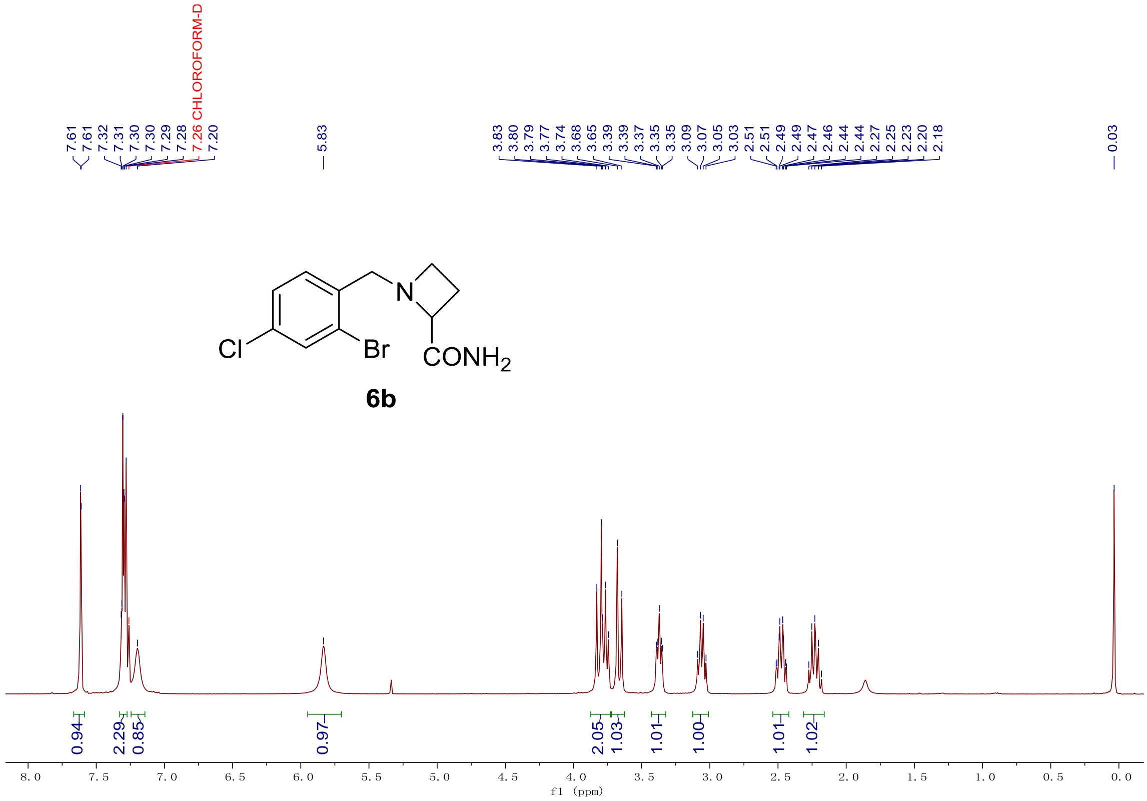
7.61
7.61
7.32
7.31
7.30
7.30
7.29
7.28
7.26 CHLOROFORM-D
7.20
5.83
3.83
3.80
3.79
3.77
3.74
3.68
3.65
3.39
3.39
3.37
3.35
3.35
3.09
3.07
3.05
3.03
2.51
2.51
2.49
2.49
2.47
2.46
2.44
2.44
2.27
2.25
2.23
2.20
2.18
0.03
Cl
Br
N
CONH2
6b
0.94
2.29
0.85
0.97
2.05
1.03
1.01
1.00
1.01
1.02
f1 (ppm)

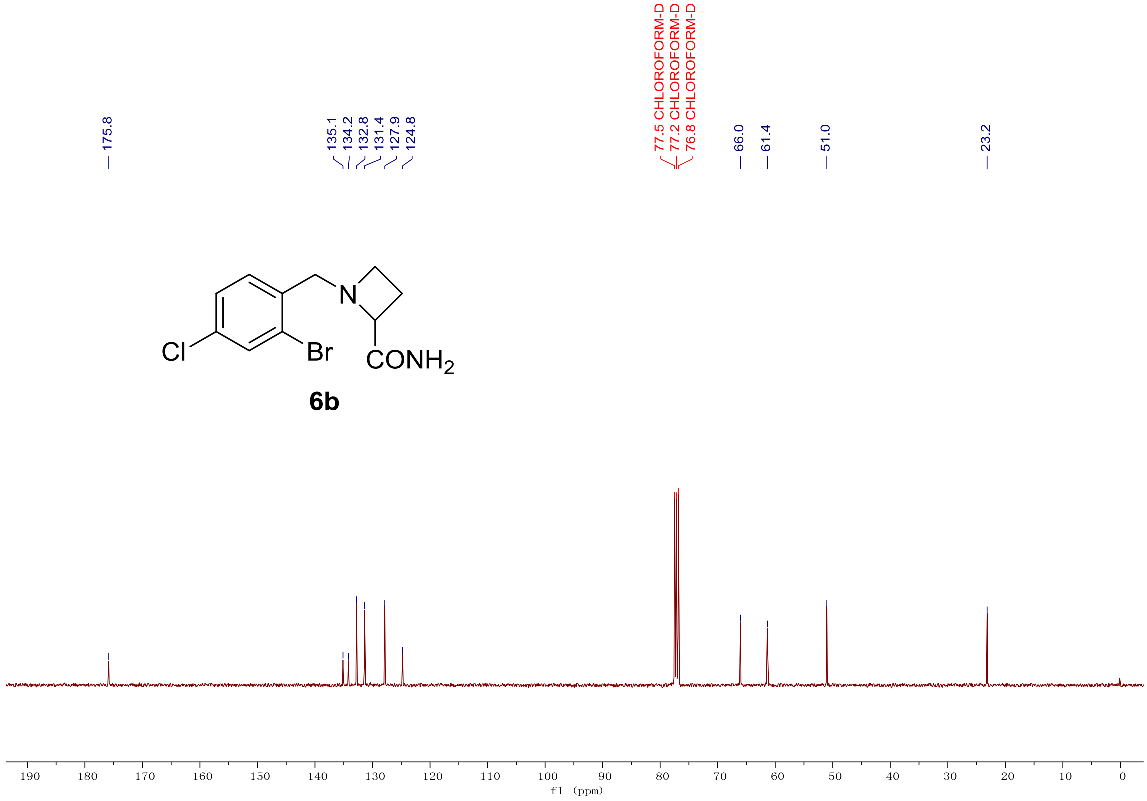
175.8
135.1
134.2
132.8
131.4
127.9
124.8
77.5 CHLOROFORM-D
77.2 CHLOROFORM-D
76.8 CHLOROFORM-D
66.0
61.4
51.0
23.2
Cl
Br
N
CONH2
6b
f1 (ppm)

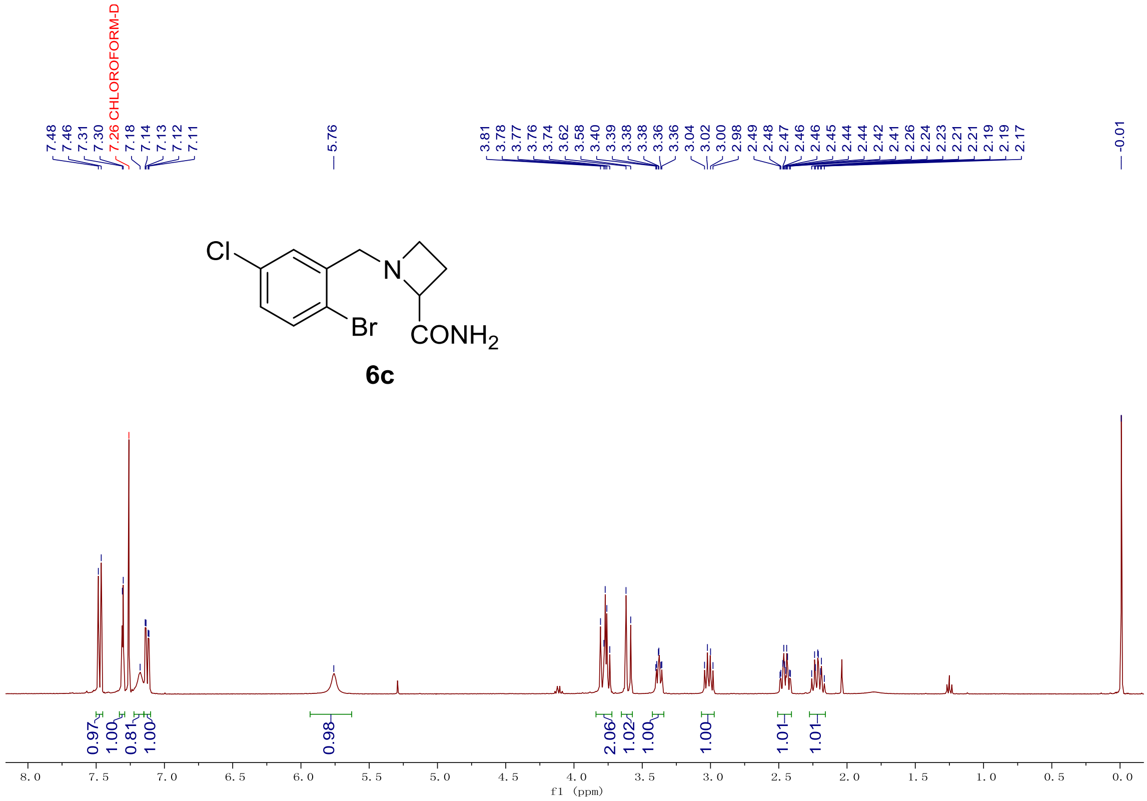
7.48
7.46
7.31
7.30
7.26 CHLOROFORM-D
7.18
7.14
7.13
7.12
7.11
5.76
3.81
3.78
3.77
3.76
3.74
3.62
3.58
3.40
3.39
3.38
3.38
3.36
3.36
3.04
3.02
3.00
2.98
2.49
2.48
2.47
2.46
2.46
2.45
2.44
2.44
2.42
2.41
2.26
2.24
2.23
2.21
2.21
2.19
2.17
-0.01
Cl
N
Br
CONH2
6c
0.97
1.00
0.81
1.00
0.98
2.06
1.02
1.00
1.00
1.01
1.01
f1 (ppm)

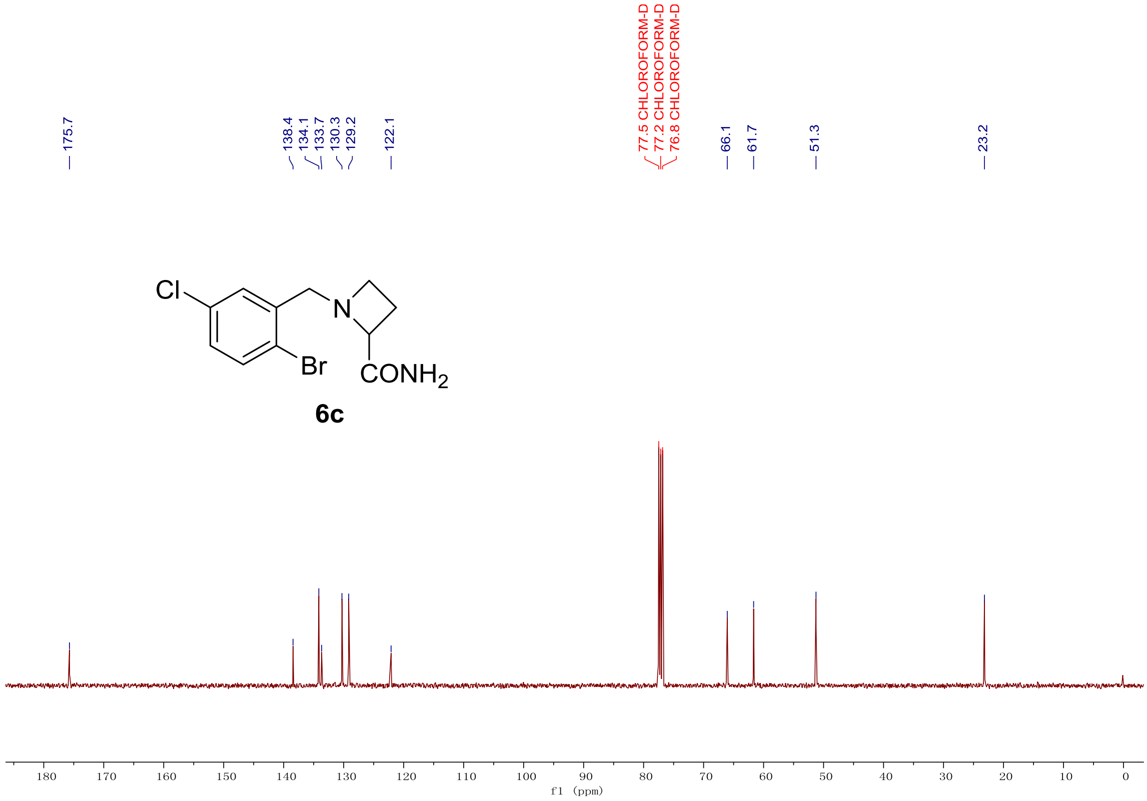
175.7
138.4
134.1
133.7
130.3
129.2
122.1
77.5 CHLOROFORM-D
77.2 CHLOROFORM-D
76.8 CHLOROFORM-D
66.1
61.7
51.3
23.2
Cl
N
Br
CONH2
6c
f1 (ppm)

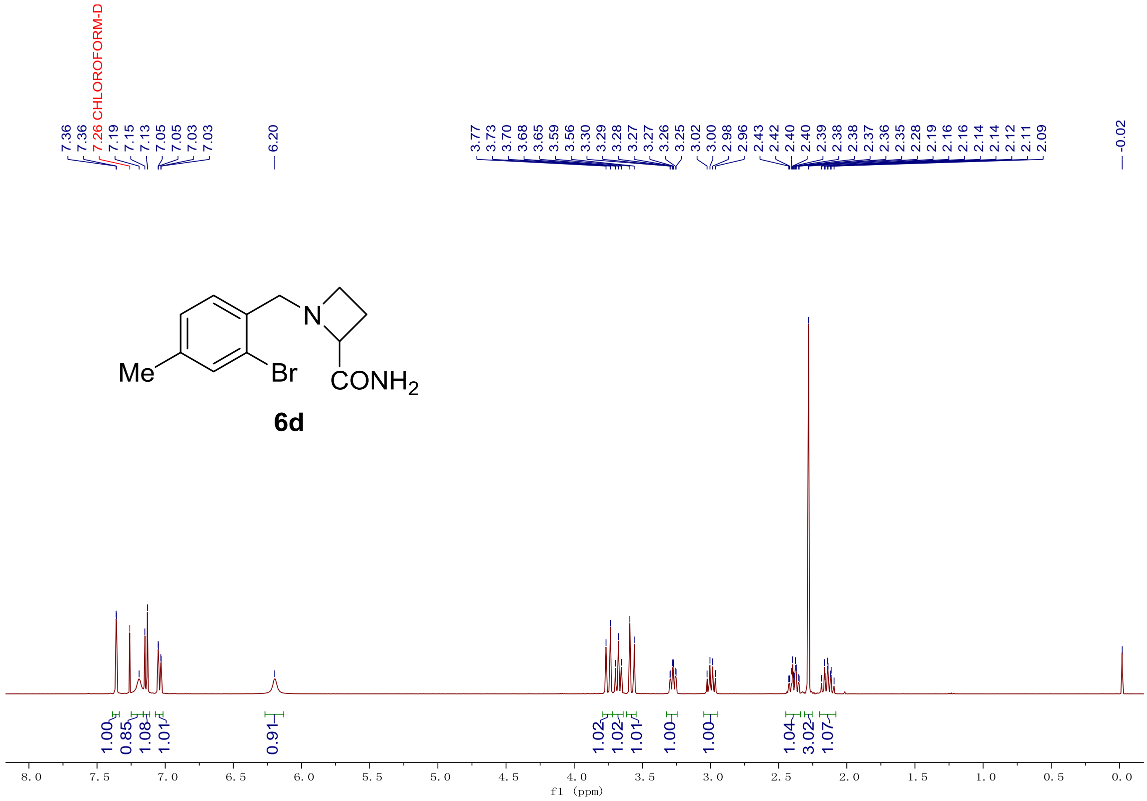
7.36
7.36
7.26 CHLOROFORM-D
7.19
7.15
7.13
7.05
7.05
7.03
7.03
6.20
3.77
3.73
3.70
3.68
3.65
3.59
3.56
3.30
3.29
3.28
3.27
3.27
3.26
3.25
3.02
3.00
2.98
2.96
2.43
2.42
2.40
2.40
2.39
2.38
2.38
2.37
2.36
2.35
2.28
2.19
2.16
2.16
2.14
2.14
2.12
2.11
2.09
-0.02
N
Me
Br
CONH2
6d
1.00
0.85
1.08
1.01
0.91
1.02
1.02
1.01
1.00
1.00
1.04
3.02
1.07
f1 (ppm)

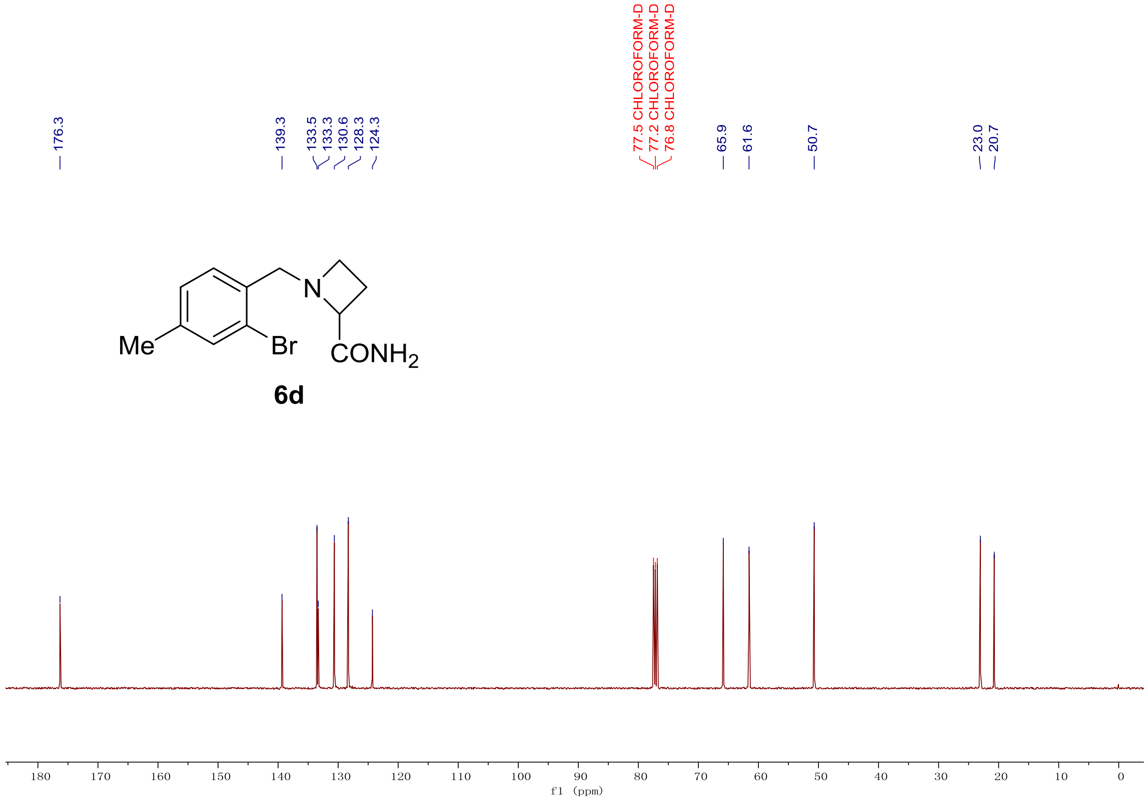
176.3
139.3
133.5
133.3
130.6
128.3
124.3
77.5 CHLOROFORM-D
77.2 CHLOROFORM-D
76.8 CHLOROFORM-D
65.9
61.6
50.7
23.0
20.7
N
Me
Br
CONH2
6d
f1 (ppm)

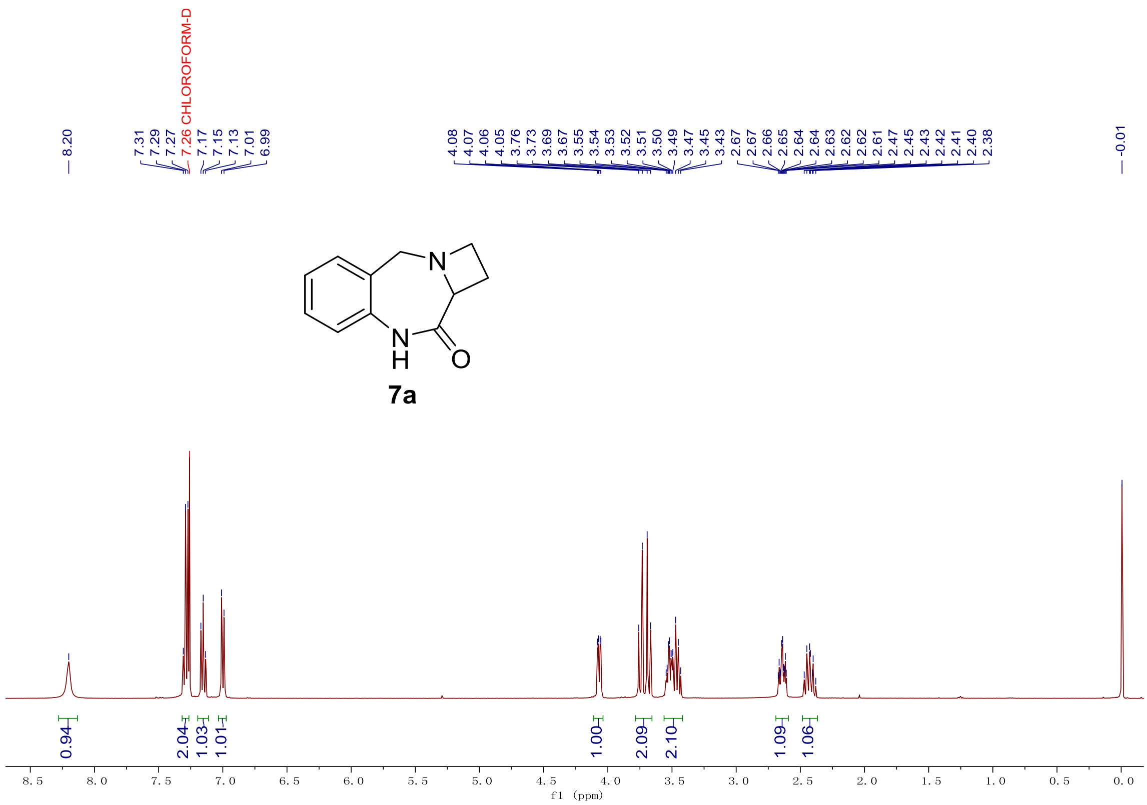
8.20
7.31
7.29
7.27
7.26 CHLOROFORM-D
7.17
7.15
7.13
7.01
6.99
4.08
4.07
4.06
4.05
3.76
3.73
3.69
3.67
3.55
3.54
3.53
3.52
3.51
3.50
3.49
3.47
3.45
3.43
2.67
2.67
2.66
2.65
2.64
2.64
2.63
2.62
2.62
2.61
2.47
2.45
2.43
2.42
2.41
2.40
2.38
-0.01
7a
0.94
2.04
1.03
1.01
1.00
2.09
2.10
1.09
1.06
f1 (ppm)

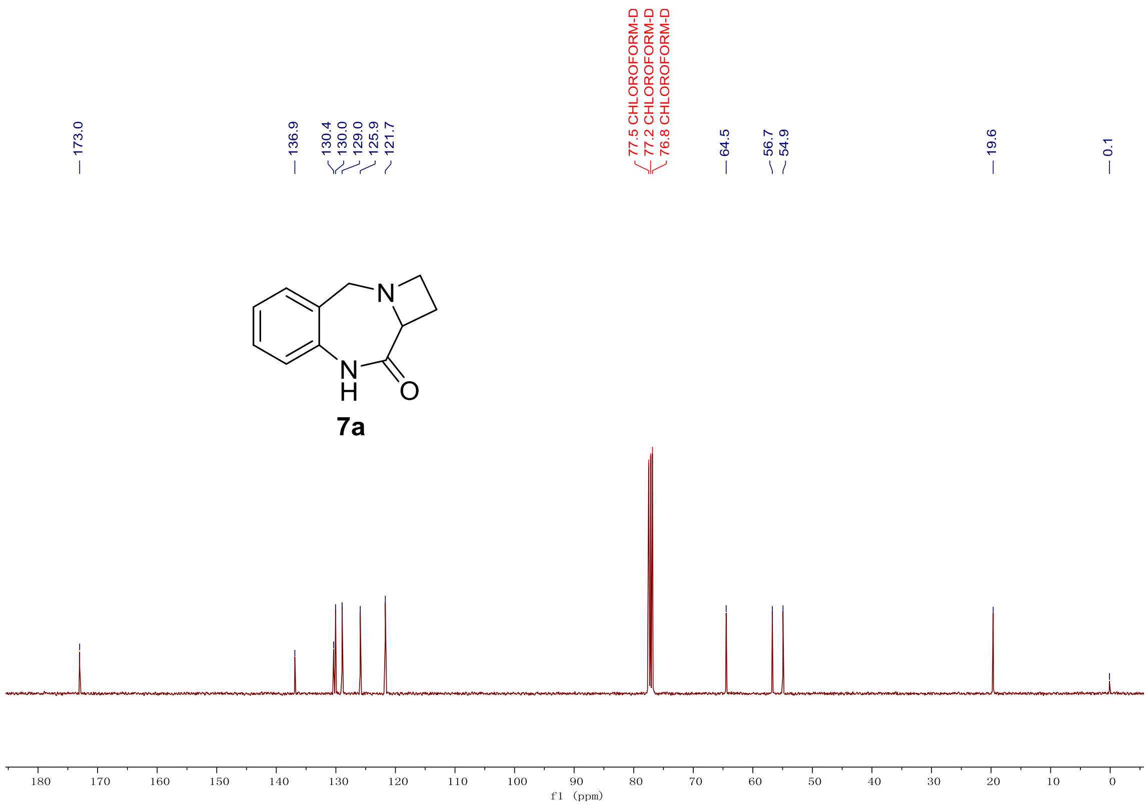
173.0
136.9
130.4
130.0
129.0
125.9
121.7
77.5 CHLOROFORM-D
77.2 CHLOROFORM-D
76.8 CHLOROFORM-D
64.5
56.7
54.9
19.6
0.1
7a
f1 (ppm)

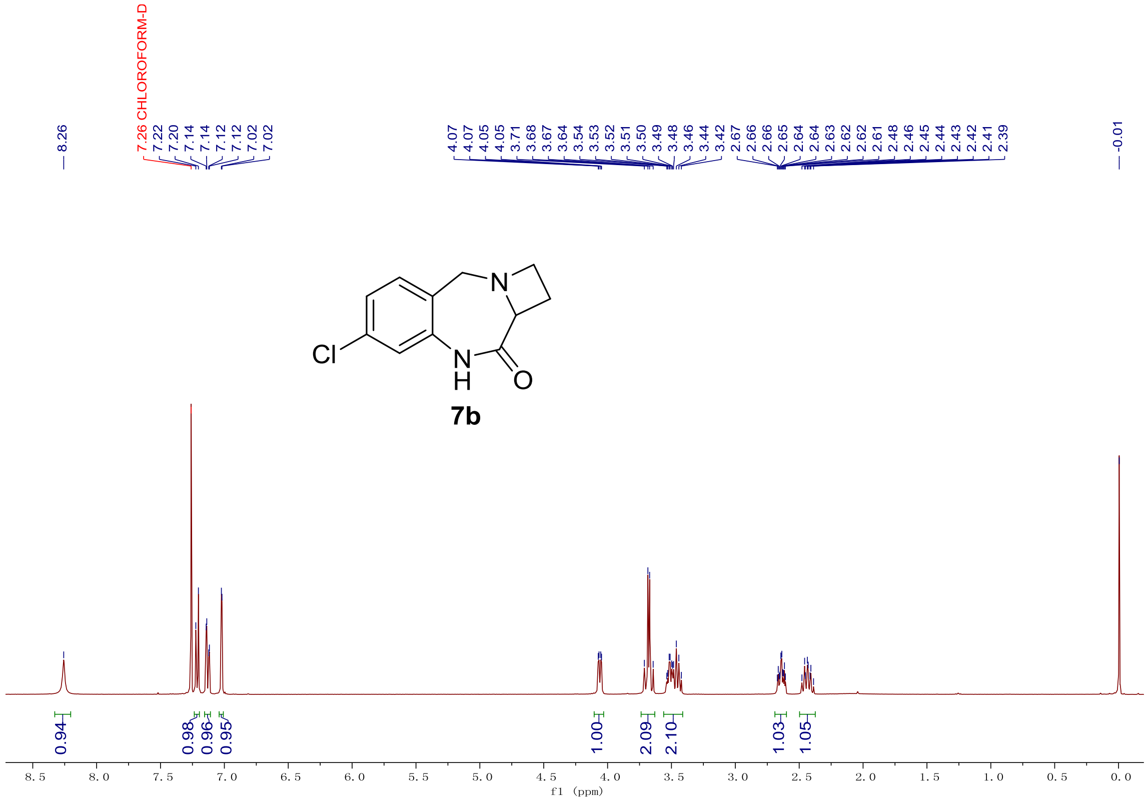
8.26
7.26 CHLOROFORM-D
7.22
7.20
7.14
7.14
7.12
7.12
7.02
7.02
4.07
4.07
4.05
3.71
3.68
3.67
3.64
3.54
3.53
3.52
3.51
3.50
3.49
3.48
3.46
3.44
3.42
2.67
2.66
2.65
2.64
2.64
2.63
2.62
2.62
2.61
2.48
2.46
2.45
2.44
2.43
2.42
2.41
2.39
-0.01
N
Cl
N
H
O
7b
0.94
0.98
0.96
0.95
1.00
2.09
2.10
1.03
1.05
8.5 8.0 7.5 7.0 6.5 6.0 5.5 5.0 4.5 4.0 3.5 3.0 2.5 2.0 1.5 1.0 0.5 0.0
f1 (ppm)

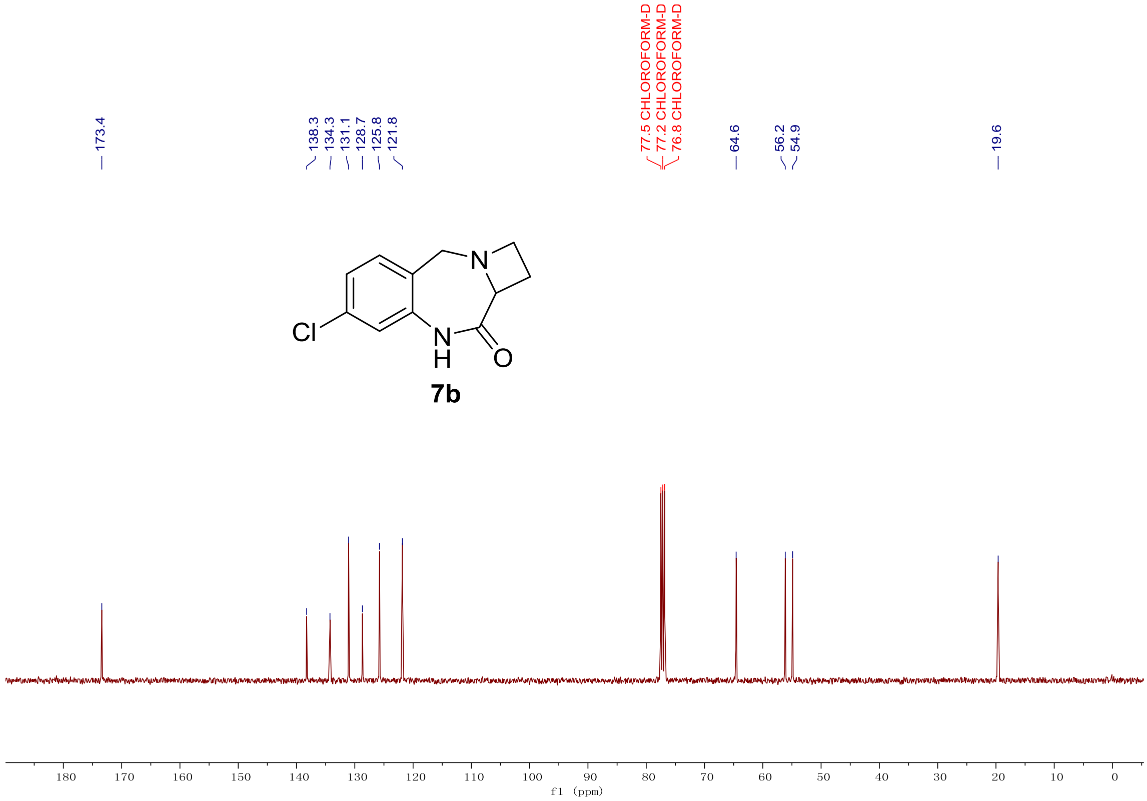
173.4
138.3
134.3
131.1
128.7
125.8
121.8
77.5 CHLOROFORM-D
77.2 CHLOROFORM-D
76.8 CHLOROFORM-D
64.6
56.2
54.9
19.6
N
Cl
N
H
O
7b
180 170 160 150 140 130 120 110 100 90 80 70 60 50 40 30 20 10 0
f1 (ppm)

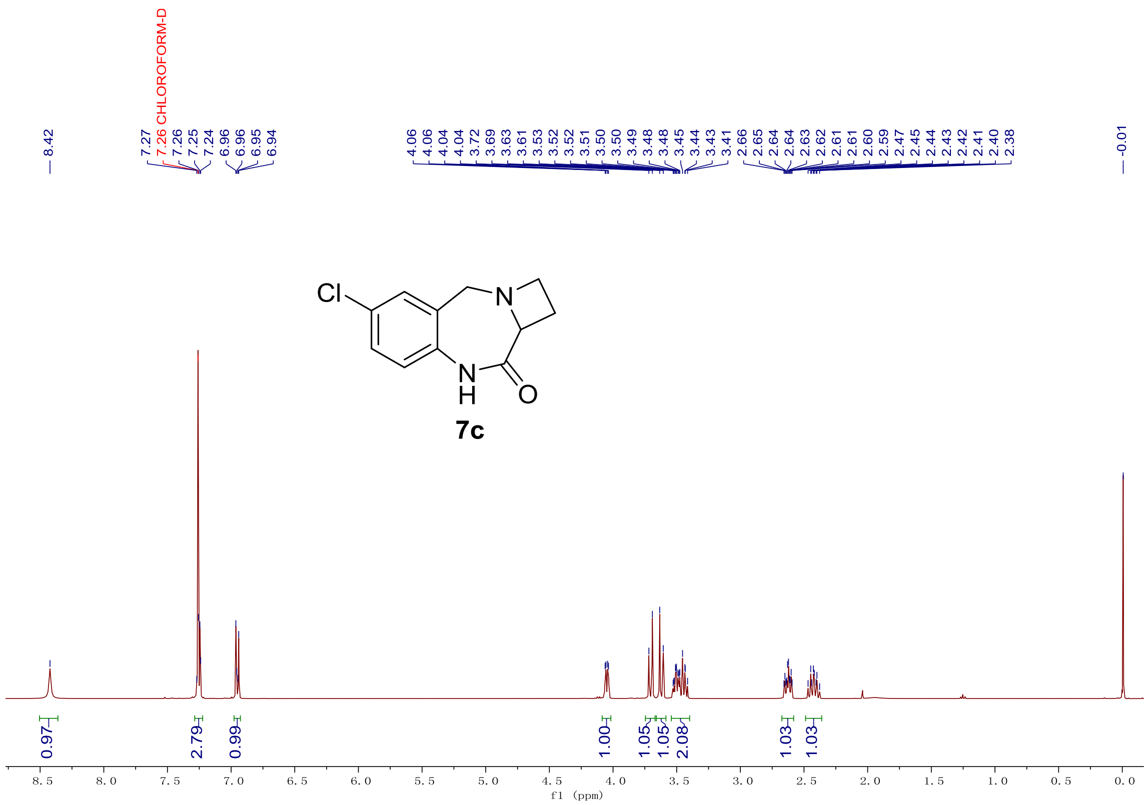
8.42
7.27
7.26 CHLOROFORM-D
7.26
7.25
7.24
6.96
6.96
6.95
6.94
4.06
4.06
4.04
4.04
3.72
3.69
3.63
3.61
3.53
3.52
3.52
3.51
3.50
3.50
3.49
3.48
3.48
3.45
3.44
3.43
3.41
2.66
2.65
2.64
2.64
2.63
2.62
2.61
2.61
2.60
2.59
2.47
2.45
2.44
2.43
2.42
2.41
2.40
2.38
-0.01
Cl
N
H
N
O
7c
0.97
2.79
0.99
1.00
1.05
1.05
2.08
1.03
1.03
f1 (ppm)

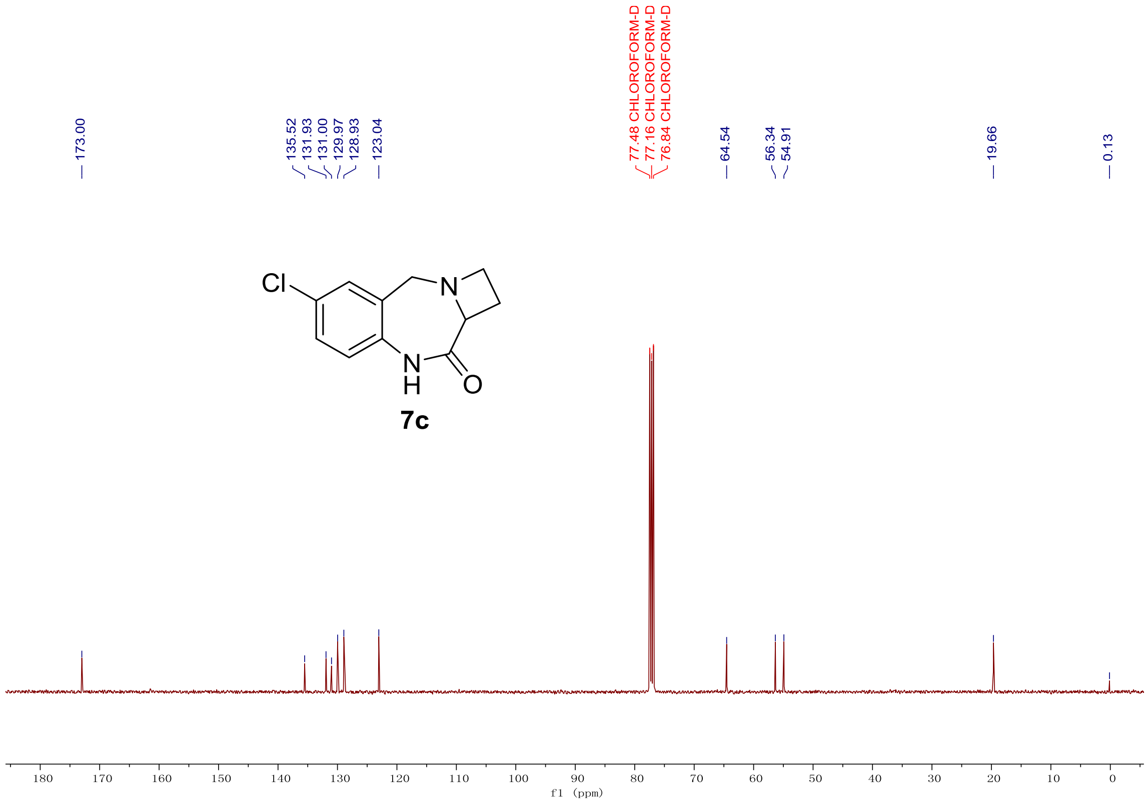
173.00
135.52
131.93
131.00
129.97
128.93
123.04
77.48 CHLOROFORM-D
77.16 CHLOROFORM-D
76.84 CHLOROFORM-D
64.54
56.34
54.91
19.66
0.13
Cl
N
H
N
O
7c
f1 (ppm)

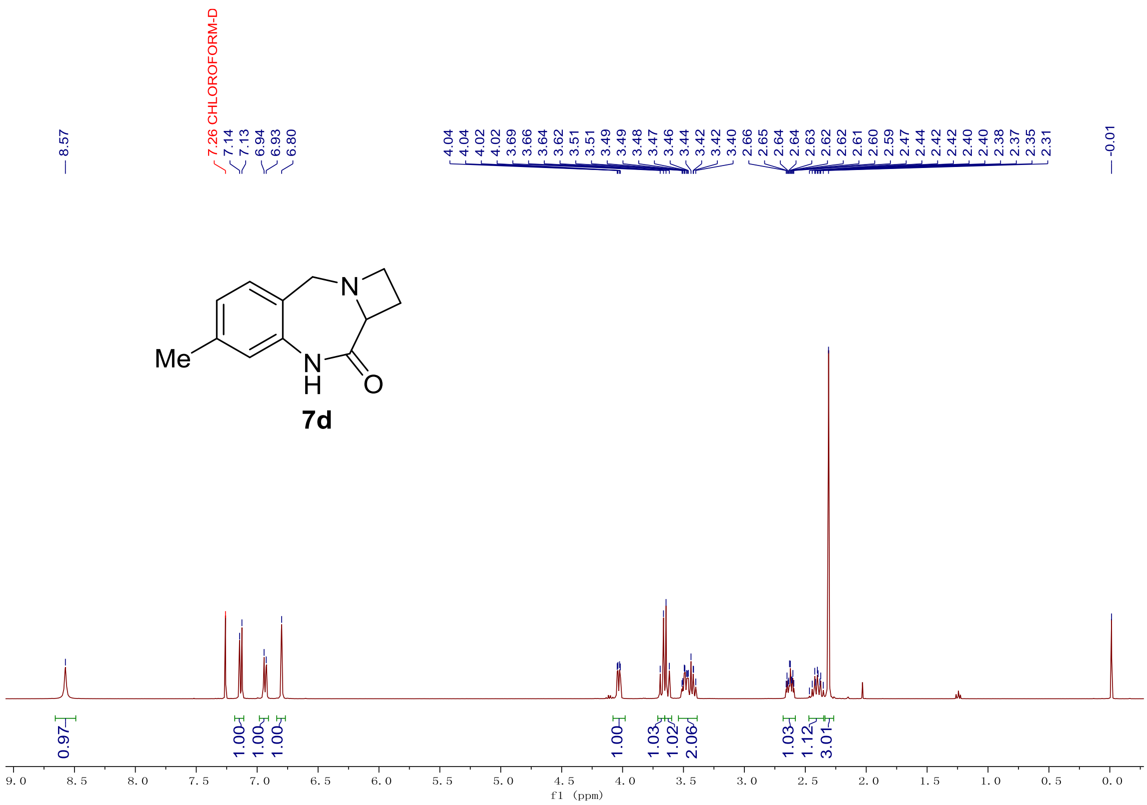
7.26 CHLOROFORM-D
7d
Me
N
H
O
f1 (ppm)

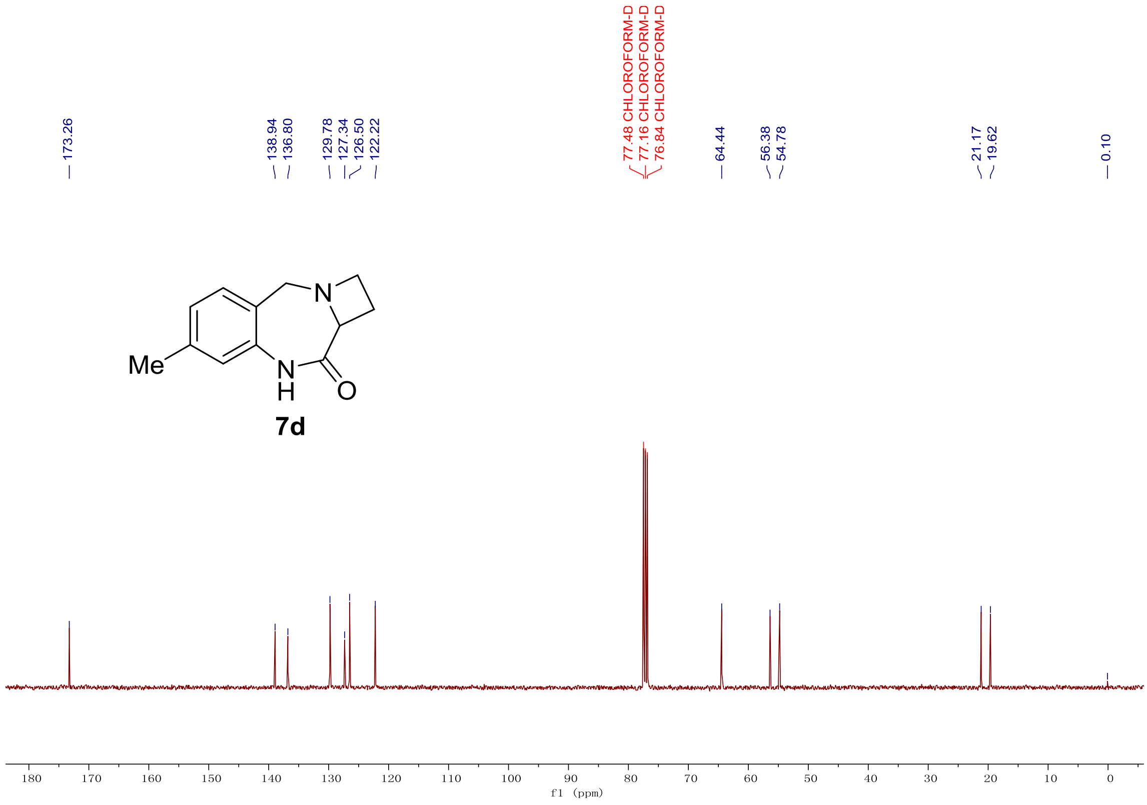
77.48 CHLOROFORM-D
77.16 CHLOROFORM-D
76.84 CHLOROFORM-D
173.26
138.94
136.80
129.78
127.34
126.50
122.22
64.44
56.38
54.78
21.17
19.62
0.10
7d
Me
f1 (ppm)

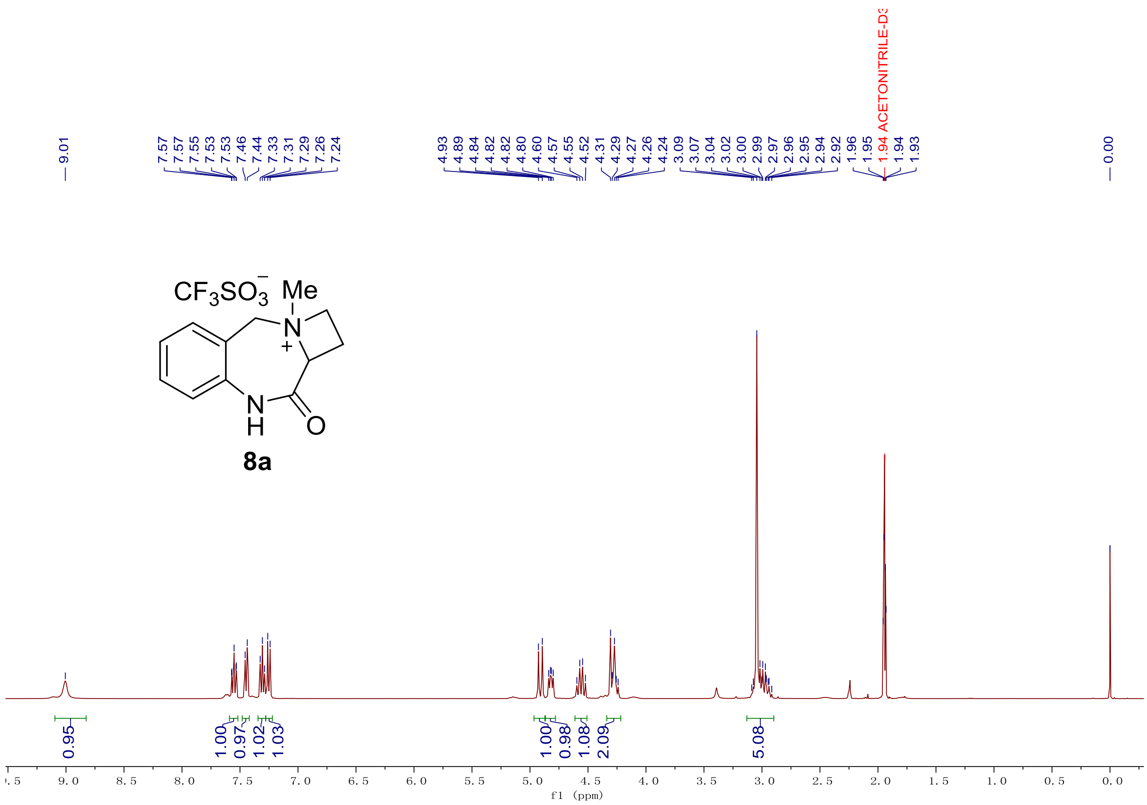
9.01
7.57 7.57 7.55 7.53 7.53 7.46 7.44 7.33 7.31 7.29 7.26 7.24
4.93 4.89 4.84 4.82 4.82 4.80 4.60 4.57 4.55 4.52 4.31 4.29 4.27 4.26 4.24
3.09 3.07 3.04 3.02 3.00 2.99 2.97 2.96 2.95 2.94 2.92
1.96 1.95 1.94 ACETONITRILE-D3 1.94 1.93
0.00
CF3SO3− Me
N+
N H O
8a
0.95 1.00 0.97 1.02 1.03 1.00 0.98 1.08 2.09 5.08
f1 (ppm)

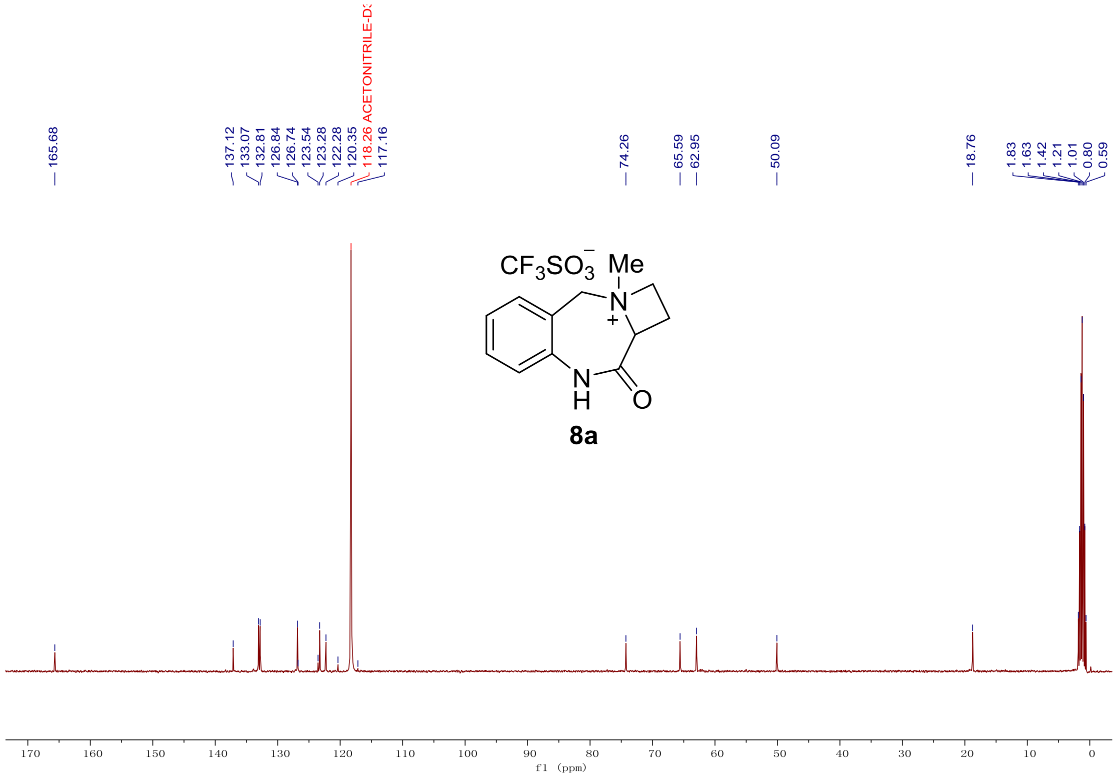
165.68
137.12 133.07 132.81 126.84 126.74 123.54 123.28 122.28 120.35 118.26 ACETONITRILE-D3 117.16
74.26
65.59 62.95
50.09
18.76
1.83 1.63 1.42 1.21 1.01 0.80 0.59
CF3SO3− Me
N+
N H O
8a
f1 (ppm)

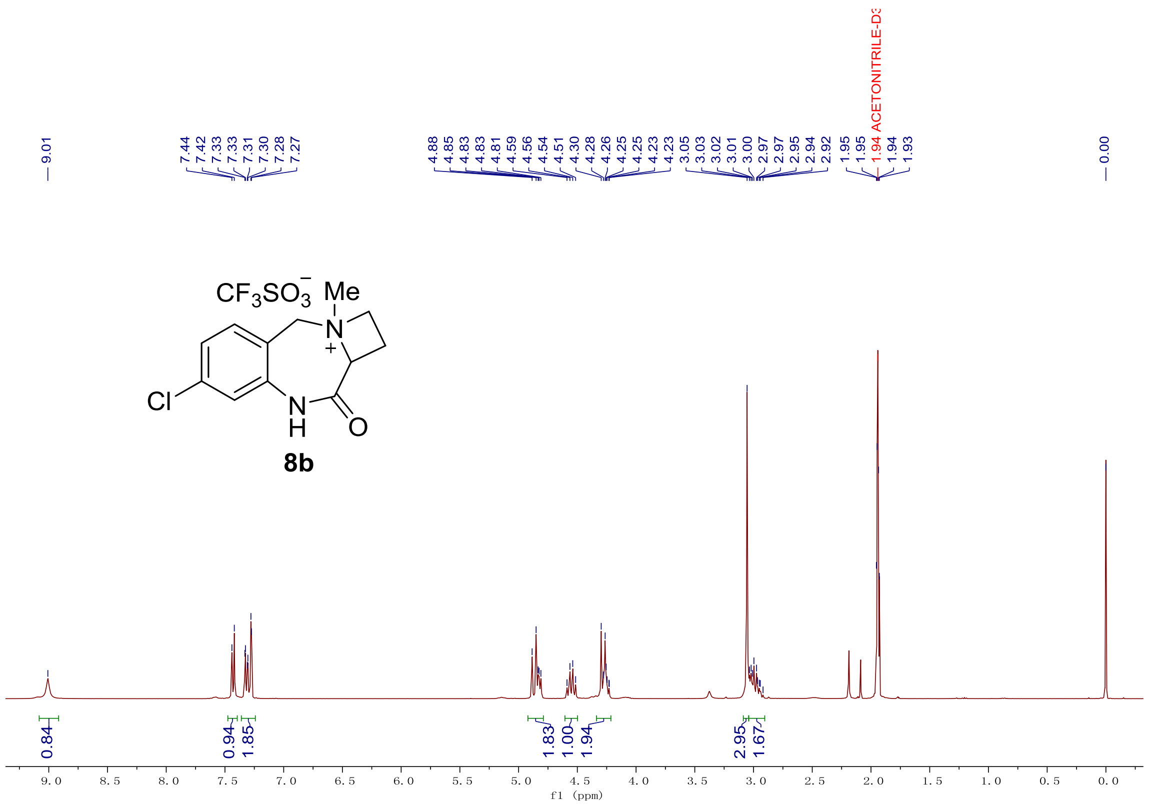

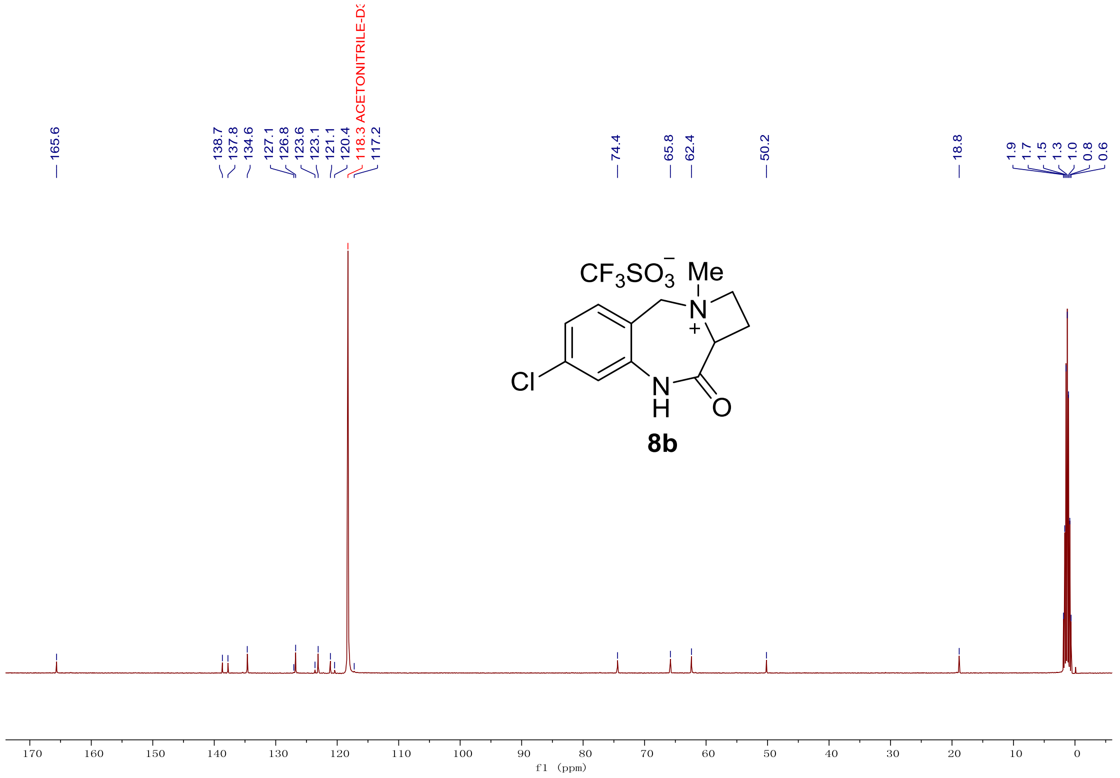

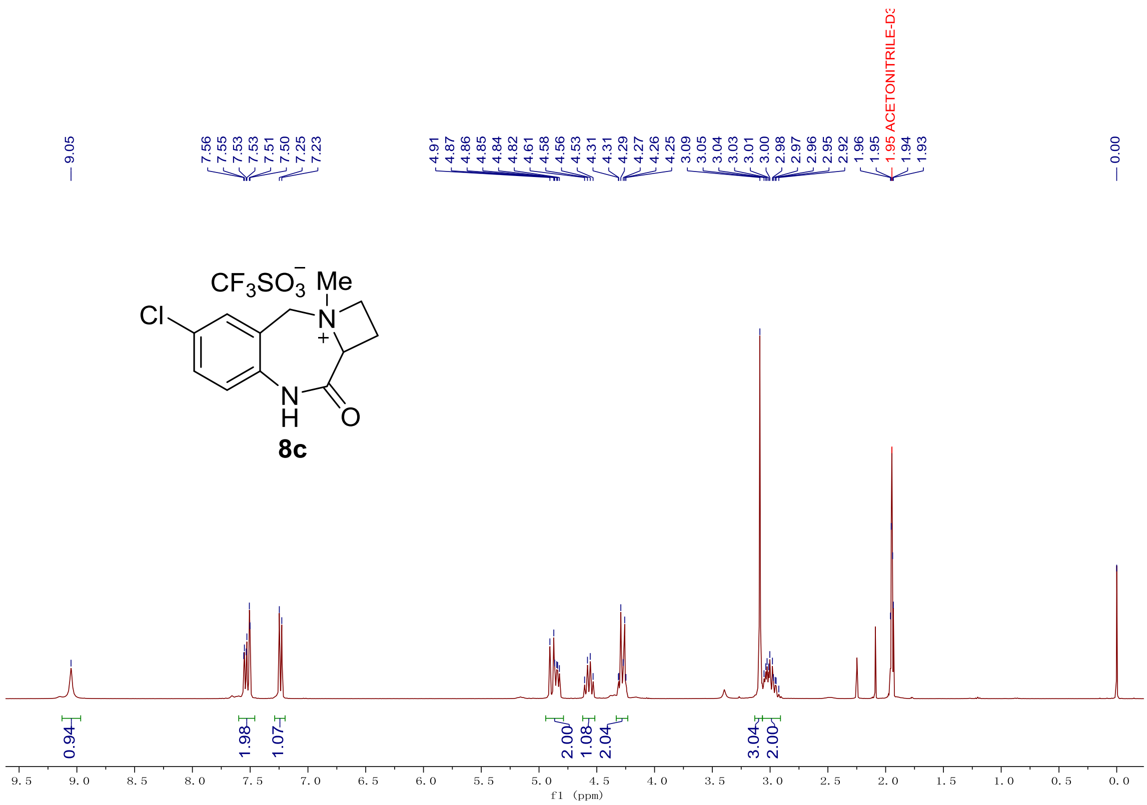

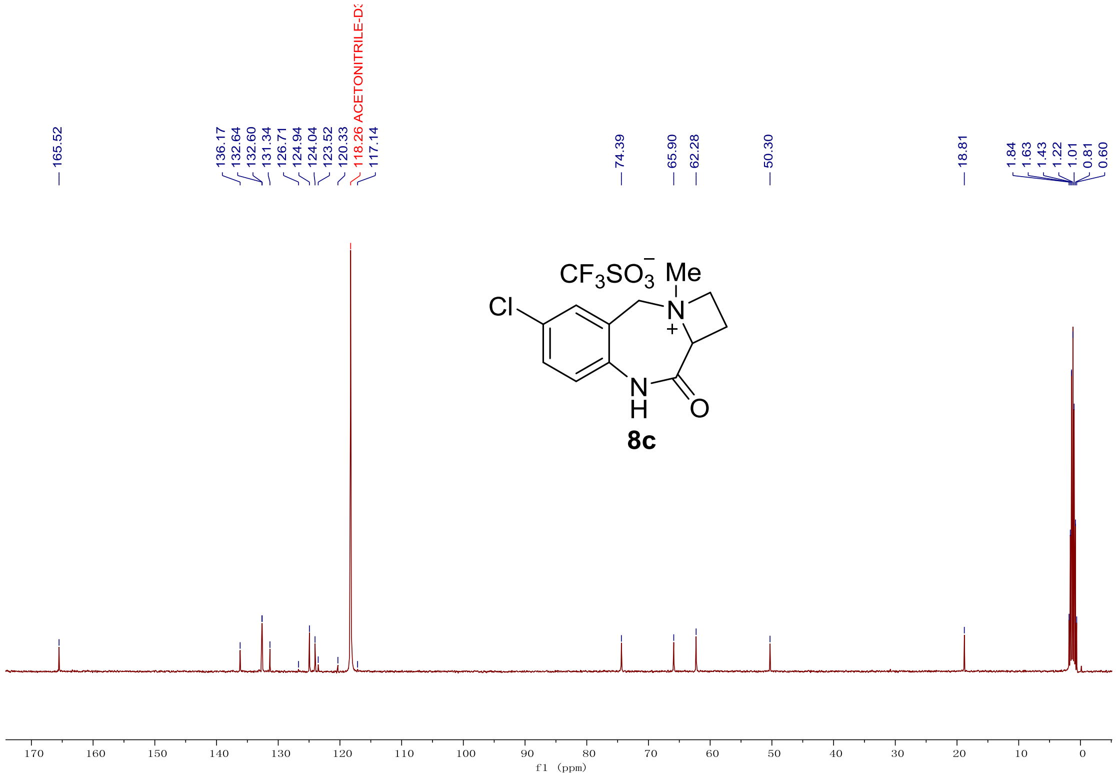

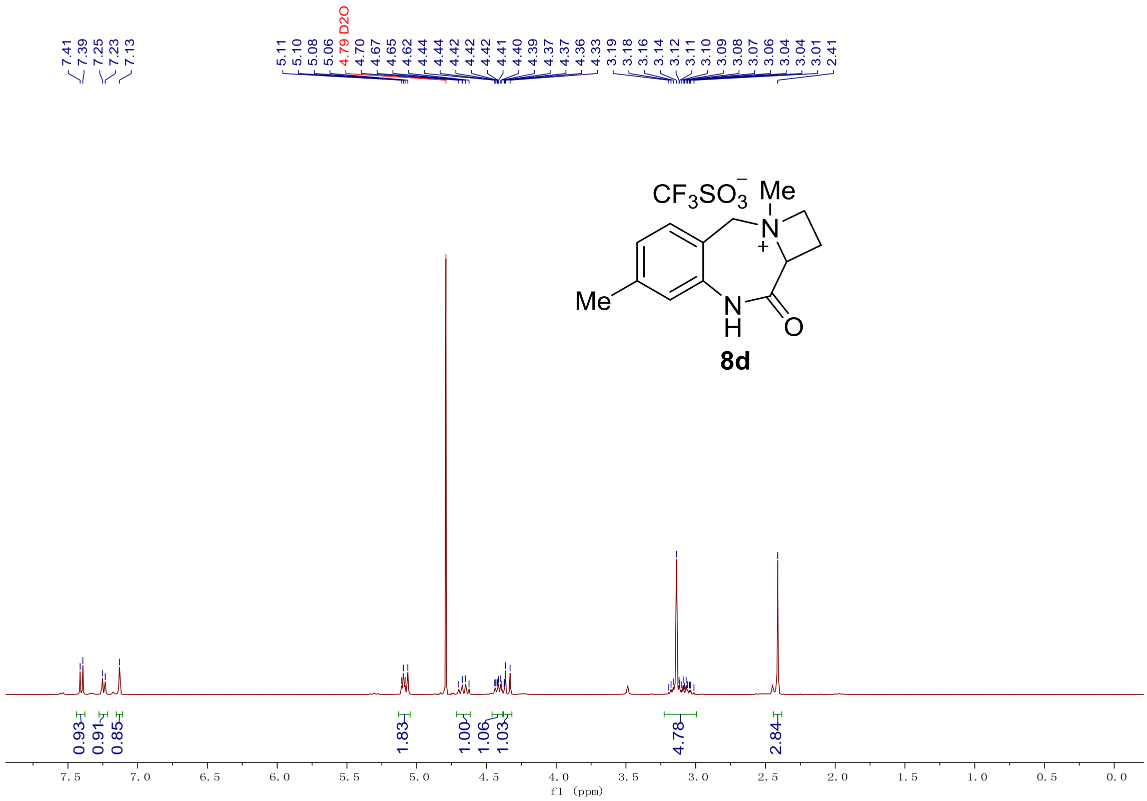

7.41
7.39
7.25
7.23
7.13
5.11
5.10
5.08
5.06
4.79 D2O
4.70
4.67
4.65
4.62
4.44
4.44
4.42
4.42
4.42
4.41
4.40
4.39
4.37
4.37
4.36
4.33
3.19
3.18
3.16
3.14
3.12
3.11
3.10
3.09
3.08
3.07
3.06
3.04
3.04
3.01
2.41
CF3SO3− Me
N
+
Me
N
H
O
8d
0.93
0.91
0.85
1.83
1.00
1.06
1.03
4.78
2.84
7.5 7.0 6.5 6.0 5.5 5.0 4.5 4.0 3.5 3.0 2.5 2.0 1.5 1.0 0.5 0.0
f1 (ppm)

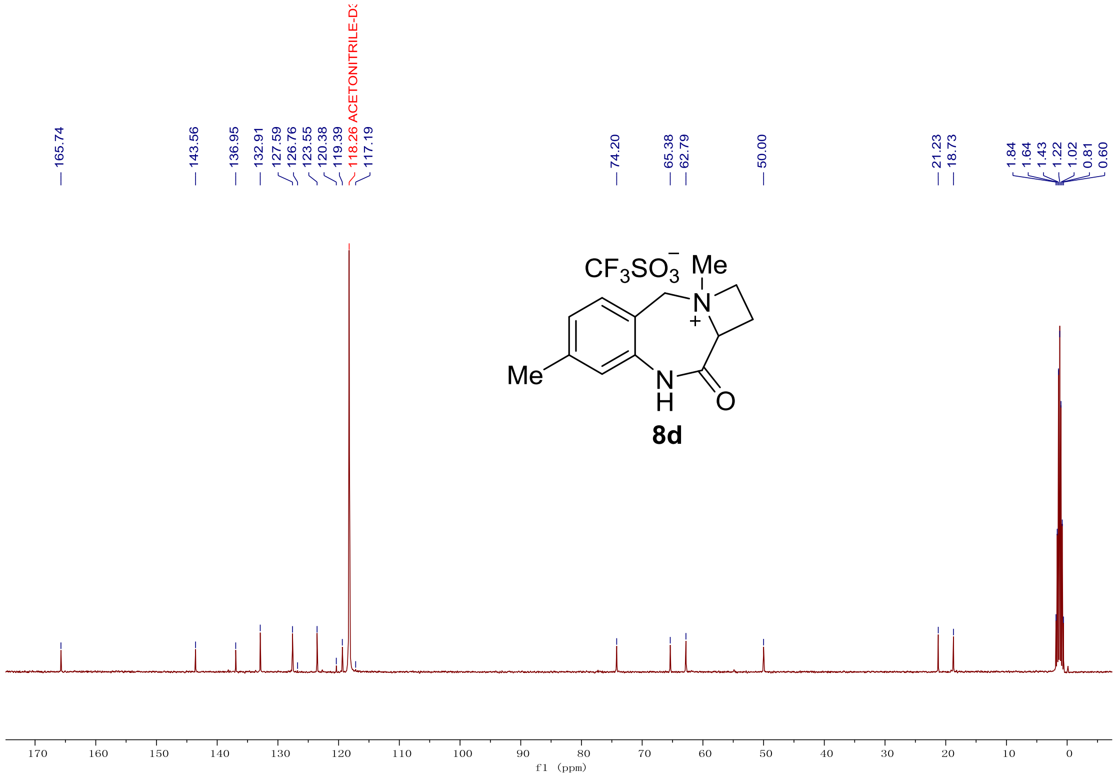

165.74
143.56
136.95
132.91
127.59
126.76
123.55
120.38
119.39
118.26 ACETONITRILE-D3
117.19
74.20
65.38
62.79
50.00
21.23
18.73
1.84
1.64
1.43
1.22
1.02
0.81
0.60
CF3SO3− Me
N
+
Me
N
H
O
8d
170 160 150 140 130 120 110 100 90 80 70 60 50 40 30 20 10 0
f1 (ppm)

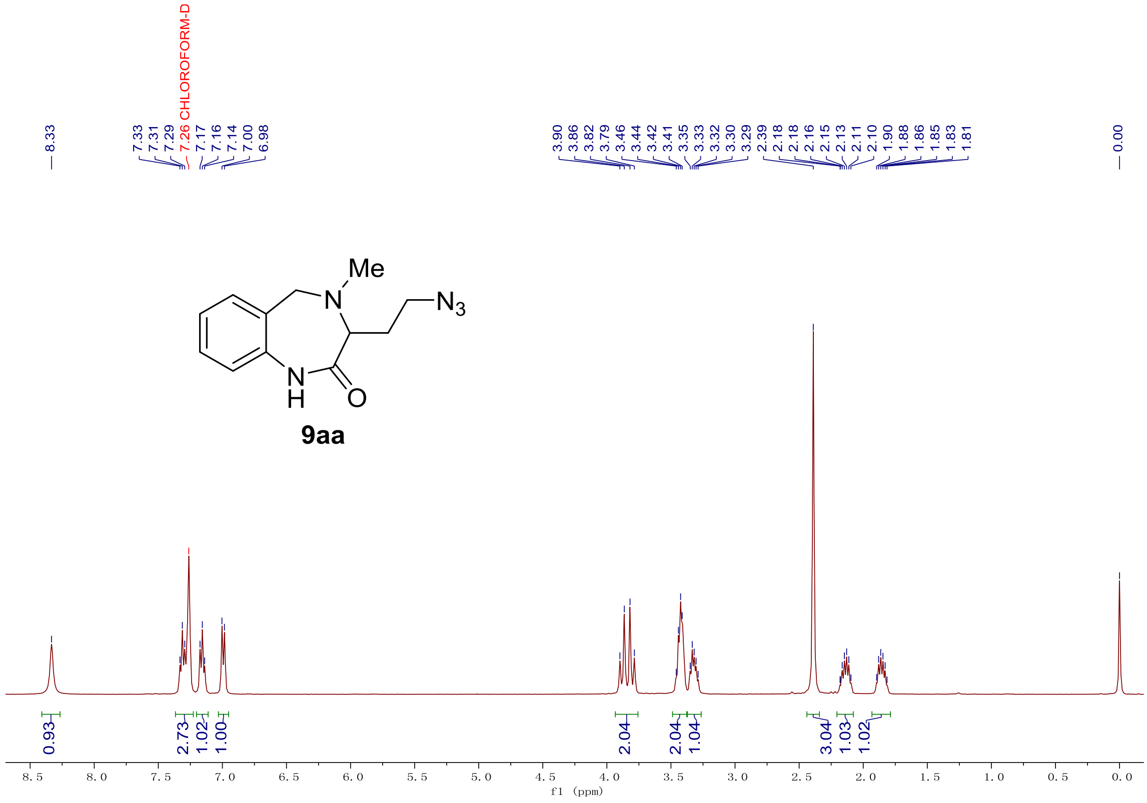

8.33
7.33
7.31
7.29
7.26 CHLOROFORM-D
7.17
7.16
7.14
7.00
6.98
3.90
3.86
3.82
3.79
3.46
3.44
3.42
3.41
3.35
3.33
3.32
3.30
3.29
2.39
2.18
2.18
2.16
2.15
2.13
2.11
2.10
1.90
1.88
1.86
1.85
1.83
1.81
0.00
Me
N
N3
N
H
O
9aa
0.93
2.73
1.02
1.00
2.04
2.04
1.04
3.04
1.03
1.02
f1 (ppm)

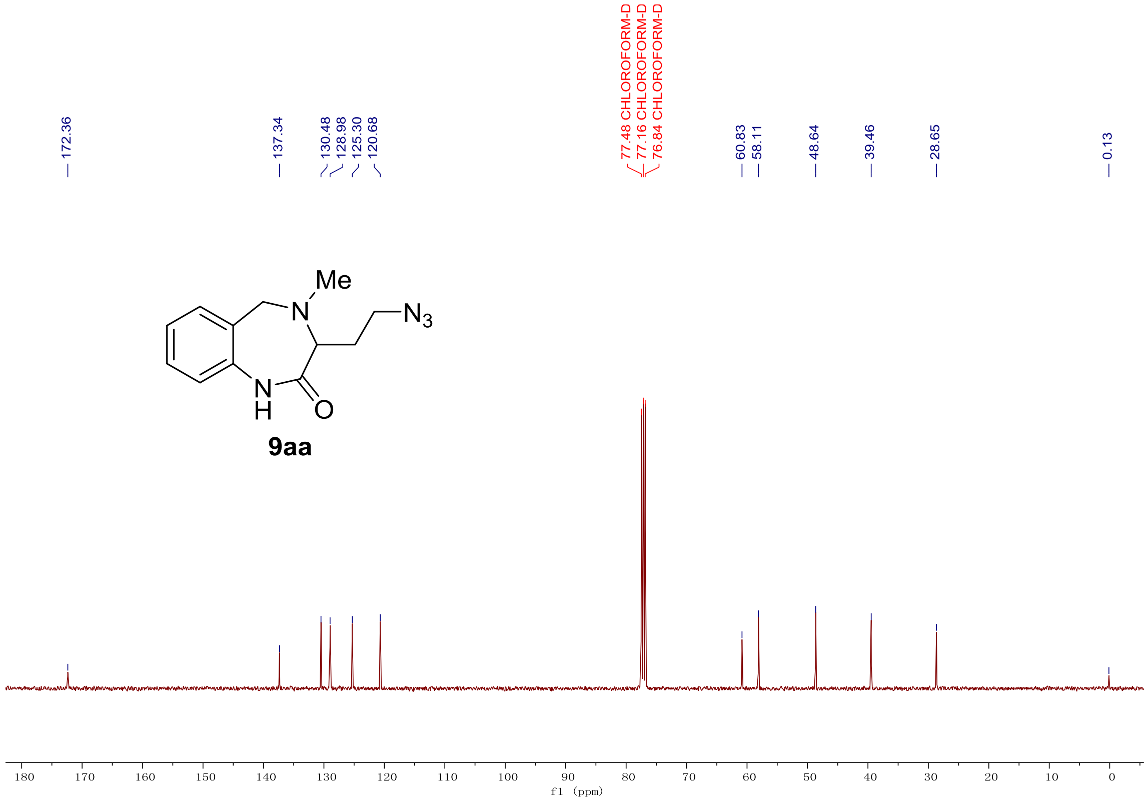

172.36
137.34
130.48
128.98
125.30
120.68
77.48 CHLOROFORM-D
77.16 CHLOROFORM-D
76.84 CHLOROFORM-D
60.83
58.11
48.64
39.46
28.65
0.13
Me
N
N3
N
H
O
9aa
f1 (ppm)

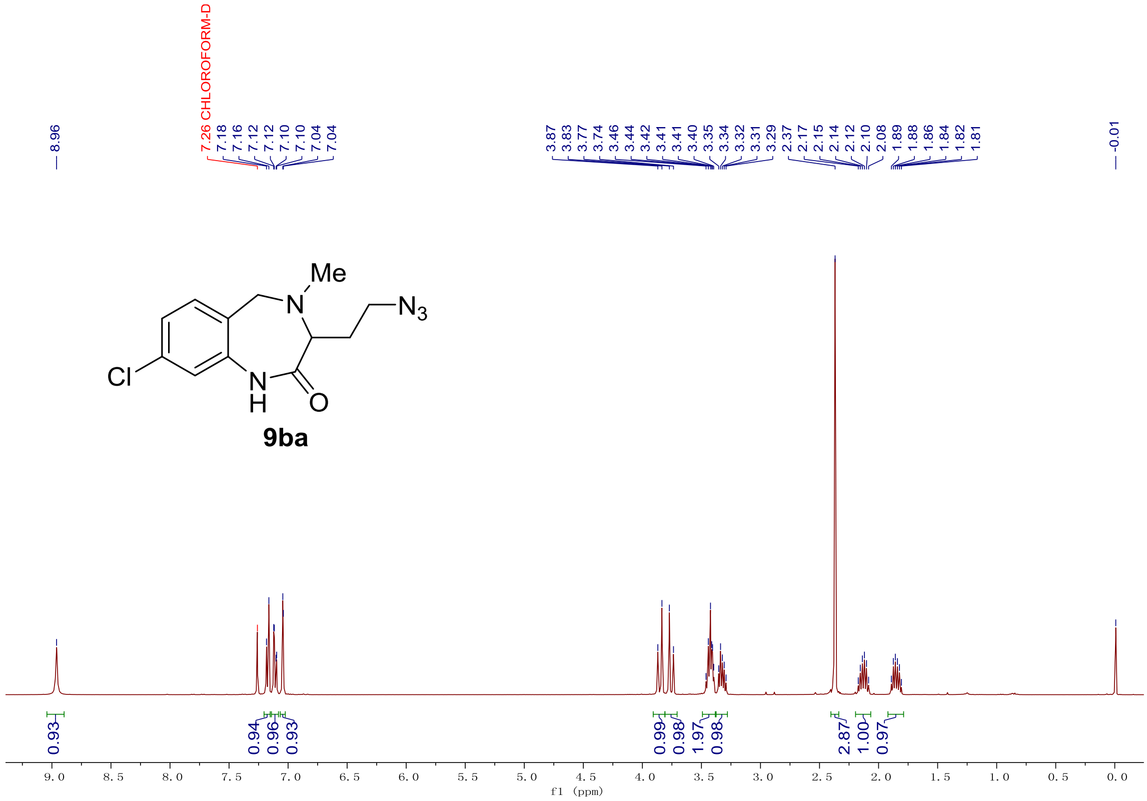
7.26 CHLOROFORM-D
8.96
7.18
7.16
7.12
7.12
7.10
7.10
7.04
7.04
3.87
3.83
3.77
3.74
3.46
3.44
3.42
3.41
3.41
3.40
3.35
3.34
3.32
3.31
3.29
2.37
2.17
2.15
2.14
2.12
2.10
2.08
1.89
1.88
1.86
1.84
1.82
1.81
-0.01
Me
N
N_3
Cl
N
H
O
9ba
0.93
0.94
0.96
0.93
0.99
0.98
1.97
0.98
2.87
1.00
0.97
f1 (ppm)

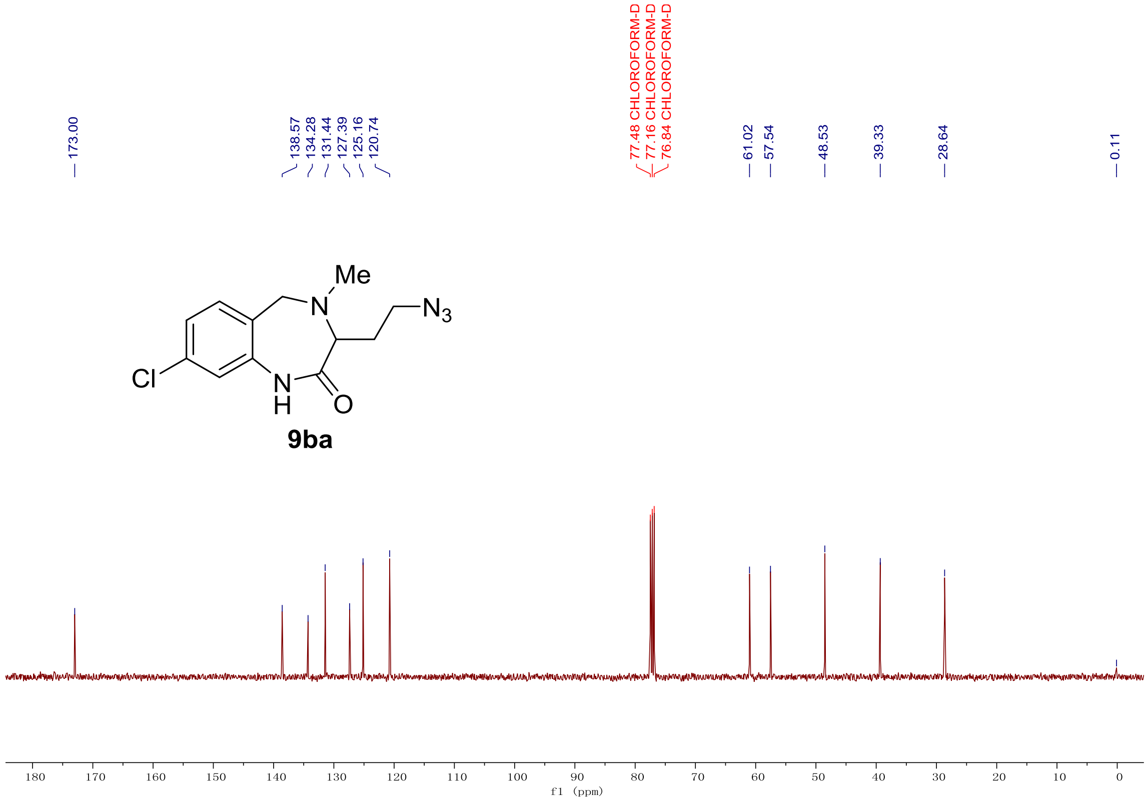
173.00
138.57
134.28
131.44
127.39
125.16
120.74
77.48 CHLOROFORM-D
77.16 CHLOROFORM-D
76.84 CHLOROFORM-D
61.02
57.54
48.53
39.33
28.64
0.11
Me
N
N_3
Cl
N
H
O
9ba
f1 (ppm)

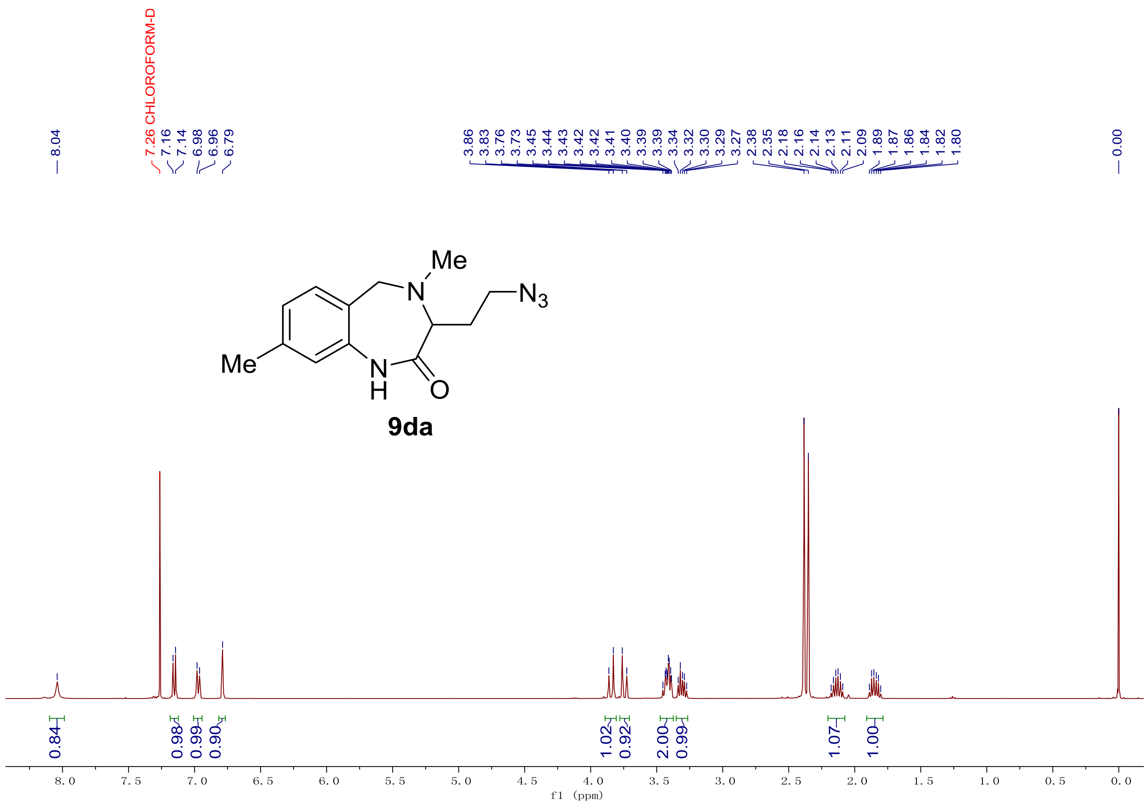

8.04
7.26 CHLOROFORM-D
7.16
7.14
6.98
6.96
6.79
3.86
3.83
3.76
3.73
3.45
3.44
3.43
3.42
3.42
3.41
3.40
3.39
3.39
3.34
3.32
3.30
3.29
3.27
2.38
2.35
2.18
2.16
2.14
2.13
2.11
2.09
1.89
1.87
1.86
1.84
1.82
1.80
0.00
Me
N
N3
Me
N
H
O
9da
0.84
0.98
0.99
0.90
1.02
0.92
2.00
0.99
1.07
1.00
f1 (ppm)

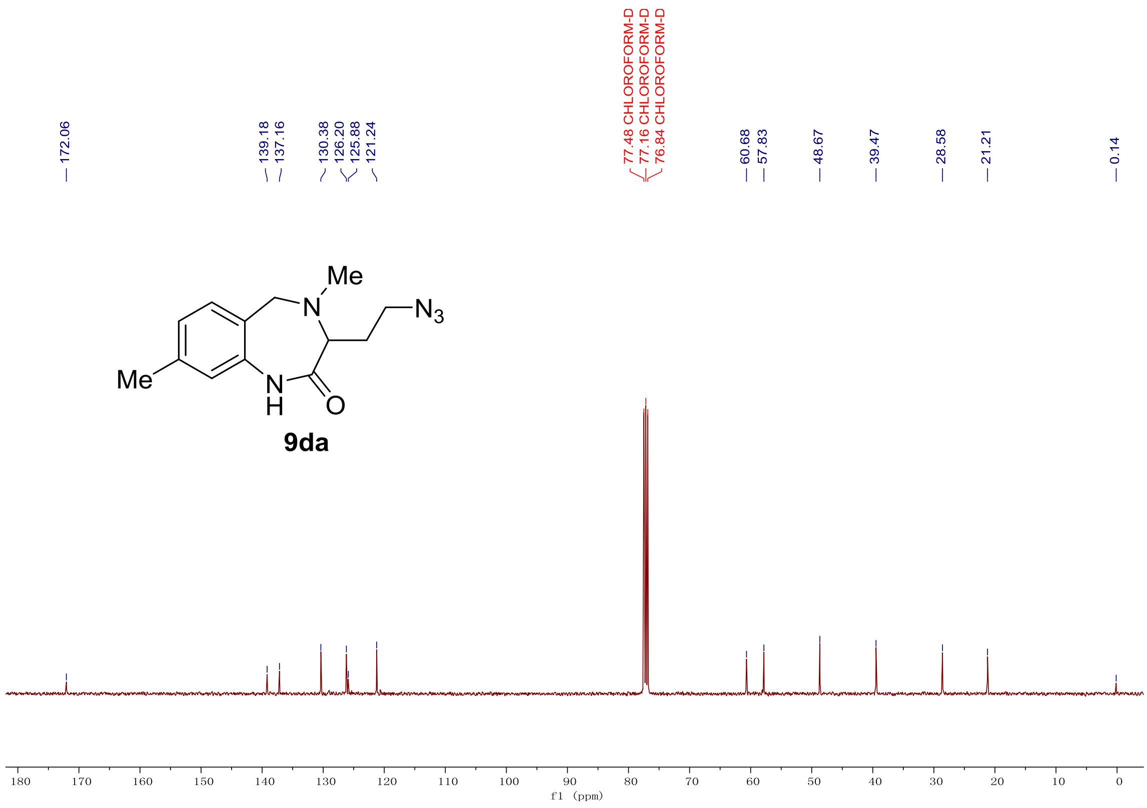

172.06
139.18
137.16
130.38
126.20
125.88
121.24
77.48 CHLOROFORM-D
77.16 CHLOROFORM-D
76.84 CHLOROFORM-D
60.68
57.83
48.67
39.47
28.58
21.21
0.14
Me
N
N3
Me
N
H
O
9da
f1 (ppm)

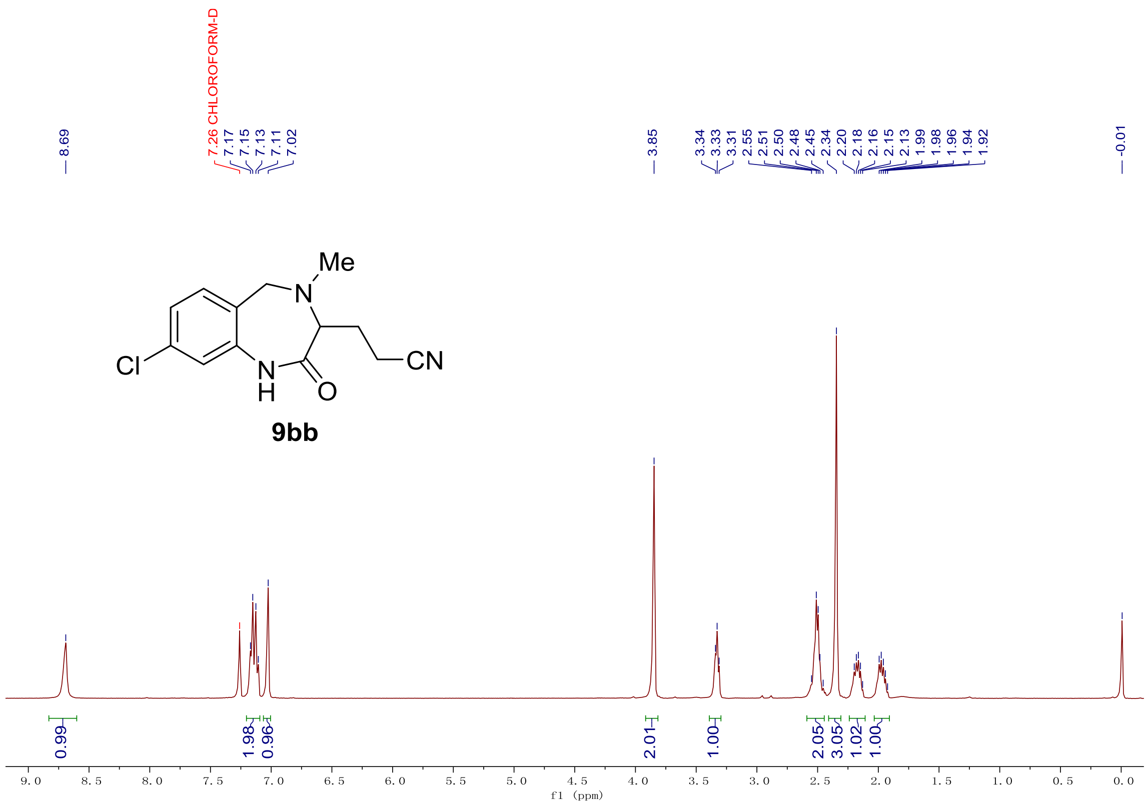
8.69
7.26 CHLOROFORM-D
7.17
7.15
7.13
7.11
7.02
3.85
3.34
3.33
3.31
2.55
2.51
2.50
2.48
2.45
2.34
2.20
2.18
2.16
2.15
2.13
1.99
1.98
1.96
1.94
1.92
-0.01
Me
N
Cl
N
H
O
CN
9bb
0.99
1.98
0.96
2.01
1.00
2.05
3.05
1.02
1.00
f1 (ppm)

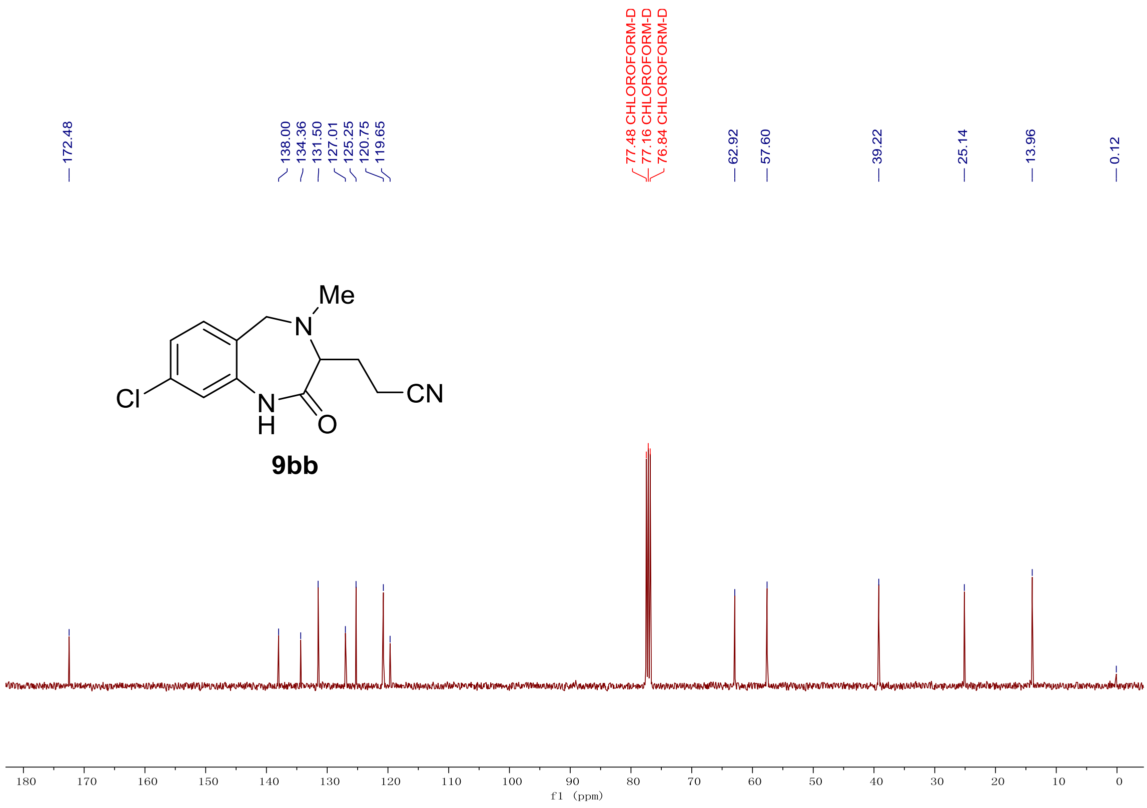
172.48
138.00
134.36
131.50
127.01
125.25
120.75
119.65
77.48 CHLOROFORM-D
77.16 CHLOROFORM-D
76.84 CHLOROFORM-D
62.92
57.60
39.22
25.14
13.96
0.12
Me
N
Cl
N
H
O
CN
9bb
f1 (ppm)

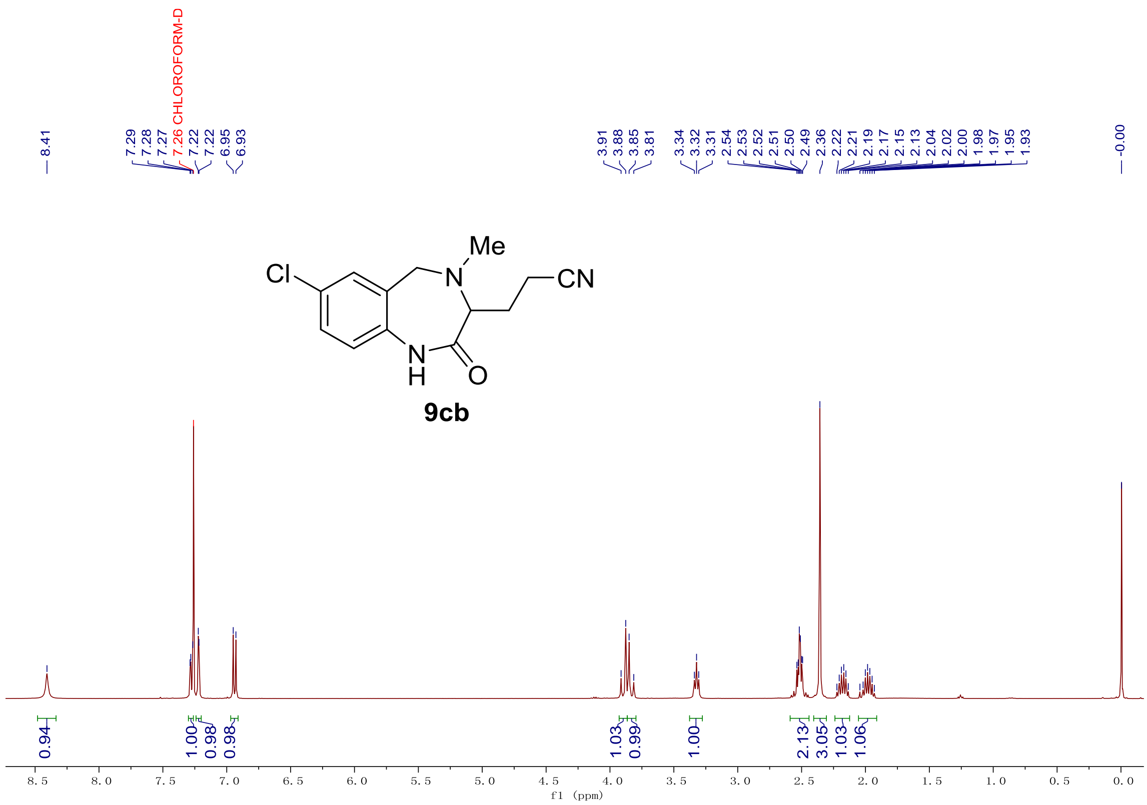
8.41
7.29
7.28
7.27
7.26 CHLOROFORM-D
7.22
7.22
6.95
6.93
3.91
3.88
3.85
3.81
3.34
3.32
3.31
2.54
2.53
2.52
2.51
2.50
2.49
2.36
2.22
2.21
2.19
2.17
2.15
2.13
2.04
2.02
2.00
1.98
1.97
1.95
1.93
-0.00
Me
Cl
N
CN
N
H
O
9cb
0.94
1.00
0.98
0.98
1.03
0.99
1.00
2.13
3.05
1.03
1.06
f1 (ppm)

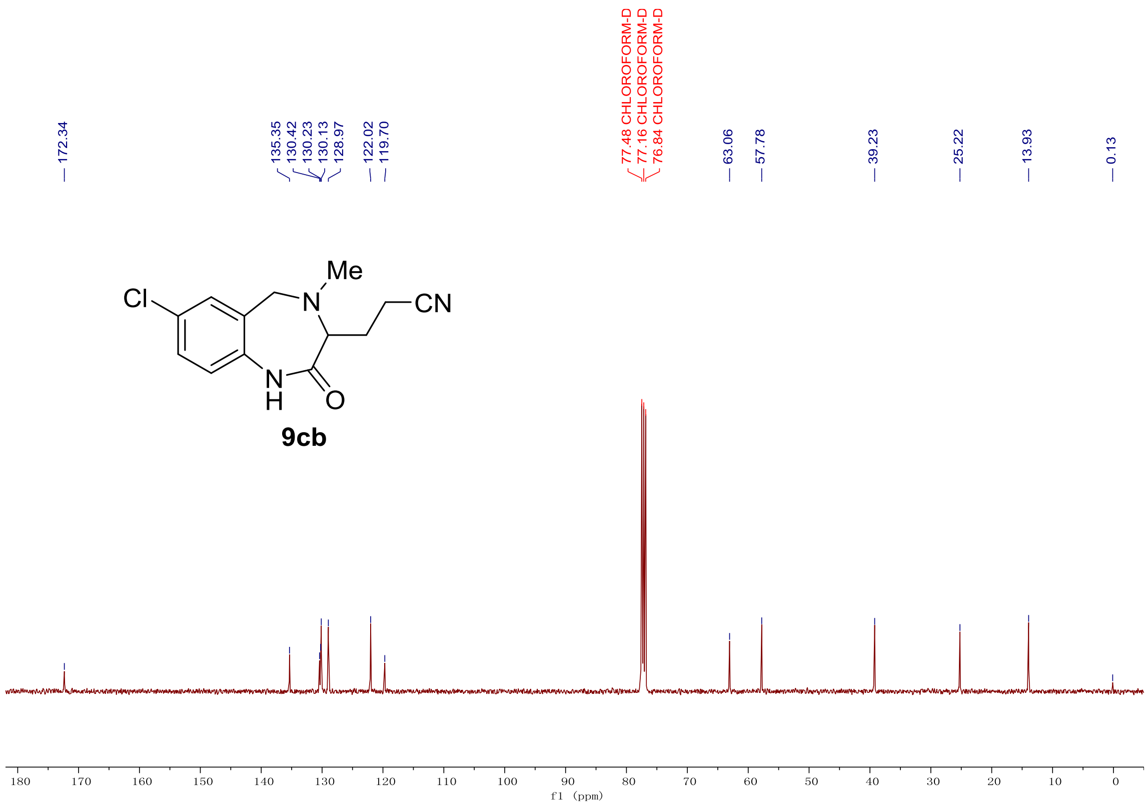
172.34
135.35
130.42
130.23
130.13
128.97
122.02
119.70
77.48 CHLOROFORM-D
77.16 CHLOROFORM-D
76.84 CHLOROFORM-D
63.06
57.78
39.23
25.22
13.93
0.13
Me
Cl
N
CN
N
H
O
9cb
f1 (ppm)

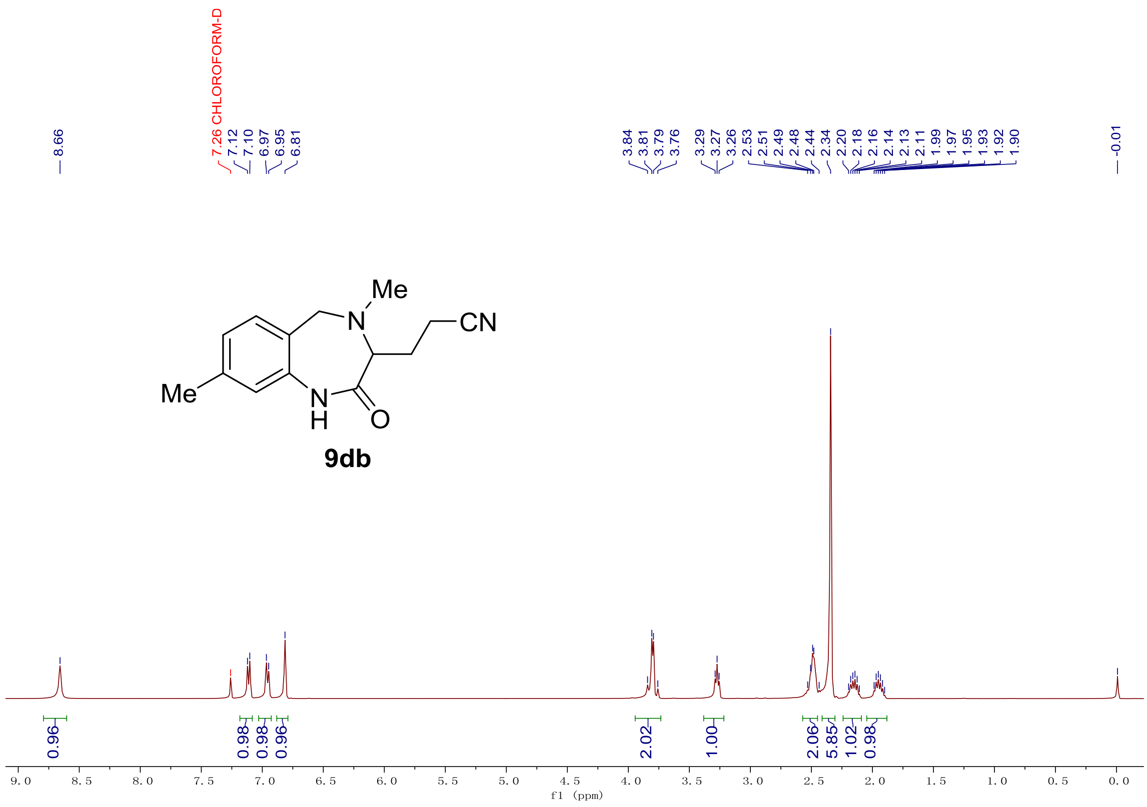
7.26 CHLOROFORM-D
9db
f1 (ppm)

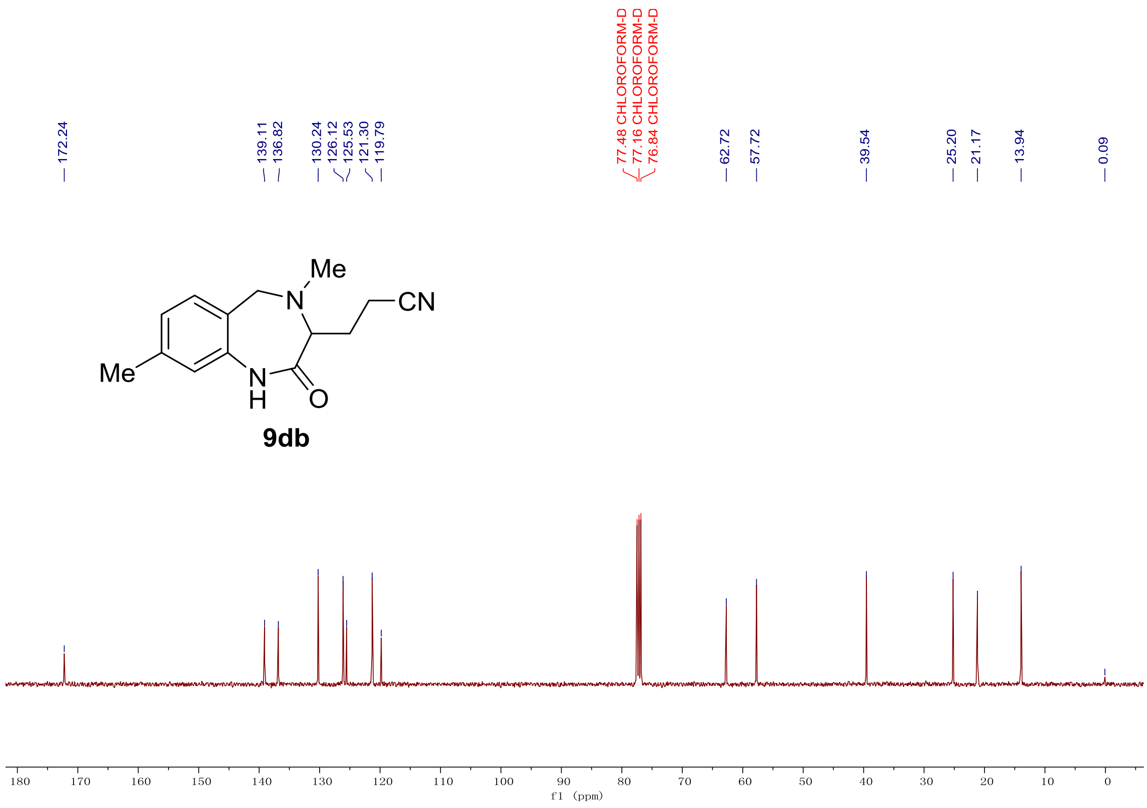
77.48 CHLOROFORM-D
77.16 CHLOROFORM-D
76.84 CHLOROFORM-D
9db
f1 (ppm)

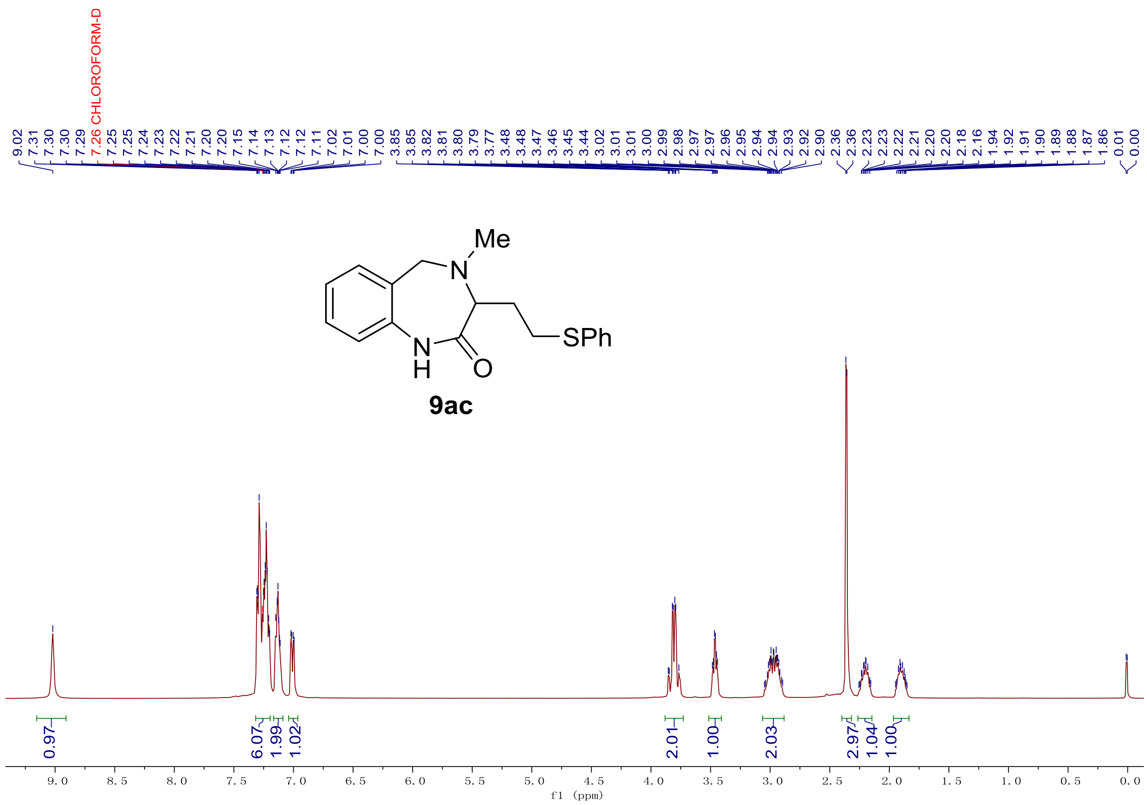
9.02
7.31
7.30
7.29
7.26 CHLOROFORM-D
7.25
7.25
7.24
7.23
7.22
7.21
7.20
7.20
7.15
7.14
7.13
7.12
7.12
7.11
7.02
7.01
7.00
3.85
3.82
3.81
3.80
3.79
3.77
3.48
3.48
3.47
3.46
3.45
3.44
3.02
3.01
3.01
3.00
2.99
2.98
2.97
2.97
2.96
2.95
2.94
2.94
2.93
2.92
2.90
2.36
2.36
2.23
2.23
2.22
2.21
2.20
2.20
2.18
2.16
1.94
1.92
1.91
1.90
1.89
1.88
1.87
1.86
0.01
0.00
Me
N
SPh
N
H
O
9ac
0.97
6.07
1.99
1.02
2.01
1.00
2.03
2.97
1.04
1.00
f1 (ppm)

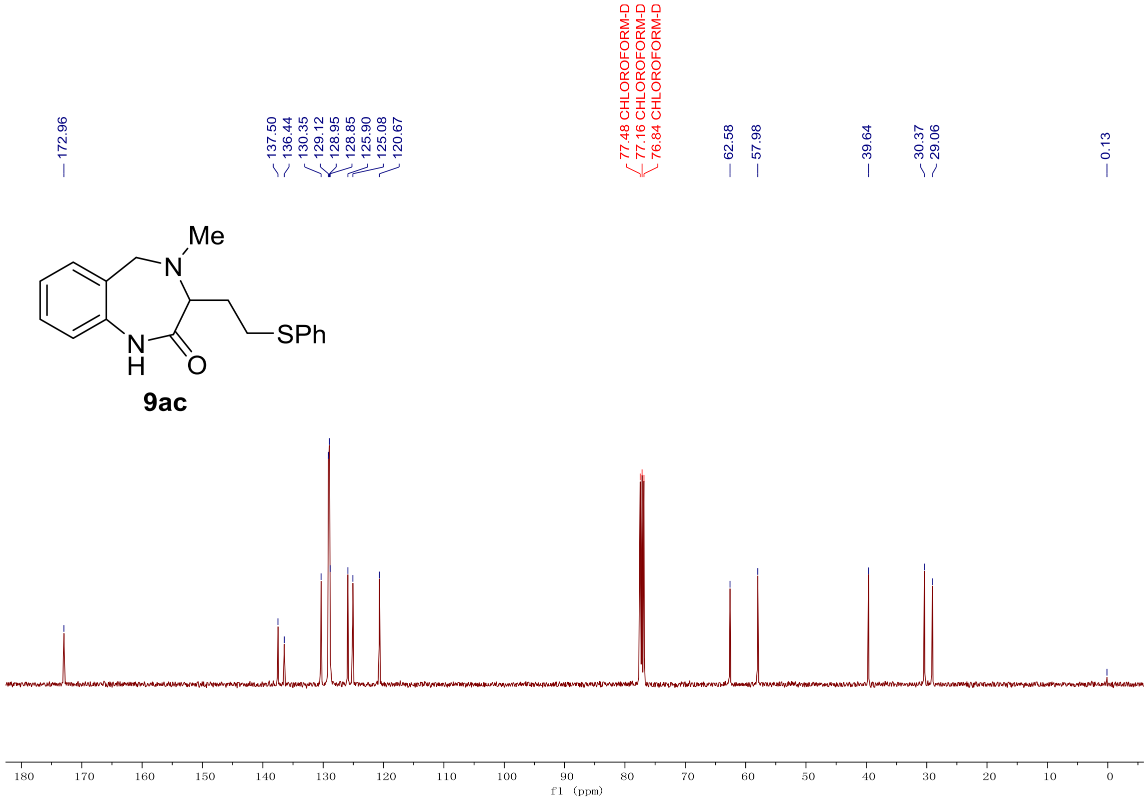
172.96
137.50
136.44
130.35
129.12
128.95
128.85
125.90
125.08
120.67
77.48 CHLOROFORM-D
77.16 CHLOROFORM-D
76.84 CHLOROFORM-D
62.58
57.98
39.64
30.37
29.06
0.13
Me
N
SPh
N
H
O
9ac
f1 (ppm)

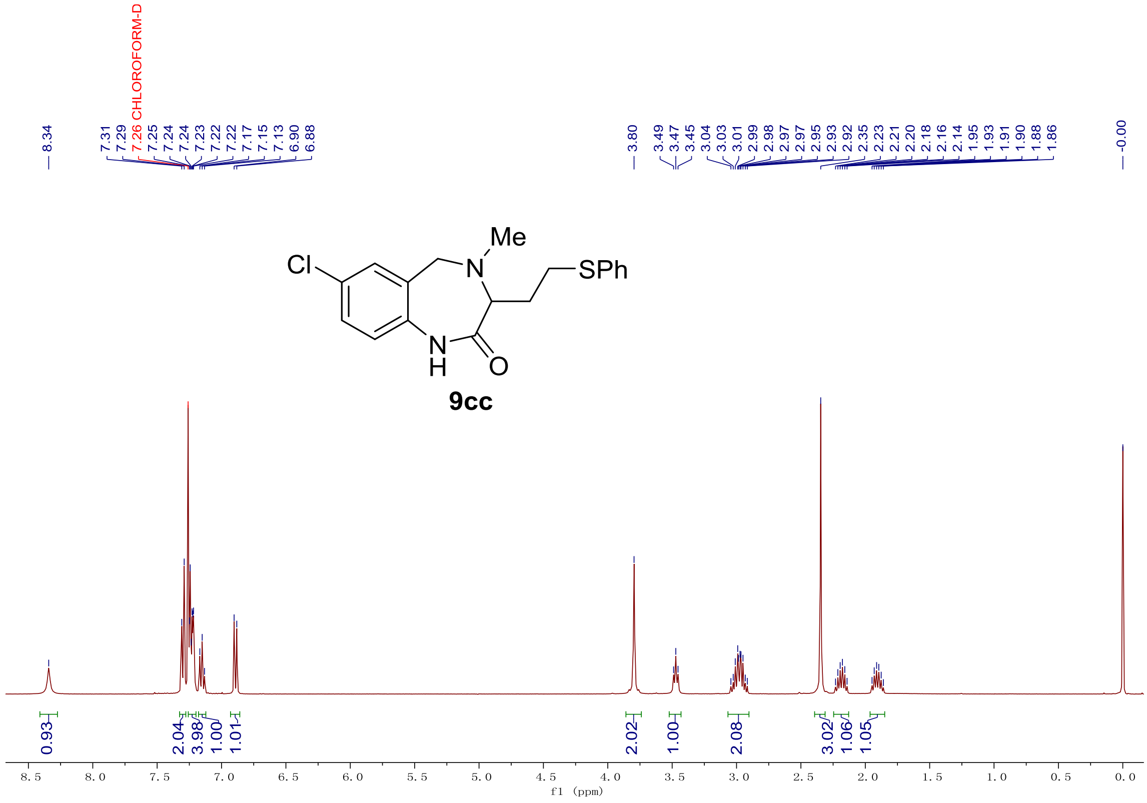
8.34
7.31
7.29
7.26 CHLOROFORM-D
7.25
7.24
7.24
7.23
7.22
7.22
7.17
7.15
7.13
6.90
6.88
3.80
3.49
3.47
3.45
3.04
3.03
3.01
2.99
2.98
2.97
2.97
2.95
2.93
2.92
2.35
2.23
2.21
2.20
2.18
2.16
2.14
1.95
1.93
1.91
1.90
1.88
1.86
-0.00
Me
Cl
N
SPh
N
H
O
9cc
0.93
2.04
3.98
1.00
1.01
2.02
1.00
2.08
3.02
1.06
1.05
f1 (ppm)

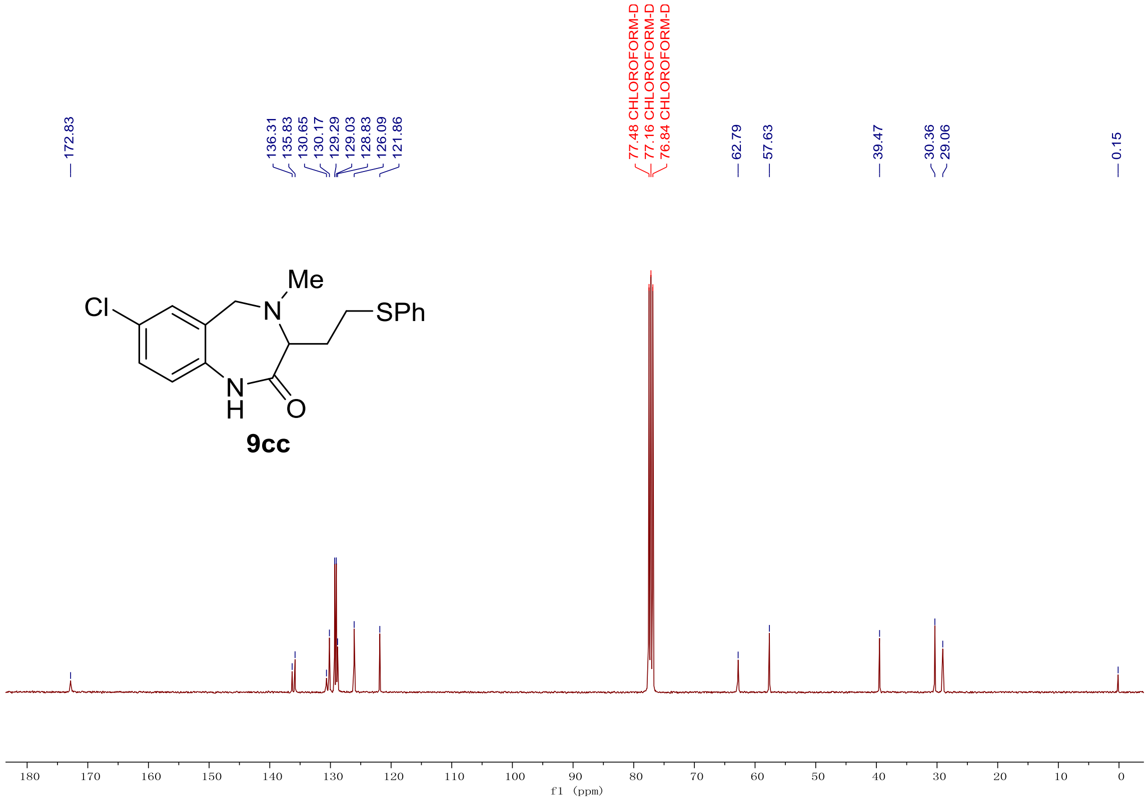
172.83
136.31
135.83
130.65
130.17
129.29
129.03
128.83
126.09
121.86
77.48 CHLOROFORM-D
77.16 CHLOROFORM-D
76.84 CHLOROFORM-D
62.79
57.63
39.47
30.36
29.06
0.15
Me
Cl
N
SPh
N
H
O
9cc
f1 (ppm)

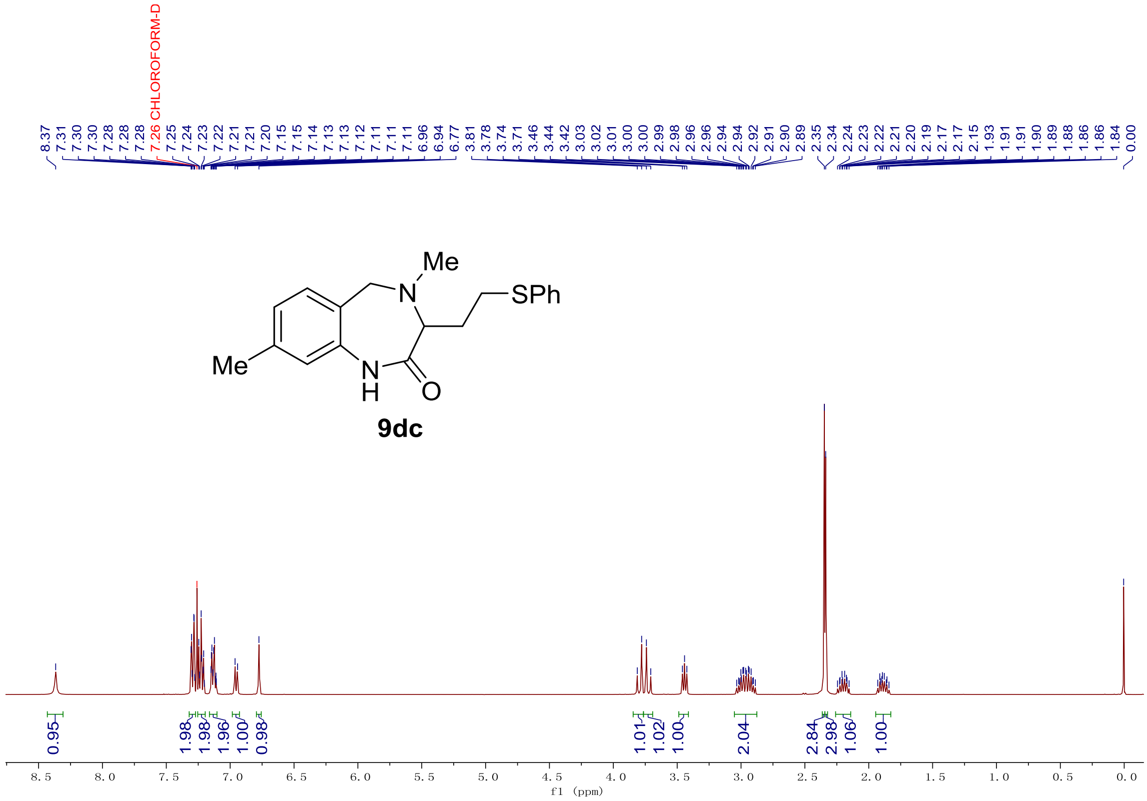
8.37 7.31 7.30 7.28 7.28 7.26 CHLOROFORM-D 7.25 7.24 7.23 7.22 7.21 7.21 7.20 7.15 7.14 7.13 7.13 7.12 7.11 7.11 7.11 6.96 6.94 6.77 3.81 3.78 3.74 3.71 3.46 3.44 3.42 3.03 3.02 3.01 3.00 3.00 2.99 2.98 2.96 2.96 2.94 2.94 2.92 2.91 2.90 2.89 2.35 2.34 2.24 2.23 2.22 2.21 2.20 2.19 2.17 2.17 2.15 1.93 1.91 1.91 1.90 1.89 1.88 1.86 1.86 1.84 0.00
Me
N
SPh
Me
N
H
O
9dc
0.95 1.98 1.98 1.96 1.00 0.98 1.01 1.02 1.00 2.04 2.84 2.98 1.06 1.00
f1 (ppm)

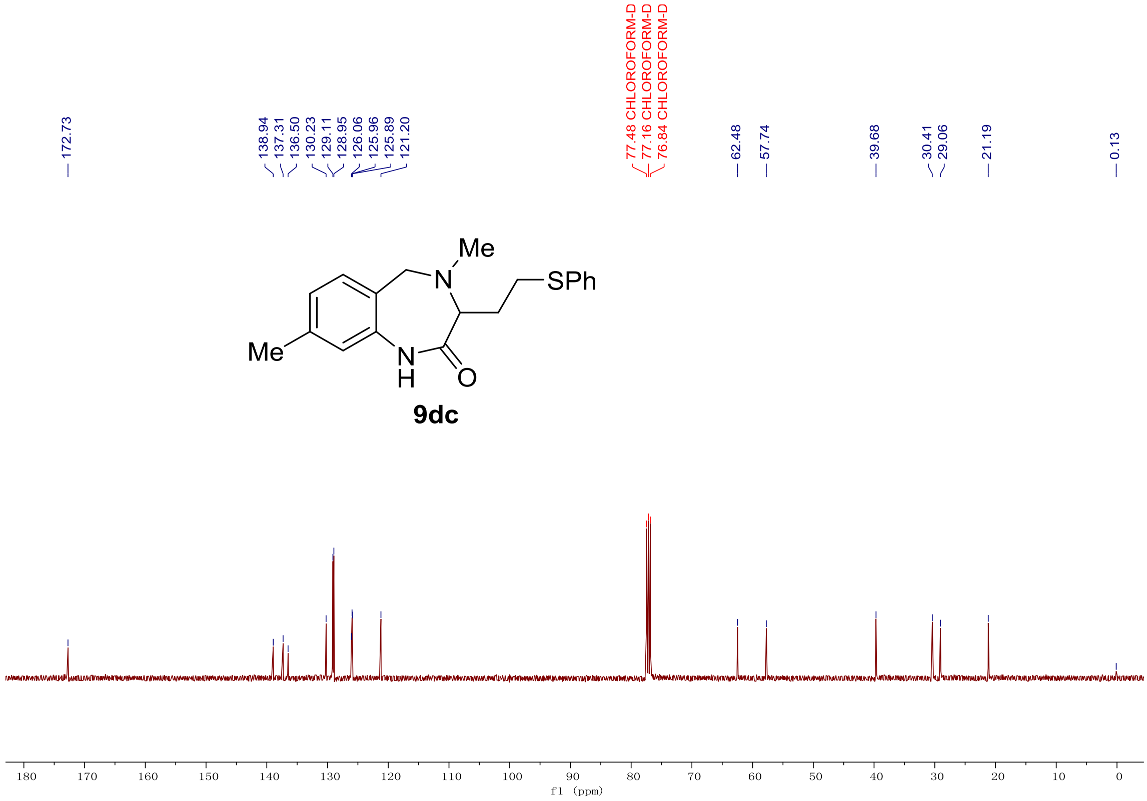
172.73
138.94 137.31 136.50 130.23 129.11 128.95 126.06 125.96 125.89 121.20
77.48 CHLOROFORM-D 77.16 CHLOROFORM-D 76.84 CHLOROFORM-D
62.48 57.74 39.68 30.41 29.06 21.19 0.13
Me
N
SPh
Me
N
H
O
9dc
f1 (ppm)

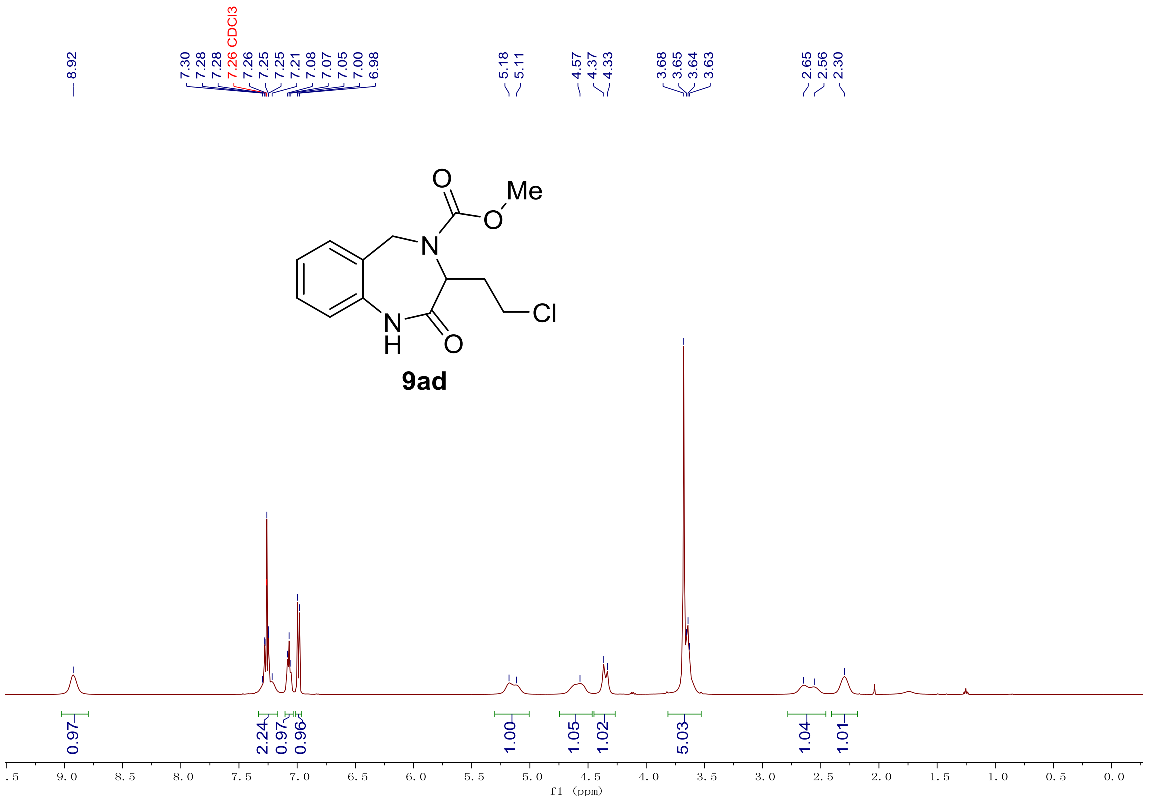
8.92
7.30 7.28 7.28 7.26 CDCl3 7.26 7.25 7.25 7.21 7.21 7.08 7.07 7.05 7.00 6.98
5.18 5.11
4.57 4.37 4.33
3.68 3.65 3.64 3.63
2.65 2.56 2.30
Me
O
N
N
H
Cl
9ad
0.97
2.24 0.97 0.96
1.00
1.05 1.02
5.03
1.04 1.01
f1 (ppm)

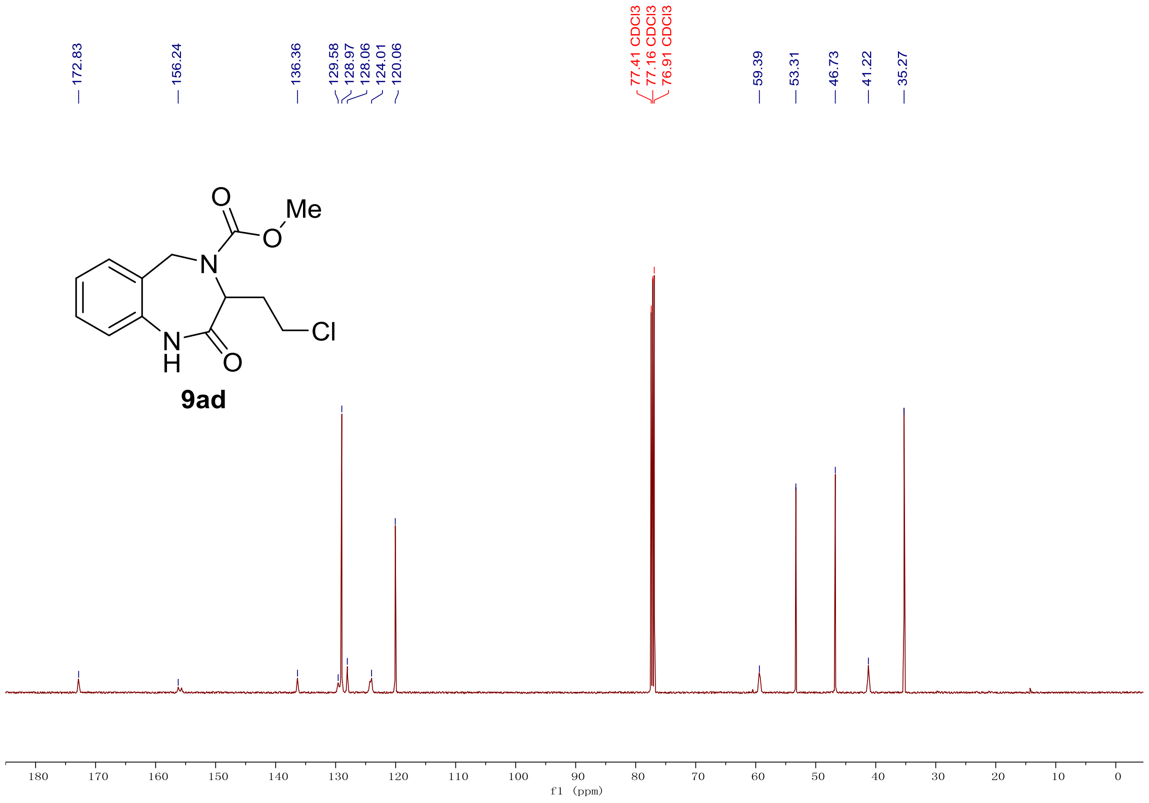
172.83
156.24
136.36
129.58 128.97 128.06 124.01 120.06
77.41 CDCl3 77.16 CDCl3 76.91 CDCl3
59.39
53.31
46.73
41.22
35.27
Me
O
N
N
H
Cl
9ad
f1 (ppm)

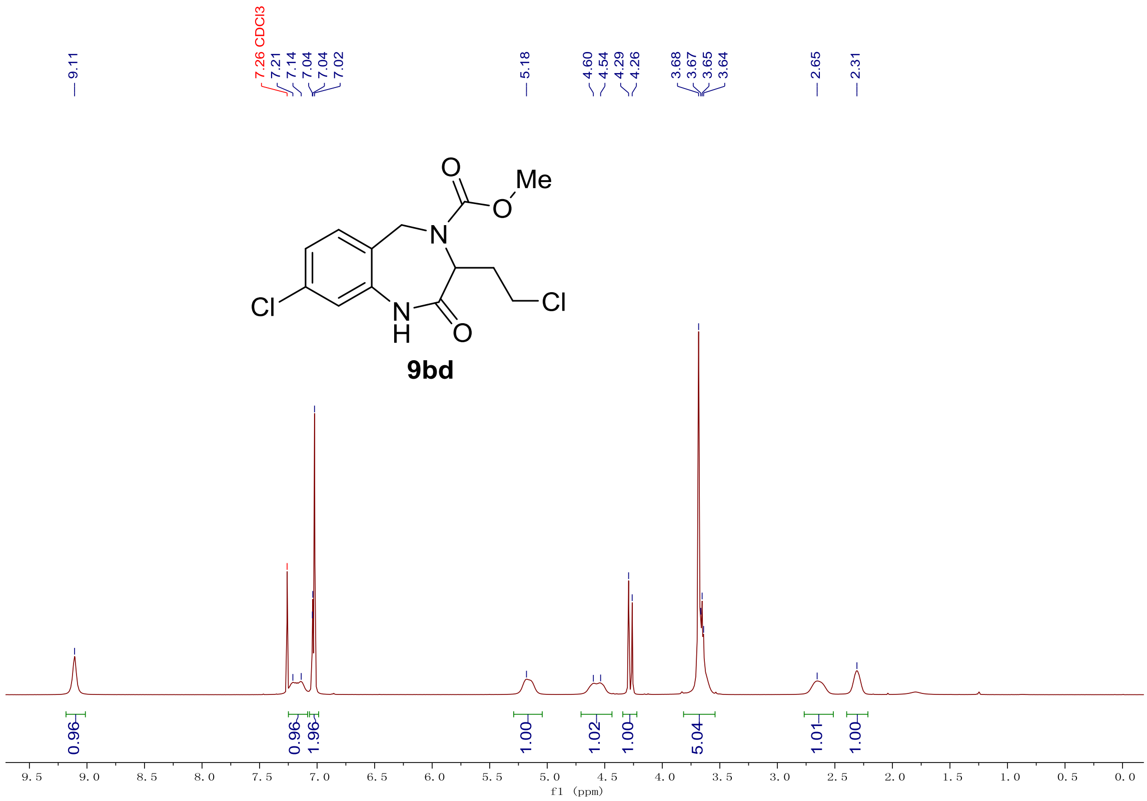
9.11
7.26 CDCl3
7.21
7.14
7.04
7.04
7.02
5.18
4.60
4.54
4.29
4.26
3.68
3.67
3.65
3.64
2.65
2.31
O
Me
O
N
Cl
N
H
O
Cl
9bd
0.96
0.96
1.96
1.00
1.02
1.00
5.04
1.01
1.00
f1 (ppm)

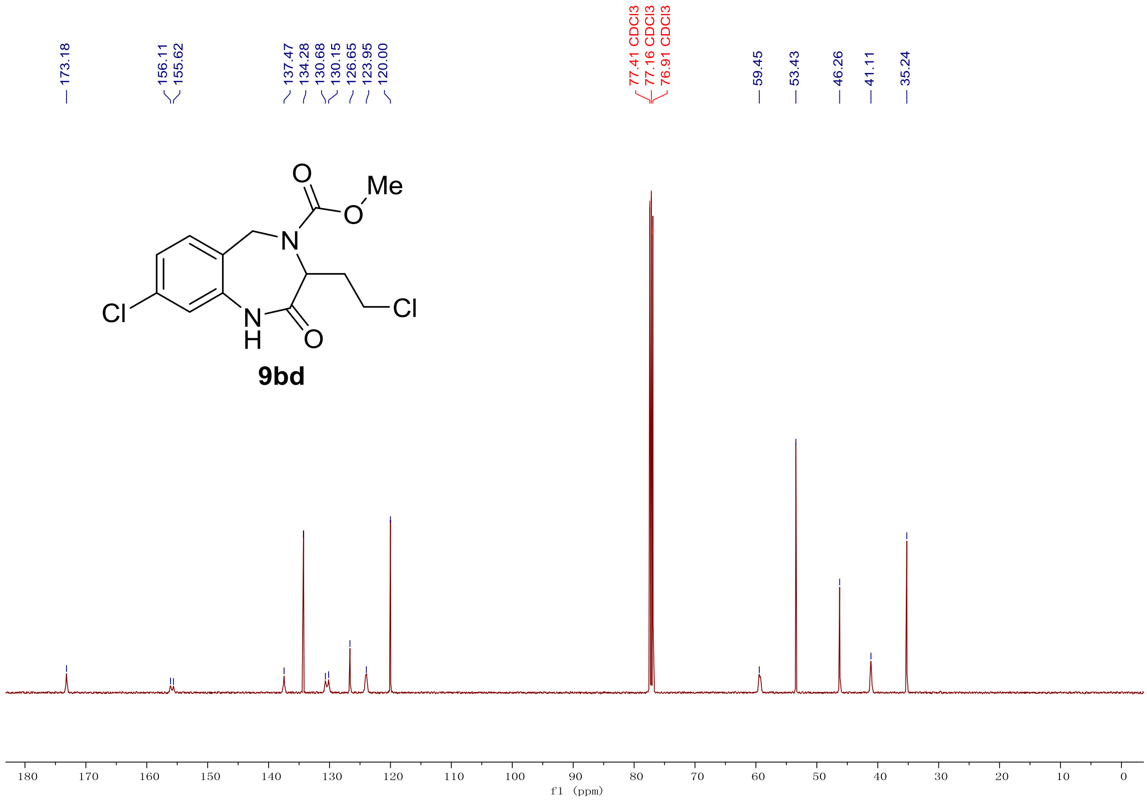
173.18
156.11
155.62
137.47
134.28
130.68
130.15
126.65
123.95
120.00
77.41 CDCl3
77.16 CDCl3
76.91 CDCl3
59.45
53.43
46.26
41.11
35.24
O
Me
O
N
Cl
N
H
O
Cl
9bd
f1 (ppm)

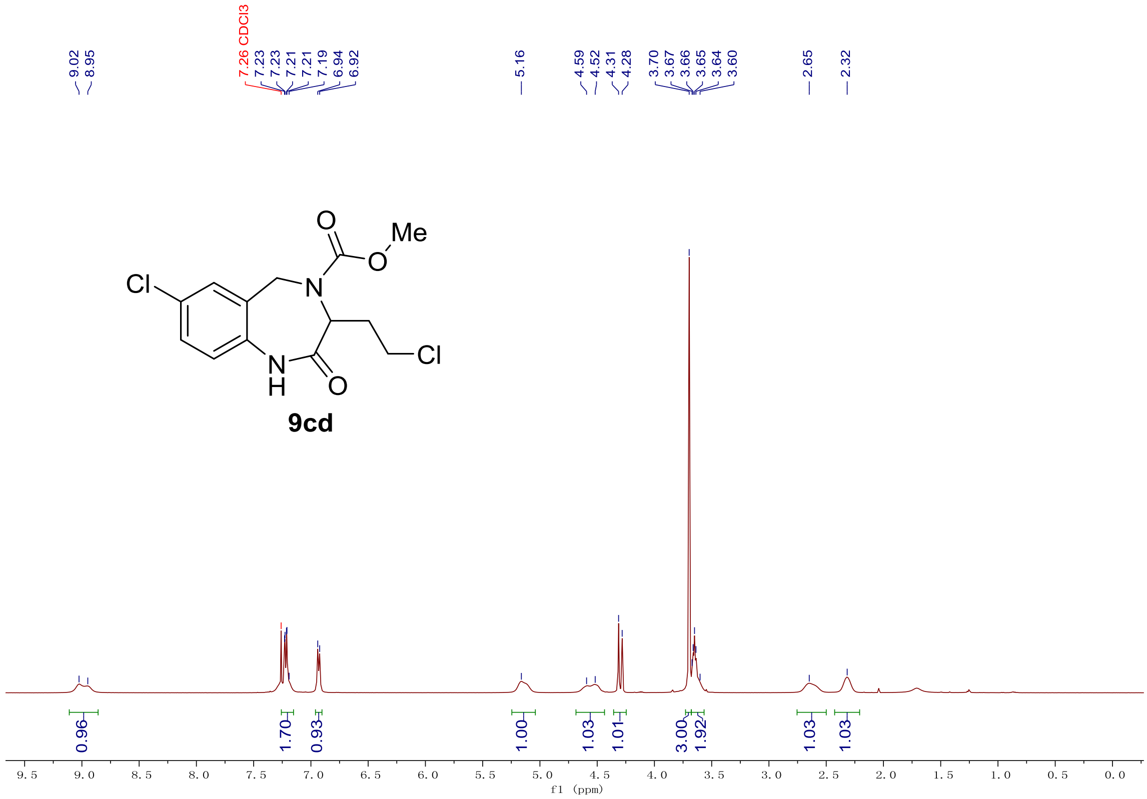
9.02
8.95
7.26 CDCl3
7.23
7.23
7.21
7.21
7.19
6.94
6.92
5.16
4.59
4.52
4.31
4.28
3.70
3.67
3.66
3.65
3.64
3.60
2.65
2.32
Cl
N
O
O
Me
N
H
O
Cl
9cd
0.96
1.70
0.93
1.00
1.03
1.01
3.00
1.92
1.03
1.03
9.5 9.0 8.5 8.0 7.5 7.0 6.5 6.0 5.5 5.0 4.5 4.0 3.5 3.0 2.5 2.0 1.5 1.0 0.5 0.0
f1 (ppm)

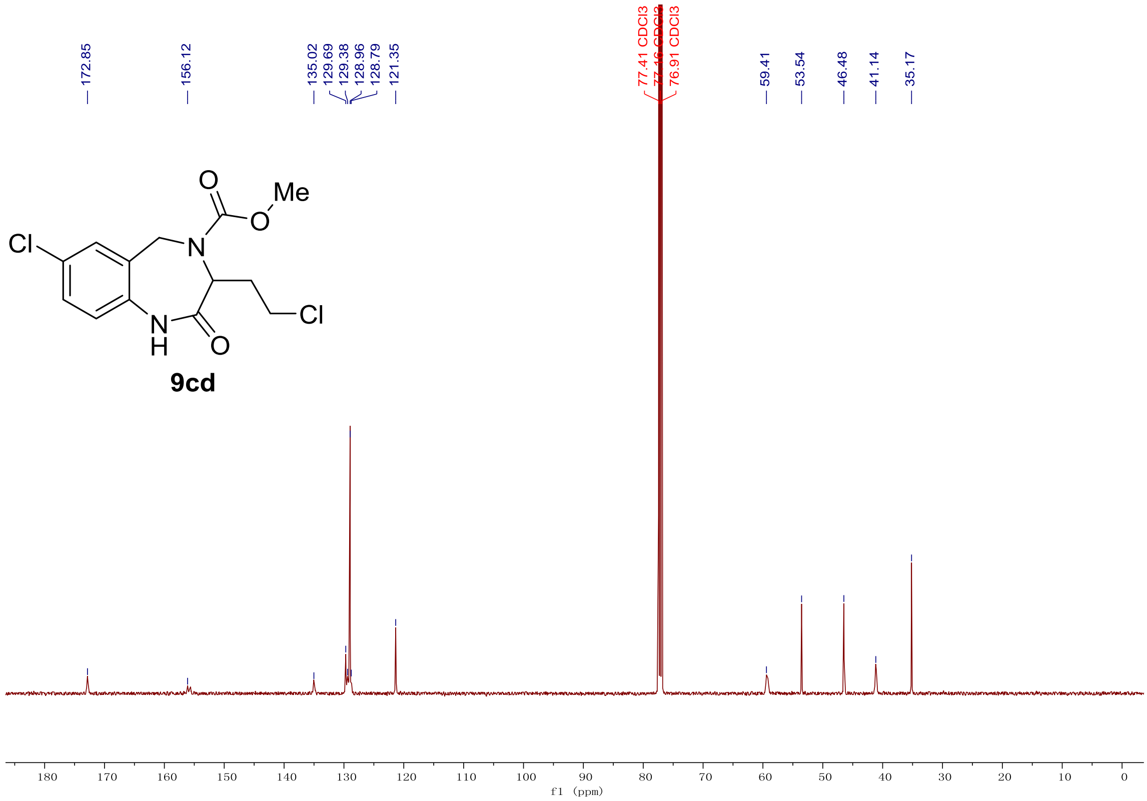
172.85
156.12
135.02
129.69
129.38
128.96
128.79
121.35
77.41 CDCl3
76.91 CDCl3
59.41
53.54
46.48
41.14
35.17
Cl
N
O
O
Me
N
H
O
Cl
9cd
180 170 160 150 140 130 120 110 100 90 80 70 60 50 40 30 20 10 0
f1 (ppm)

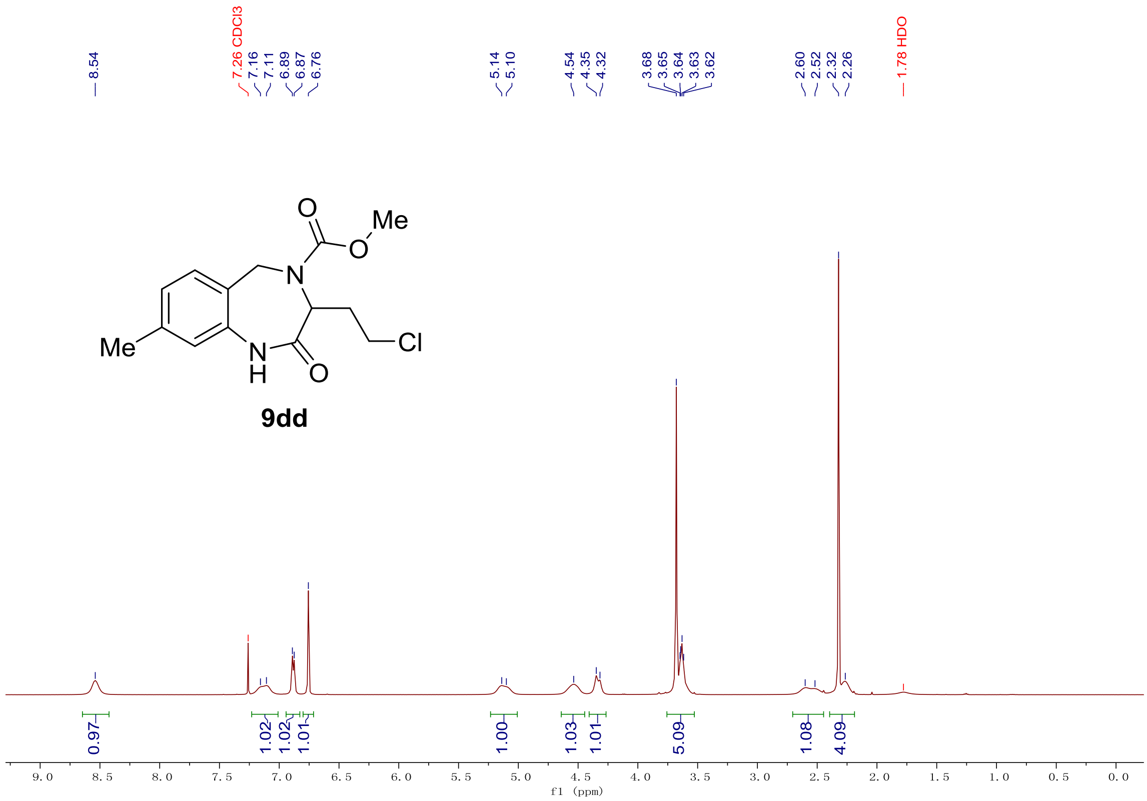
8.54
7.26 CDCl3
7.16
7.11
6.89
6.87
6.76
5.14
5.10
4.54
4.35
4.32
3.68
3.65
3.64
3.63
3.62
2.60
2.52
2.32
2.26
1.78 HDO
Me
O
N
Cl
N
H
9dd
0.97
1.02
1.02
1.01
1.00
1.03
1.01
5.09
1.08
4.09
9.0 8.5 8.0 7.5 7.0 6.5 6.0 5.5 5.0 4.5 4.0 3.5 3.0 2.5 2.0 1.5 1.0 0.5 0.0
f1 (ppm)

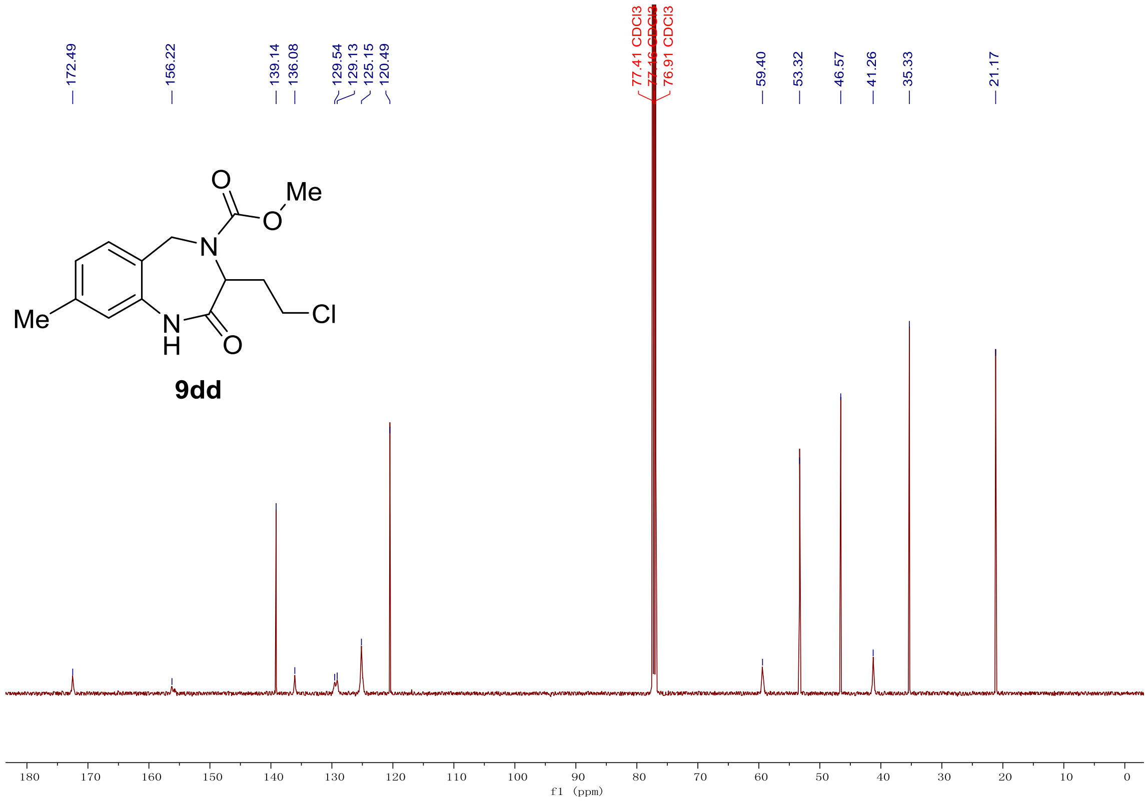
172.49
156.22
139.14
136.08
129.54
129.13
125.15
120.49
77.41 CDCl3
76.91 CDCl3
59.40
53.32
46.57
41.26
35.33
21.17
Me
O
N
Cl
N
H
9dd
180 170 160 150 140 130 120 110 100 90 80 70 60 50 40 30 20 10 0
f1 (ppm)
